# Mechanistic Insights into Molecular Oxygen Reactivity with Late Transition Metal–Hydride Bonds

**DOI:** 10.1021/acs.inorgchem.5c02310

**Published:** 2025-07-13

**Authors:** Diego Sorbelli, Leonardo Belpassi, Paola Belanzoni

**Affiliations:** † Pritzker School of Molecular Engineering, University of Chicago, 5640 South Ellis Avenue, Chicago, Illinois 60615, United States; ‡ CNR Institute of Chemical Science and Technologies “Giulio Natta” (CNR-SCITEC), Via Elce di Sotto 8, 06123 Perugia, Italy; § Department of Chemistry, Biology and Biotechnology,University of Perugia, Via Elce di Sotto 8, 06123 Perugia, Italy

## Abstract

A gold complex, [(^*t*Bu^PCP)­Au–H]^+^ (^*t*Bu^PCP = 2,6-bis­(di-*tert*-butylphosphinomethyl)­benzene), has been recently reported to insert O_2_ into the Au­(III)–H bond, leading to a stable Au­(III)–OOH complex with an observed kinetic behavior sharing similarities with those of previously reported Pd­(II)–H (nonradical) and Pt­(IV)–H (autoaccelerated radical chain) reactions with O_2_. In this work, we computationally investigate, by inclusion of spin–orbit coupling (SOC) effects, along the adiabatic PES, this elusive reaction mechanism in connection with the Au­(III)–H bond nature and in comparison with recent case studies involving isostructural Pd­(II)–H ([(^*t*Bu^PCP)­Pd–H]) or different ligand supported Au­(III)–H ([(CNC)­Au–H], CNC = 2,6-bis­(alkylimidazol-2-ylidene)-pyridine) bonds. The M–H (M = Au, Pd) bonds in these complexes are shown to be mainly of electron-sharing nature, featuring, however, a decreasing degree of M­(δ+)–H­(δ−) polarization in the order [(^*t*Bu^PCP)­Pd–H] > [(^*t*Bu^PCP)­Au–H]^+^ > [(CNC)­Au–H], which we propose to be related to their reactivity with dioxygen, with the M–H bond displaying no reactivity ([(CNC)­Au–H]), a radical chain ([(^*t*Bu^PCP)­Au–H]^+^), and a nonradical ([(^*t*Bu^PCP)­Pd–H]) reactivity. The decisive factors in dictating the M–H bond polarity and, consequently, the preferred pathway lie in the nature of both the ligand and the metal, demonstrating how the fine-tuning of the electronic structure of these complexes causes mechanistic pathways to diverge.

## Graphical abstract

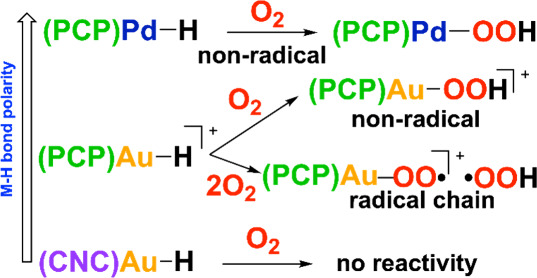

## Introduction

The activation of molecular oxygen and its use as an oxidant in chemical synthesis remains one of the major research challenges in catalysis.^1,2^ Significant environmental and economic benefits, particularly in large-scale industrial processes, such as the ready availability of O_2_ and the absence of environmentally harmful byproducts, are among the attractive features of such processes. However, widespread use of oxygen requires the development of catalytic strategies to produce useful and selectively oxidized products, and to date, it has been hampered by our limited understanding of the mechanisms by which O_2_ reacts with transition metals. The reactions of O_2_ with late transition metal hydride species are of particular interest due to their relevance in oxidation processes, including biological oxidation reactions,^3–5^ as these metal centers are better poised to release oxygenated products. Although metal hydrides are implicated in many catalytic transformations,^6–11^ the mechanisms of their fundamental reaction with O_2_ are poorly understood. The first monomeric gold hydride, [(IPr)­Au­(I)–H] (IPr = 1,3-bis­(2,6-diisopropylphenyl)­imidazole-2-ylidene), was reported in 2008 by Sadighi^12^, and later on, Bochmann^13^ showed its reactivity with O_2_ to give [(IPr)­Au­(I)–OOH]. This spin-forbidden Au­(I)–H/O_2_ insertion reaction (triplet spin state reactants/singlet spin state product) was demonstrated by some of us to be accurately described only by calculations including spin orbit coupling (SOC) effects along the adiabatic potential energy surface (PES).^14,15^ Our computational studies supported an oxidative addition/recombination mechanism, revealing differences with respect to Pd­(II)–H bond in complexes bearing two N-heterocyclic carbene ligands, for which O_2_ insertion was reported to occur through a hydrogen abstraction mechanism in the triplet PES with a pure spin transition state.^16^

Recently, additional homogeneous catalytic systems involving palladium and gold complexes have appeared in the literature, opening new opportunities for efficient and selective aerobic oxidation chemistry. The first observation of molecular oxygen insertion into a Pd­(II)–H bond for a palladium complex supported by a diphosphine pincer ligand, [(^*t*Bu^PCP)­Pd–H] (^*t*Bu^PCP = 2,6-bis­(di-*tert*-butylphosphinomethyl) benzene) was reported by Goldberg and co-workers,^17^ yielding a relatively stable hydroperoxo product [(^*t*Bu^PCP)­Pd–OOH], which was crystallographically characterized. Kinetic studies report that the Pd–H bond cleavage should be implied in the rate-determining step, and results of experimental studies with radical inhibitors and light suggest that the reaction does not proceed by a radical chain mechanism. Reactivity toward molecular oxygen has been reported by Bochmann and co-workers^18^ for a family of Au­(III) hydrides, of the type [(CC)­AuH­(PR_3_)] (R = Me, Ph, *p*-tolyl; CC = 4,4′-di-*tert*-butylbiphenyl-2,2′-diyl), where O_2_ insertion into the Au­(III)–H bond to give [AuOOH­(PR_3_)] insertion intermediate has been suggested. Mechanistic details are not clear from the experiment. Interestingly, this paper, reporting on the reactions of these complexes with alkynes, also investigates the role played by trans-influence on hydride reactivity. On the basis of detailed quantum-chemical calculations and analysis including relativistic spin–orbit coupling effects, a linear relationship could be established between the computed Au–H bond distances, the hydridic character of the Au–H bond, and the hydride NMR chemical shifts. Strong *trans* effect ligands, such as C^–^ in [(CCN)­AuH], raise the deshielding σ­(Au–H) orbital and increase the energetic separation from orbitals with shielding Au­(d_π_)-type MO, resulting in deshielding (positive chemical shift), whereas, in [(CNC)­AuH], σ­(Au–H) and Au­(d_π_) are close in energy, so that the hydride shift is dominated by the shielding SOC contribution. The resulting ^1^H hydride shifts are found to correlate linearly with the DFT optimized Au–H distances and Au–H bond covalency. Interestingly, a strong connection between molecular oxygen and alkyne reactivity can be observed. For example, the Au­(III) hydrides that are found to react with O_2_ in ref 18 are also the same that react with activated alkynes such as dimethylacetylene-biscarboxylate, DMAD, an issue which deserves to be investigated in a more general perspective. More recently, Goldberg and co-workers reported an analogous facile dioxygen insertion into an Au­(III)–H bond for a gold complex supported by the same diphosphine pincer ligand [(^*t*Bu^PCP)­Au–H]^+^ (with OTf^–^ as counterion), similarly forming a stable hydroperoxo product [(^*t*Bu^PCP)­Au–OOH]^+^, which was structurally characterized.^19^ For the gold hydride [(^*t*Bu^PCP)­Au–H]^+^, results of experimental studies are consistent with an autoaccelerating radical chain mechanism and, although similarities to Pd­(II)–H (and Pt­(IV)–H) reactions with O_2_ have been observed, the kinetic behavior was found to be not fully consistent with any known O_2_ insertion mechanism, sharing features of both the Pd­(II)–H and Pt­(IV)–H^20,21^ O_2_ insertion reactivity. Interestingly, for the first reported monomeric Au­(III)–H bond in the [(CNC)­Au–H] complex, bearing a rigid doubly cyclometalated 2,6-diphenylpyridine CNC pincer ligand framework, no reaction with O_2_ under thermal or photolytic conditions was observed.^22,23^ Instead, isocyanide insertion into the Au­(III)–H bond in the same complex, giving the first example of gold iminoformyl complexes, was reported,^24^ which is initiated by a catalytic amount of radicals. DFT calculations indicate that the process begins with the abstraction of an H· radical, forming [(CNC)­Au­(II)·] radicals that react with the substrate in a barrierless, exergonic step. The same mechanism cited in ref 24 is also active for alkyne hydroauration with [(CNC)­AuH] complex.^25^ The homolytic breaking of the [(CNC)­Au–H] bond, which results in a radical mechanism, thus requires the presence of suitable radicals. In addition, the trans ligand donor strength can still affect the reactivity (see, for instance, refs 18 and 26).

Computational DFT studies on oxygen insertion into Pd­(II)–H bond of [(^*t*Bu^PCP)­Pd–H] are available in the literature,^16,27–31^ pointing to a hydrogen atom abstraction mechanism possibly involving the formation of a Pd­(I)·/HOO· (triplet) radical pair, which very easily proceeds to form the singlet palladium hydroperoxo species. In ref 29, Sicilia and co-workers further analyzed both the singlet and the triplet PESs, showing distinct pathways with a triplet-to-singlet crossing occurring before the formation of a very stable singlet intermediate from which the reaction easily proceeds to yield the singlet hydroperoxo complex. The triplet pathway involves hydrogen atom abstraction by O_2_ and formation of a HOO fragment weakly bonded to Pd­(I), which rearranges into an unstable triplet hydroperoxo product.

The mechanism of the direct insertion of molecular oxygen into the gold­(III) hydride bond has not yet been fully elucidated to the best of our knowledge. The authors in ref 19 suggest that for [(^*t*Bu^PCP)­Au–H]^+^, the formation of the radical cage pair {Au·/HOO·} through hydrogen atom abstraction by O_2_ could occur in the same manner as for [(^*t*Bu^PCP)­Pd–H], but the rate of radical cage escape could be competitive with the rate of nongeminate radical recombination, which would lead to free Au· and HOO· in solution. However, a different role of the metal nature (Au/Pd) in determining the different mechanism (radical/nonradical) cannot be excluded a priori. On the other hand, the lack of reactivity of the [(CNC)­Au–H] complex with O_2_ raises the question about the fundamental role of the ligand nature (CNC/^*t*Bu^PCP).^22^ The computational study of the reaction mechanism, with reactants and products having different spin ground states, clearly involves the crossing between two diabatic triplet and singlet PESs. The commonly used approach to describe these reactions is the calculation of the so-called minimum energy crossing point (MECP) between the diabatic reactants and products PESs,^31^ which is not, however, a stationary point, and the activation energy of the process can only be estimated at MECP. Alternatively, the state-of-the-art approach is by calculations including spin–orbit coupling (SOC) effects, which allow one to locate a saddle point on a single mixed-spin adiabatic PES as well as to calculate an activation energy barrier (TS SOC).^14,15,32–34^ Although the TS SOC approach is computationally very demanding, it has the merit of allowing a detailed description of the overall reaction path along the adiabatic PES, avoiding possible pitfalls, particularly when large SOC effects are expected with heavy metals and/or when spin-crossing forms the main barrier to reaction.

In this work, we present the first DFT investigation of the insertion mechanism of O_2_ into the Au­(III)–H bond of [(^*t*Bu^PCP)­Au–H]^+^ complex which is discussed in comparison with that into the Pd­(II)–H bond of [(^*t*Bu^PCP)­Pd–H] and the Au­(III)–H bond of [(CNC)­Au–H] complexes (Scheme 1).

**Scheme 1:**
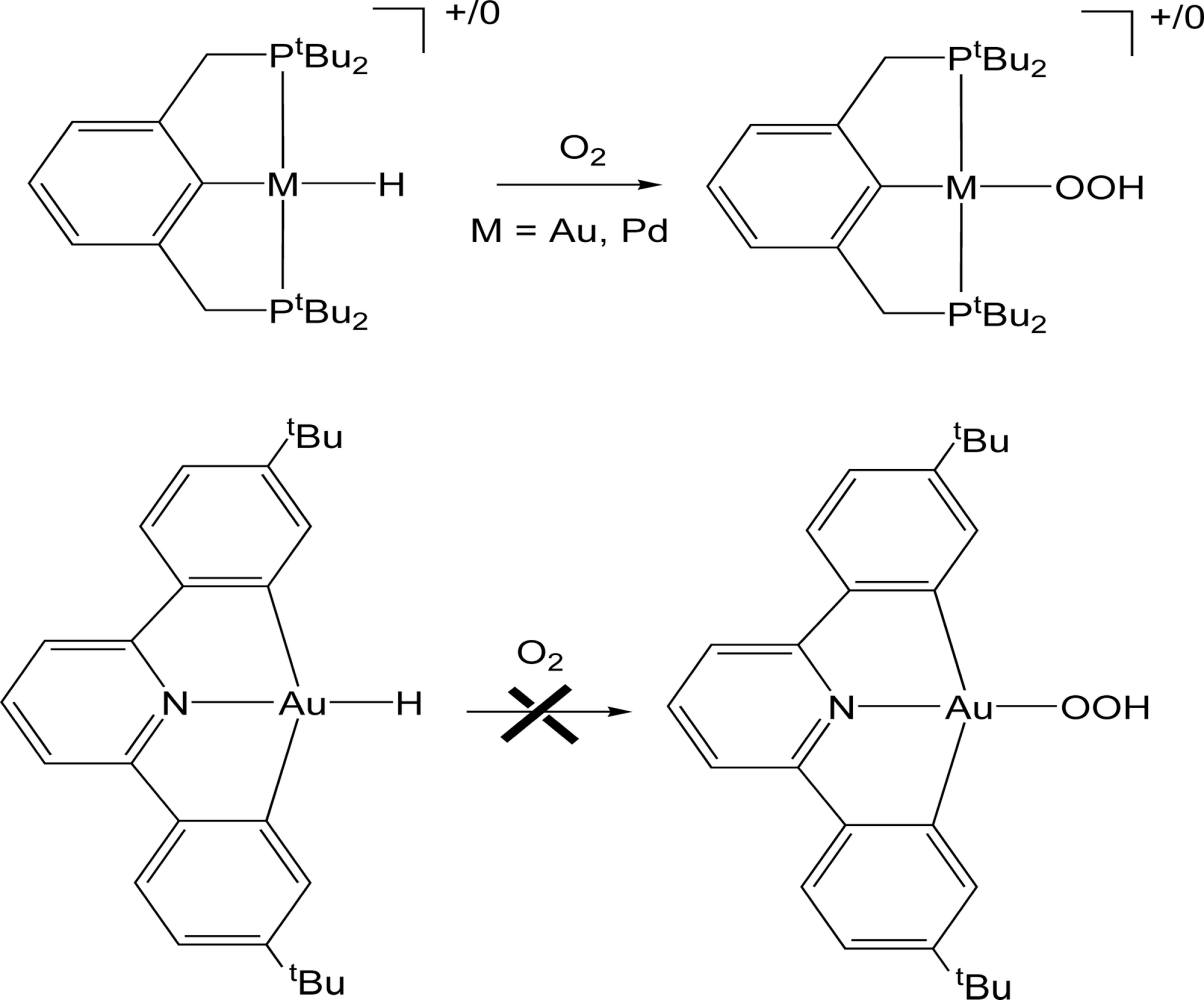
Reactions Studied in the Present Work^a^

a Insertion of molecular oxygen into M–H bond of [(^*t*Bu^PCP)­Au–H]^+^ (ref 19) and [(^*t*Bu^PCP)­Pd–H] (ref 17) to form [(^*t*Bu^PCP)­Au–OOH]^+^ and [(^*t*Bu^PCP)­Pd–OOH]. No reactivity of [(CNC)­Au–H] with O_2_ is experimentally observed.^22,23^

The TS SOC approach^35–37^ is employed to locate intermediates and transition states of the reaction on the lowest adiabatic PES. We provide evidence for a hydrogen abstraction/recombination mechanism, which diverges at the recombination step (radical vs nonradical). The M–H bond nature/reactivity relationship provides a bonding picture for the rationalization of the experimental findings. We show that the M­(δ+)–H­(δ−) polarization of the electron sharing M–H bond is likely tuning the reactivity with O_2_: the weakly (lowest) polarized Au­(III)–H bond in [(CNC)­Au–H] is connected with no reactivity, the medium (intermediate) polarized Au­(III)–H bond in [(^*t*Bu^PCP)­Au–H]^+^ with a radical-type reactivity, and the highest polarized Pd­(II)–H bond in [(^*t*Bu^PCP)­Pd–H] with a nonradical-type reactivity. This study is highly relevant as it contributes, relying on sophisticated state-of-the-art computational approaches, to the fundamental understanding of the mechanisms of aerobic oxidation of late transition metal hydrides, shedding light on the important role of both the metal and the ligand, not yet established, in this kind of gold/palladium-catalyzed oxidation reactions.

## Computational Details

The reaction energy profiles along the triplet and singlet diabatic PESs (see The O_2_ insertion mechanism—preliminary study section in the SI) and the mixed-spin adiabatic PESs were computed using the Amsterdam Density Functional (ADF) package (version 2017.01)^38^ and the companion Quantum-Regions Interconnected by Local Descriptions (QUILD)^39^ program. Specifically, geometry optimizations and frequency calculations on optimized structures (minima with zero imaginary frequencies and transition states with one imaginary frequency) were run at the BP86 ^40,41^ level, including dispersion corrections with Grimme’s D3 method together with the Becke–Johnson damping scheme (D3-BJ),^42,43^ using the Slater-type TZ2P triple-ζ quality basis set with two polarization functions for each atom, and the “core small” keyword to describe the innermost electron shells. The Conductor-Like Screening Model (COSMO) framework (solvent name = acetonitrile) was employed to account for solvation effects in both geometry optimization and frequency calculations.^44^ Among the different solvents used in the experimental studies (acetonitrile, benzene, toluene, or THF), we chose acetonitrile for calculations of all the complexes in order to compare results at the same level of theory. We expect that this choice does not have a significant effect, since all of the experimental solvents are low-polarity solvents, which should not make relevant differences within the COSMO approach.

The scalar ZORA Hamiltonian^45–47^ was adopted to calculate the reaction profiles along the diabatic triplet (reactants) and singlet (product) PESs (see SI). In the context of spin-forbidden reactions, inclusion of spin–orbit coupling SOC in the calculations (i.e., a relativistic effect that couples the electron’s spin and orbital angular momentum, particularly important for heavy elements where relativistic effects become significant) allows the reaction to be described on a single, mixed-spin (adiabatic) PES, even though the reactants and products are on different spin PESs. The spin–orbit ZORA Hamiltonian including SOC effects (SOC ZORA) was employed within the unrestricted noncollinear approximation^35–37^ to locate intermediates and transition states of the reaction on the lowest adiabatic PESs (TS SOC approach). By using the SOC ZORA Hamiltonian to approximate the relativistic Dirac equation, two-component spinors are obtained as solutions, which can be correlated to the molecular orbitals calculated with the scalar ZORA Hamiltonian.^48^ Since only the calculation of numerical frequencies at relativistic SOC level (computationally unaffordable for large systems) is supported by ADF, the ^t^Bu groups in the (^*t*Bu^PCP) ligand were substituted with methyl ones, and the free energy reaction profile at SOC level was calculated for this simplified model of [(^*t*Bu^PCP)­Au–H]^+^ ([(PCP)­Au–H]^+^). The computational setup used in this work, including functional and basis set choice, was validated in a previous benchmark study for the insertion of the O_2_ into the Au­(I)–H bond of the [(IPr)­Au­(I)­H] complex. Results are reported in the ESI of ref 14.

Energy decomposition analysis (EDA),^49^ natural orbitals for chemical valence (NOCV),^50,51^ and combined charge displacement^52^ and natural orbitals for chemical valence (CD-NOCV)^53^ approaches have been applied for the M–H (M = Au, Pd) bond analysis. A detailed description of EDA, NOCV, and CD tools can be found in the Methodology section of SI.

## Results and Discussion

### The O_2_ Insertion Mechanism

As a preliminary study, reaction free energy profiles for the hydrogen abstraction mechanism have been calculated along the diabatic open-shell singlet, singlet, and triplet PESs. Results are reported and discussed in the SI for [(^*t*Bu^PCP)­Au–H]^+^, [(^*t*Bu^PCP)­Pd–H], and [(CNC)­Au–H] (Figures S1–S13, Scheme S1). The TS SOC approach is applied here for the reactivity of the insertion of O_2_ with the three complexes. The adiabatic reaction electronic energy profiles are compared in Figure 1 for the [(^*t*Bu^PCP)­Au–H]^+^ (top left), [(^*t*Bu^PCP)­Pd–H] (top right), and [(CNC)­Au–H] (bottom) complexes. Corresponding structures of all of the stationary points along the SOC paths are shown in Figures S14–S16, and key stationary points (TSI and INT) structures are compared in Figure 2.

**Figure 1:**
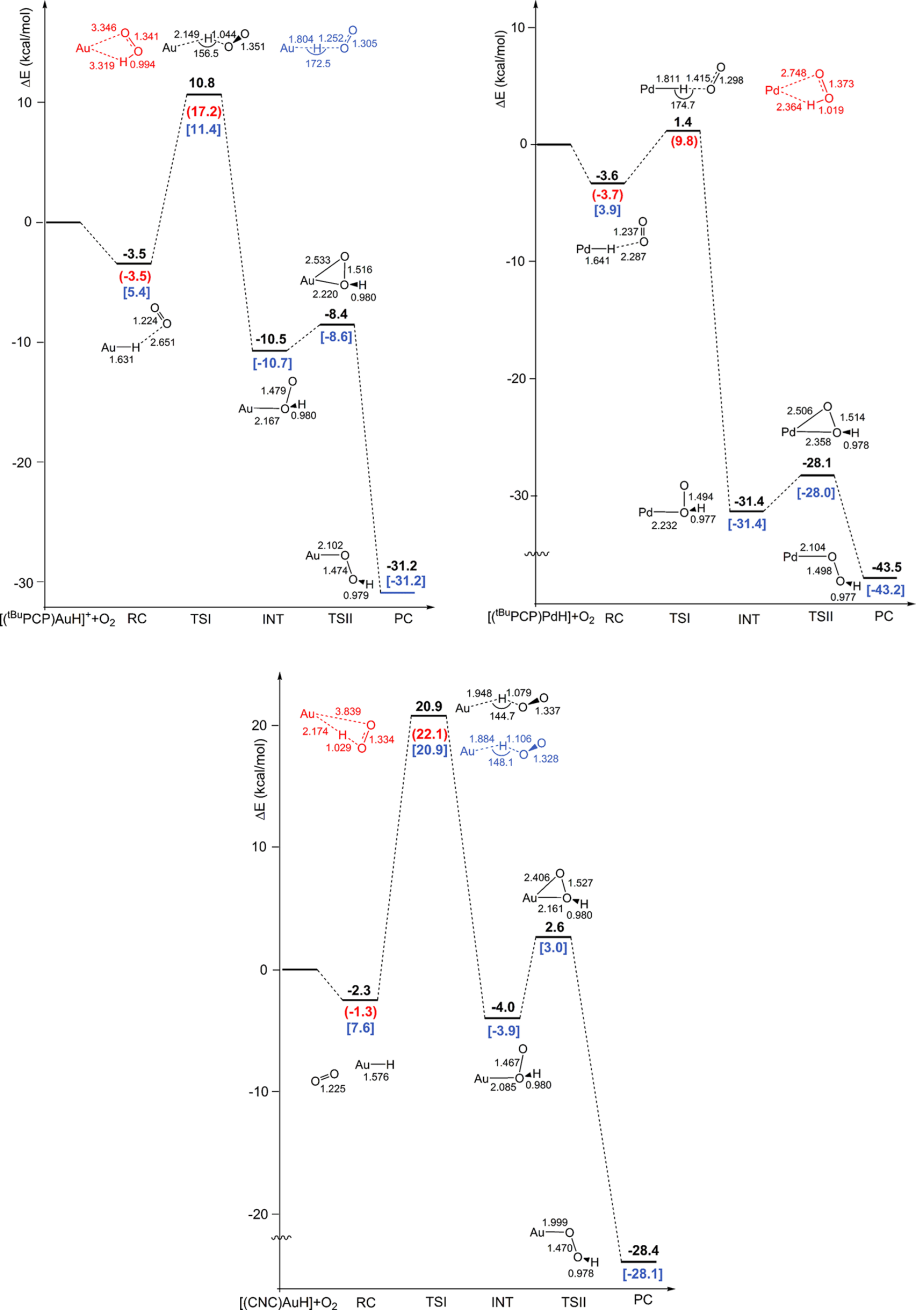
Reaction electronic energy profiles (relativistic SOC) and schematic structures (with relevant geometrical parameters in Å and degrees) for the dioxygen insertion with [(^*t*Bu^PCP)­Au–H]^+^ (top left), [(^*t*Bu^PCP)­Pd–H] (top right), and [(CNC)­Au–H] (bottom) complexes. Δ*E* SOC values (in black) are shown, and Δ*E* values for the corresponding triplet (in red) and singlet (in blue) stationary points are reported in brackets. Schematic structures (with relevant geometrical parameters in Å and degrees) of transition states calculated at the relativistic scalar level are shown for comparison in red (triplet spin state) and blue (singlet spin state). All energy values (in kcal/mol) refer to the energy of the isolated reactants taken as zero. RC = reactant complex; TS = transition state; INT = intermediate; PC = product complex.

**Figure 2:**
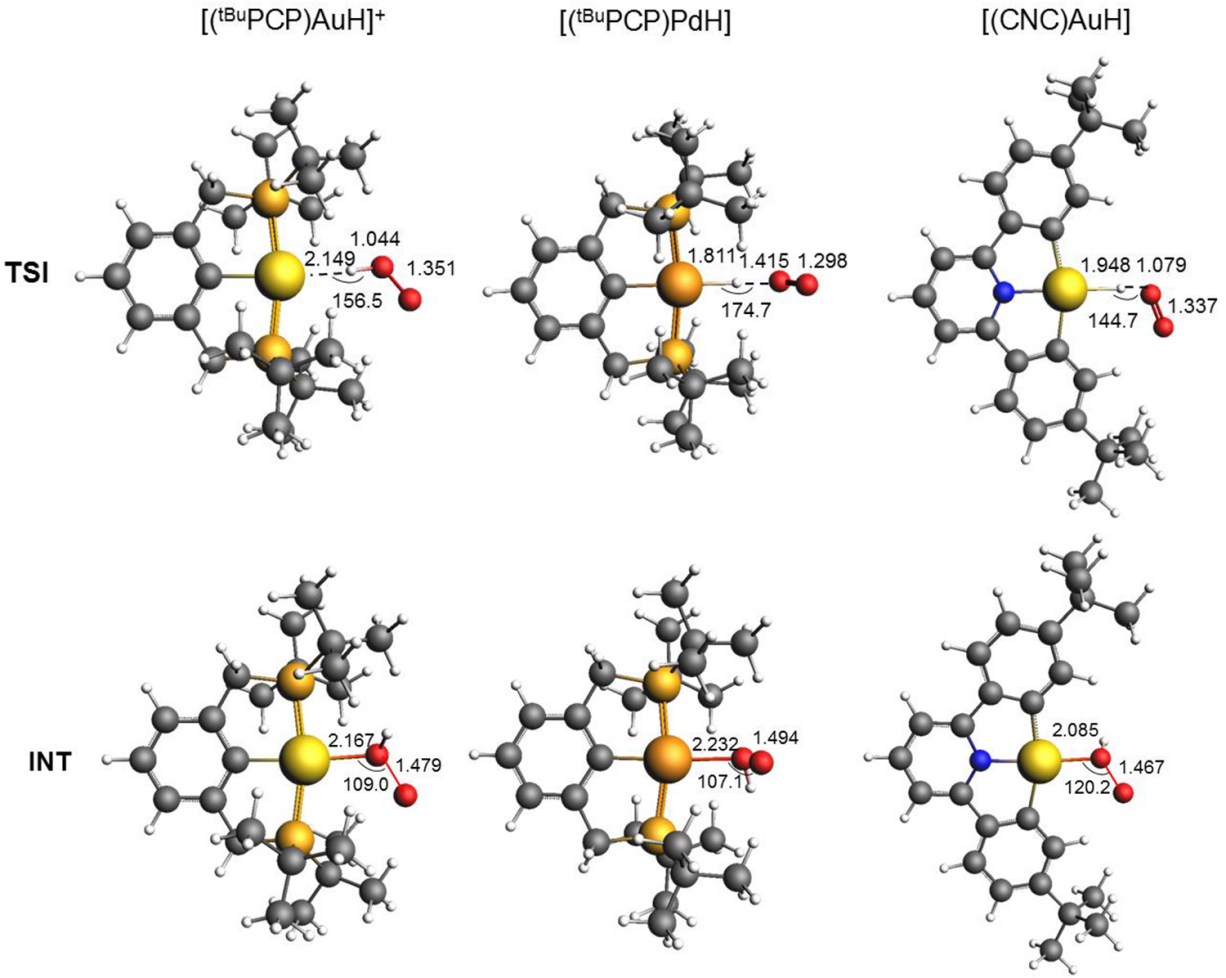
SOC structures of key stationary points (transition states, TSI and intermediates, INT) along the relativistic SOC path for the insertion of molecular oxygen into the M–H bond of the [(^*t*Bu^PCP)­AuH]^+^ (left column), [(^*t*Bu^PCP)­PdH] (central column), and [(CNC)­AuH] (right column) complexes (see Figure 1). Relevant geometrical parameters are shown (distances in Å, angles in degrees).

For [(^*t*Bu^PCP)­Au–H]^+^, the reaction along the SOC pathway initially involves the slightly exothermic (−3.5 kcal/mol) formation of a weakly bound van der Waals complex in a triplet spin state (note that RC has the same electronic energy and geometry as those calculated along the triplet path, RC^T^, see Figure S3). The first step of the reaction is the abstraction of the hydrogen atom from the gold center by O_2_ that takes place by overcoming an energy barrier of 10.8 kcal/mol via the TSI transition state, leading to a relatively stabilized intermediate (INT), which is a 1,1-O_2_ insertion product, through an exothermic step (−10.5 kcal/mol). As is evident from Figure 1 (top left), the description of the SOC PES from INT to PC, where the OOH hydroperoxo ligand rearranges to [(^*t*Bu^PCP)­Au–OOH]^+^ product formation, parallels that of the singlet PES reported in Figure S3 (both in electronic energy and geometrical structures). The reaction on the triplet PES directly gives the final 1,2-O_2_ insertion product PC^T^ in an endergonic step (Δ*G* = 10.8 kcal/mol). The TSI^T^ on the triplet PES is a concerted hydrogen abstraction/OOH rebound transition state. This is confirmed by both the imaginary frequency (95.8i cm^–1^), involving both the coordinates of the hydrogen which is abstracted and the incoming oxygen atom (see “Atomic displacements for the imaginary frequency of relevant transition states” in the SI), and by the computed two-dimensional triplet PES for the H abstraction/OOH rebound steps for [(^*t*Bu^PCP)­Au–H]^+^, using the Au–O1 and O2–H bond distances as reaction coordinates (see Figure S2).

All attempts to individuate a singlet intermediate with a structure analogous to that of the triplet final 1,2-O_2_ insertion product complex were unsuccessful, despite the numerous strategies used to find it. Analogous results have been reported in ref 29, where the insertion mechanism of molecular oxygen into a Pd­(II)–H bond has been computationally studied using the MECP method, showing that along the triplet pathway the reaction evolves through the abstraction of the hydrogen atom by O_2_ and formation of the final hydroperoxo product, while the reaction mechanism along the singlet PES involves, as a result of the hydrogen atom abstraction, the formation of a very stable intermediate, [(PCP)­Pd­(OHO)] (1,1-O_2_ insertion product). The authors report that, due to the stabilization of this intermediate, the crossing between triplet and singlet occurs exactly in this region of the PESs. To confirm that the computed TSI SOC in Figure 1 is a transition state that leads directly to intermediate INT, the SOC PES has been explored for the [(^*t*Bu^PCP)­Au–H]^+^ complex reactivity with O_2_, which allows us to get an idea about the reaction landscape. Results are reported and discussed in the SI (Figures S18–S23), mainly suggesting that formation of the [(^*t*Bu^PCP)­Au·]^+^ radical after hydrogen abstraction can be only stabilized through interaction with the ·OOH radical, indicating that the hydrogen abstraction should occur concerted (or at least barrierless) with the OOH rebound which takes place through the 1,1-O_2_ insertion product (INT) formation.

For [(^*t*Bu^PCP)­Pd–H] (Figure 1, top right), the results analogously show that along the SOC pathway, the reaction evolves through the abstraction of the hydrogen atom by O_2_ (TSI) and formation of a HOO fragment bonded to the Pd center (INT). From the highly stabilized intermediate INT, exothermic formation of the final product (PC) takes place, overcoming the low barrier associated with the transition state (TSII) for the simultaneous breaking of the bond with proximal oxygen and bond forming with distal oxygen atom. Similar to [(^*t*Bu^PCP)­Au–H]^+^, the description of the SOC PES from INT to PC parallels that of the singlet PES reported in Figure S7 (both in electronic energy and geometrical structures). Calculation of TSI highlights the importance of the SOC effects in lowering the energy barrier of the hydrogen abstraction step by 8.4 kcal/mol with respect to the triplet transition state, indicating very low electronic energy barriers for both the hydrogen abstraction and the rearrangement steps (Δ*E* ^⧧^ = 1.4 and 3.3 kcal/mol, respectively). Remarkably, in the TSI structure, the O_2_ orientation is perpendicular to the complex plane with an almost linear Pd–H–O configuration (174.7°) (Figure 2). It is worthwhile to underline that the mechanism calculated along the SOC PES allows us to avoid any uncertainty concerning the relative position of the triplet-singlet spin crossing point (MECP) and the first transition state. For instance, the localization and characterization of the MECP for this reaction have been under discussion in previous theoretical studies.^29^ Although inclusion of SOC in a noncollinear approximation as used in this work for investigating the spin-forbidden late transition metal–hydride complex reactivity with O_2_ is not a routine mechanistic approach, it has the merit that, enabling the reaction to occur on a single adiabatic PES, a transition state (TS SOC), as well as an activation energy, can be calculated. In contrast, the commonly used MECP approach only allows for an estimate of the energy barrier and a detailed description of the overall reaction path, which avoids possible pitfalls when tricky diabatic PESs are involved, is exclusively possible through the TS SOC approach.

For [(CNC)­Au–H] (Figure 1, bottom), the activation electronic energy barrier on the SOC PES is very high (Δ*E* ^⧧^ = 20.9 kcal/mol and Figure S12), consistent with the lack of reactivity with O_2_ experimentally reported for this complex. Very interestingly, the TS SOC structure is very similar to that calculated for [(^*t*Bu^PCP)­Au–H]^+^, and they are both different from the TSI structure calculated for [(^*t*Bu^PCP)­Pd–H] (Figure 2).

To get an estimate of the triplet/singlet spin state contribution to the TS SOC for the three complexes, it is worthwhile to calculate single-point energies at the TS SOC-optimized geometries. Results are reported in Table S1. Spin density distribution is also presented for the triplet spin single points in Table 1.

**Table 1:** Electron α Spin Density Distribution (Mulliken, in e) Calculated from the Triplet Spin Single Point at the Corresponding TS SOC Geometry for the [(^*t*Bu^PCP)­Au–H]^+^, [(^*t*Bu^PCP)­Pd–H], and [(CNC)­Au–H] Complexes (Figure 1) Columns^a^

	C/N	M	H	O1	O2
[(^*t*Bu^PCP)Au–H]^+^	0.40	0.29	0.05	0.40	0.71
[(^*t*Bu^PCP)Pd–H]	0.19	0.18	0.10	0.70	0.74
[(CNC)Au–H]	0.22	0.40	0.05	0.48	0.73

a C/N indicates the ligand atom bonded to the metal in trans position with respect to H; M = Au, Pd; and O1 refers to the oxygen atom of O_2_ forming a bond with hydrogen.

From Table S1, the triplet state contribution at the TS SOC geometry is slightly larger than the singlet for all the [(^*t*Bu^PCP)­Au–H]^+^ (−3.1 kcal/mol), [(^*t*Bu^PCP)­Pd–H] (−2.7 kcal/mol), and [(CNC)­Au–H] (−0.8 kcal/mol) complexes. Moreover, while singlet and open shell singlet energies are identical for [(^*t*Bu^PCP)­Pd–H] and [(CNC)­Au–H], the open shell singlet state energy is lower than the singlet one for [(^*t*Bu^PCP)­Au–H]^+^. However, for this complex, the open-shell singlet is not a pure spin state, suffering from spin contamination (⟨*S* ^2^⟩ = 0.74), meaning that mixing with the triplet state is responsible for a 4.4 kcal/mol lowering of the singlet state energy. Comparison between electron spin distribution calculated from the triplet spin single point at the corresponding TS SOC geometries (Table 1) shows that (i) for [(^*t*Bu^PCP)­Pd–H], the two unpaired electrons are mainly localized on each of the two oxygen atoms of O_2_; (ii) for [(CNC)­Au–H] and [(^*t*Bu^PCP)­Au–H]^+^, one unpaired electron is roughly localized on one oxygen atom of O_2_ (that not abstracting hydrogen) and one unpaired electron is mainly delocalized between the second oxygen atom of O_2_ interacting with H, the Au center and the N/C ligand atom bonded to Au in trans position to H, although to a different extent. Indeed, while one unpaired electron is mainly delocalized over the oxygen atom interacting with H and the Au centers for [(CNC)­Au–H], for [(^*t*Bu^PCP)­Au–H]^+^ it is more extensively distributed over the oxygen atom interacting with H, the Au and the C ligand atom bonded to Au in the trans position to H centers, revealing a relevant role of the ligand in delocalizing the unpaired electron. In Figure 3, the TSI SOC structure for the [(^*t*Bu^PCP)­Au–H]^+^ and the two singly occupied α spin MO (102 and 106 aα) resulting from the triplet spin state single-point calculation on this geometry are depicted, which clearly shows the radical pair formation at the transition state. The 102 aα describes a bonding MO between H and O_2_ (which localizes one α electron on the OOH group), while 106 aα represents an antibonding MO between O_2_ and the gold fragment, mainly involving Au and C atom ligand, with a much smaller contribution from O_2_ (which localizes one α electron on the gold fragment).

**Figure 3:**
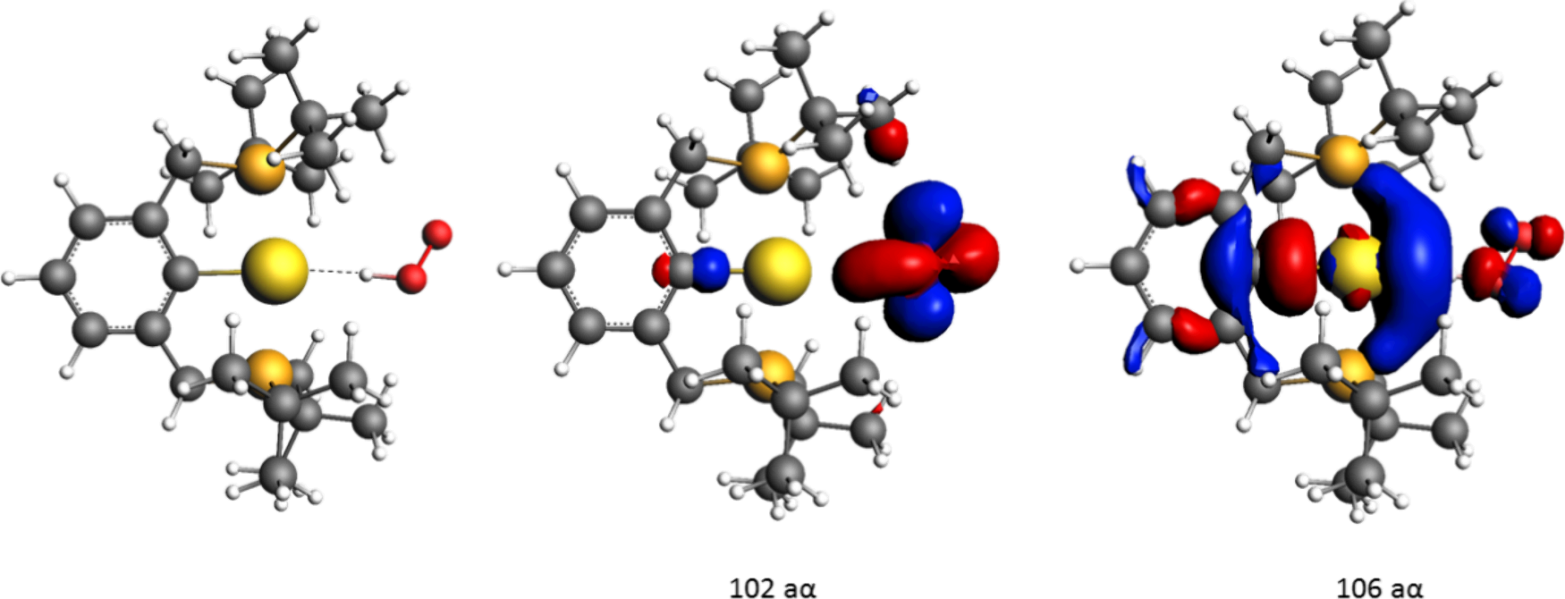
: [(^*t*Bu^PCP)­Au–H]^+^ TSI SOC structure (left) and the two singly occupied α spin MO (102 and 106 aα) resulting from the triplet spin state single-point calculation on the [(^*t*Bu^PCP)­Au–H]^+^ TSI SOC geometry.

Results in Tables S1 and 1, all together, suggest that at the [(^*t*Bu^PCP)­Pd–H] TS SOC no radical pairs are involved (the TS SOC has mainly a triplet character with the two unpaired electrons on O_2_ which can be described as a polarized biradicaloid species), whereas at the [(CNC)­Au–H] and [(^*t*Bu^PCP)­Au–H]^+^ TS SOCs radical pairs are formed (the TS SOC has mainly a triplet character with one unpaired electron on O_2_ and one on the gold fragment), with a larger radical pair stabilization due to the delocalization ability at the TS SOC of the (^*t*Bu^PCP) ligand. The above results suggest a heterolytic cleavage of the Pd–H bond with H^–^ hydride transfer to O_2_, with no radical pair involvement. On the contrary, a homolytic breaking of the Au–H bond can be expected, with H· atom transfer to molecular oxygen. Very interestingly, the [(^*t*Bu^PCP)­Au–H]^+^ reactivity with O_2_ is strongly reminiscent of the “radical-like” (or “hidden-radical”) reactivity previously observed by us for gold aluminyl and gold hydride complexes with other small molecules.^54–59^

To make the SOC numerical frequency calculations affordable, the ^t^Bu groups in the ^*t*Bu^PCP ligand of [(^*t*Bu^PCP)­Au–H]^+^ were substituted with methyl ones. The same simplification was applied in ref 29 for [(^*t*Bu^PCP)­Pd–H], where testing calculations showed that no significant structural change was introduced. Analogous, we find that the simplified complex [(PCP)­Au–H]^+^ is a very reasonable model for the real [(^*t*Bu^PCP)­Au–H]^+^ one, as demonstrated by the adiabatic free energy reaction profile shown in Figure 4 (to be compared with that shown in Figure 1, top left) and by the TSI SOC structure comparison in Figure S17.

**Figure 4:**
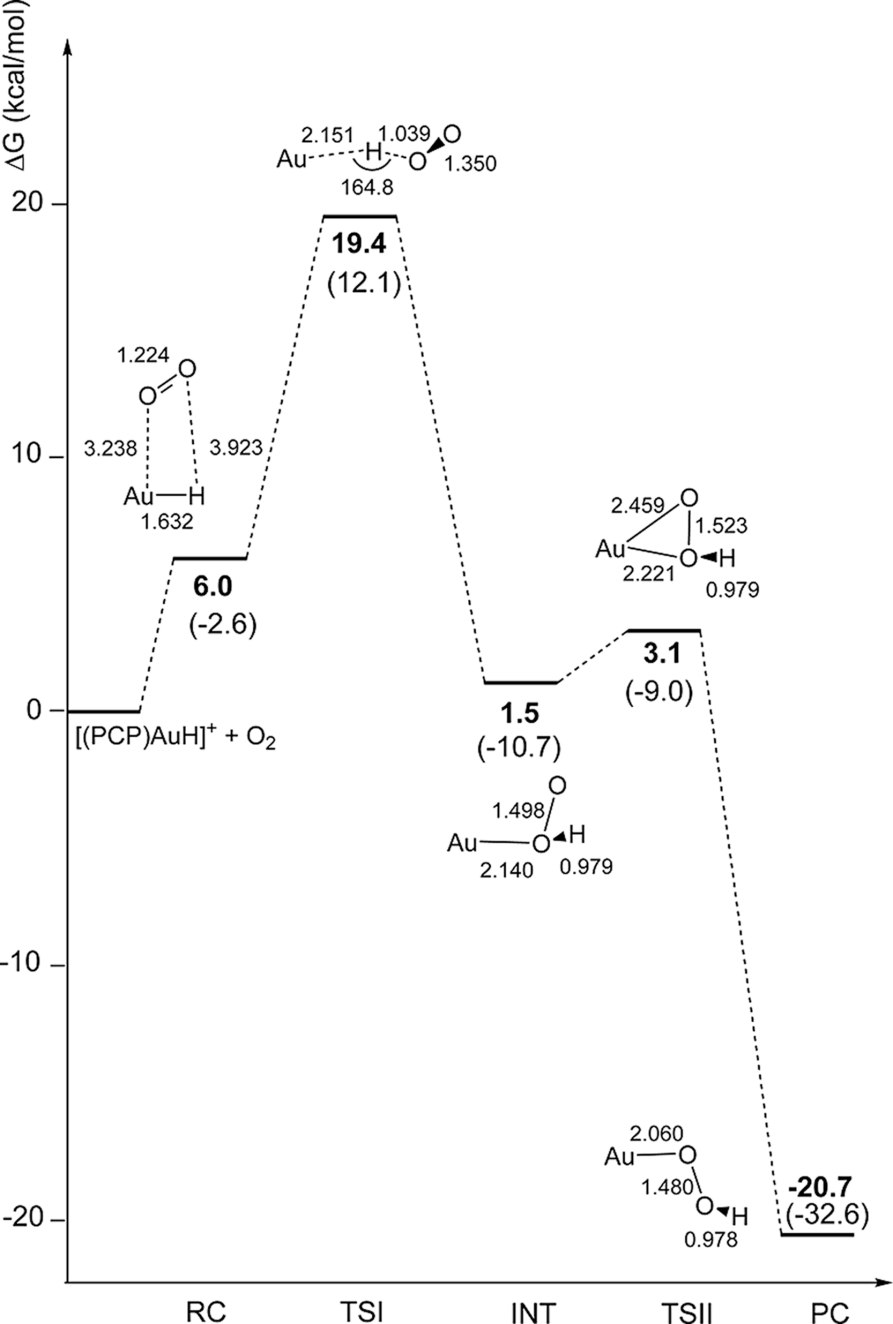
Reaction free energy profile (SOC) and schematic structures (with relevant geometrical parameters in Å and degrees) for the hydrogen abstraction mechanism with the model [(PCP)­Au–H]^+^ complex. Δ*G* values (in kcal/mol) refer to the energy of the isolated reactants in their ground spin state taken as zero. Values in parentheses are the corresponding Δ*E* values (in kcal/mol).

Along the path, both electronic energies and geometries of intermediates and transition states are very similar for model [(PCP)­Au–H]^+^ and real [(^*t*Bu^PCP)­Au–H]^+^ complexes. In particular, the TSI SOC has been verified to be a transition state structure, with only one negative frequency (703.6i cm^–1^) as well as the TSII SOC (188.7i cm^–1^). While usually TS frequencies for hydrogen abstraction processes are found to be above 1000i cm^–1^, lower frequencies are not uncommon and have been reported in several instances.^60–62^ In conclusion, these results indicate a “radical-like” reactivity of [(^*t*Bu^PCP)­Au–H]^+^ with O_2_ in a kinetically feasible hydrogen abstraction/rearrangement mechanism, where the hydrogen abstraction is the rate-determining step (Δ*G* ^⧧^ = 19.4 kcal/mol). SOC numerical frequency calculations have been similarly performed for the reaction with [(^*t*Bu^PCP)–Pd–H] and [(CNC)­Au–H] using simplified models, [(PCP)–Pd–H] and [(CNC′)­Au–H] (where CNC′ represents the CNC ligand where the two ^t^Bu groups are substituted by two methyl groups). The TSI SOC shows only one negative frequency for both [(PCP)–Pd–H] and [(CNC′)­Au–H] (162.3i and 494.1i cm^–1^, respectively). A more complete description of the TSI SOC vibrational modes is given in the SI (see section “Atomic displacements for the imaginary frequency of relevant transition states”), which shows that atomic displacements for the imaginary frequency in all complexes involve the coordinates of the hydrogen atom that is abstracted. For [(PCP)–Pd–H], they involve, in addition to that, the coordinates of the two oxygen atoms of the O_2_ moiety.

Corresponding free energy activation barriers Δ*G* ^⧧^ have been calculated, amounting to 14.1 and 27.8 kcal/mol for [(PCP)–Pd–H] and [(CNC′)­Au–H], respectively, fully consistent with experiment.

### Rationalization of the Experiment: Dissociation of Radical Pairs for [(^*t*Bu^PCP)­Au–H]^+^

In ref 19, a dissociation of [(^*t*Bu^PCP)­Au·]^+^ and ·OOH pair and generation of a second radical chain carrier through reaction of ·OOH with [(^*t*Bu^PCP)­AuH]^+^ were proposed. However, from experimental kinetic results, a definitive mechanism for this transformation remains elusive. Very interestingly, our computational study suggests that the resulting [(^*t*Bu^PCP)­Au·]^+^ radical is stabilized both by the unpaired electron delocalization over the ligand and by the ·OOH trapping at the INT structure (Figure 1 top left and Figure 4). The INT structure represents the experimentally defined “radical cage” [[(^*t*Bu^PCP)­Au·]^+^ + ·OOH], where radicals are generated close together and they can react before diffusing apart (“geminated recombination”); the in-cage PC formation has a very low activation free energy barrier (Δ*G* ^⧧^ = 1.6 kcal/mol, Figure 4). However, the INT formation is slightly endergonic (Δ*G* = 1.5 kcal/mol, Figure 4) and one may wonder if the ·OOH could easily be dissociated (“radical cage escape”), initiating a radical chain and if the [(^*t*Bu^PCP)­Au·]^+^ radical could be trapped by the diradical O_2_ substrate itself during initiation or propagation. The following reactions were proposed in ref 19, for which the free energy Δ*G* values are here calculated using the real [(^*t*Bu^PCP)­Au–H]^+^ complex.

Initiation:

$$[{(}^{t\mathrm{Bu}}\mathrm{PCP})\mathrm{Au-H}{]}^{+}+O_{2}\to [{(}^{t\mathrm{Bu}}\mathrm{PCP})\mathrm{Au\cdot}{]}^{+}+\mathrm{\cdot OOH},\Delta G=+18.0\;\mathrm{kcal}/\mathrm{mol}$$ 1

$$\mathrm{\cdot OOH}+[{(}^{t\mathrm{Bu}}\mathrm{PCP})\mathrm{Au-H}{]}^{+}\to [{(}^{t\mathrm{Bu}}\mathrm{PCP})\mathrm{Au\cdot}{]}^{+}+H_{2}O_{2},\Delta G=-11.2\;\mathrm{kcal}/\mathrm{mol}$$ 2

Propagation:

$$[{(}^{t\mathrm{Bu}}\mathrm{PCP})\mathrm{Au\cdot}{]}^{+}+O_{2}\to [{(}^{t\mathrm{Bu}}\mathrm{PCP})\mathrm{Au}O_{2}\cdot {]}^{+},\Delta G=-18.6\;\mathrm{kcal}/\mathrm{mol}$$ 3

$$[{(}^{t\mathrm{Bu}}\mathrm{PCP})\mathrm{Au}O_{2}\cdot {]}^{+}+[{(}^{t\mathrm{Bu}}\mathrm{PCP})\mathrm{Au-H}{]}^{+}\to [{(}^{t\mathrm{Bu}}\mathrm{PCP})\mathrm{Au-OOH}{]}^{+}+[{(}^{t\mathrm{Bu}}\mathrm{PCP})\mathrm{Au\cdot}{]}^{+},\Delta G=+0.3\;\mathrm{kcal}/\mathrm{mol}$$ 4

Autoacceleration:

$$H_{2}O_{2}+[{(}^{t\mathrm{Bu}}\mathrm{PCP})\mathrm{Au-H}{]}^{+}\to [{(}^{t\mathrm{Bu}}\mathrm{PCP})\mathrm{Au\cdot}{]}^{+}+H_{2}O+\mathrm{\cdot OH},\Delta G=-1.9\;\mathrm{kcal}/\mathrm{mol}$$ 5

$$\mathrm{\cdot OH}+[{(}^{t\mathrm{Bu}}\mathrm{PCP})\mathrm{Au-H}{]}^{+}\to [{(}^{t\mathrm{Bu}}\mathrm{PCP})\mathrm{Au\cdot}{]}^{+}+H_{2}O,\Delta G=-48.3\;\mathrm{kcal}/\mathrm{mol}$$ 6

All the species in reactions 1–6 have been optimized at their ground spin states, i.e., singlet for [(^*t*Bu^PCP)­Au–H]^+^, [(^*t*Bu^PCP)­Au–OOH]^+^, H_2_O_2_, and H_2_O, doublet for [(^*t*Bu^PCP)­Au·]^+^, [(^*t*Bu^PCP)­AuO_2_·]^+^, ·OOH, and ·OH, and triplet for O_2_. The equilibrium of the stoichiometric reaction 1 significantly favor the reactant side (Δ*G* = +18.0 kcal/mol) and so it would not proceed without excess of substrate to trap the [(^*t*Bu^PCP)­Au·]^+^ radical species, which is fully consistent with the kinetic experiments performed by using elevated pressures of O_2_, 5–10 atm, with O_2_ always in excess.^19^ Indeed, initiation reaction 1 would be thermodynamically favored by excess dioxygen, as shown in reaction 7:

$$[{(}^{t\mathrm{Bu}}\mathrm{PCP})\mathrm{Au-H}{]}^{+}+2O_{2}\to [{(}^{t\mathrm{Bu}}\mathrm{PCP})\mathrm{Au}O_{2}\cdot {]}^{+}+\mathrm{\cdot OOH},\Delta G=-0.6\;\mathrm{kcal}/\mathrm{mol}$$ 7

Notably, Δ*G* for reactions 1 and 3, which involve formation (from Au–OOH homolitic bond breaking) and recombination (with Au-OO homolitic bond formation) of the [(^*t*Bu^PCP)­Au·]^+^ radical species, are practically identical in absolute value (+18.0 and −18.6 kcal/mol), consistent with the radical mechanism picture and suggesting that [(^*t*Bu^PCP)­Au·]^+^ would be equally efficiently stabilized by both ·OOH and O_2_ radicals.

For comparison, the free energy Δ*G* values for stoichiometric reactions 1 and 3 have been calculated with the [(CNC)­AuH] complex, which shows no reactivity with O_2_, finding for reaction 1 Δ*G* = +27.6 kcal/mol and for reaction 3 Δ*G* = −22.3 kcal/mol. Both values clearly suggest that the [(CNC)­Au·] radical is less stable than its [(^*t*Bu^PCP)­Au·]^+^ counterpart, likely due to the lower ability of the (CNC) ligand to delocalize the unpaired electron. Spin delocalization for [(^*t*Bu^PCP)­Au·]^+^ and [(CNC)­Au·] radicals is compared in Figure S24. Interestingly, use of dioxygen excess would not yield the desired radical stabilization, as revealed by the Δ*G* value calculated for the corresponding reaction 7, which remains endergonic (+5.3 kcal/mol). For [(^*t*Bu^PCP)­PdH], reactivity with O_2_ has been suggested not to be through a radical mechanism. Δ*G* calculations for corresponding reactions 1 and 3 give +17.0 and −34.4 kcal/mol, respectively. The much larger absolute Δ*G* value for reaction 3 with respect to that for reaction 1 is consistent with the picture that formation of a [(^*t*Bu^PCP)­Pd·] radical is unlikely (see Figure S24 for spin delocalization) and that an acidic [(^*t*Bu^PCP)­Pd]^+^ species would be rather involved in the mechanism. Interestingly, a second-order rate law (first-order in palladium and first-order in oxygen) was experimentally documented as well as evidence of significant Pd–H bond cleavage in the rate-determining step,^17^ consistent with a hydrogen abstraction mechanism not requiring an excess of dioxygen.

Based on the above results, a plausible scenario for the radical chain mechanism with [(^*t*Bu^PCP)­Au–H]^+^ would be ·OOH dissociation from intermediate INT occurring through an exchange process, where ·OOH is replaced by an O_2_ diradical. The reaction free energy profile (relativistic SOC) using the model [(PCP)­Au–H]^+^ complex is shown in Figure 5. The ·OOH replacement by O_2_ at the INT structure occurs through an interchange associative mechanism (Figure 5), along a pure spin triplet PES. Optimization of the stationary points along the triplet PES gives structures practically identical to those calculated at the SOC level. Starting from RC_O_2__, where two unpaired electrons are mainly localized on the O_2_ (0.71e and +0.90e α spin densities), at the transition state TS_sub_ (ν = 108.7i cm^–1^) they are delocalized over both the O_2_ moiety (0.72e + 0.68e α spin densities) and the OOH one (0.18e + 0.45e α spin densities) and, at the PC_rad_ structure, one unpaired electron resides on the OO moiety coordinated to gold (0.37e + 0.63e α spin densities on the O atom directly bonded to Au and on the external O atom, respectively) and one unpaired electron lies on the OOH fragment (0.36e + 0.66e α spin densities on the O atom bonded to H and on the “free” O atom, respectively) (Figure 5), demonstrating formation of two ·OOH and [(^*t*Bu^PCP)­AuO_2_·]^+^ radicals. The activation barrier calculated at TS_sub_ (Δ*G* ^⧧^ = 9.9 kcal/mol) suggests that OOH/O_2_ exchange is indeed also kinetically feasible, in a slightly exergonic equilibrium between the [(^*t*Bu^PCP)­Au–OHO–O_2_]^+^ (RC_O_2__) species and the diradical product [(^*t*Bu^PCP)­AuO_2_· ·OOH]^+^ (PC_rad_). Similar ·OOH replacement by O_2_ at the INT structure occurring through an interchange associative mechanism, along a pure spin triplet PES, has been calculated with the model [(PCP)­Pd–H] complex, and the reaction energy profile is shown in Figure S25 for comparison. At the PC_rad_ structure, although roughly one unpaired electron resides on the OO moiety coordinated to palladium, one unpaired electron is delocalized over both Pd and the OOH fragment, demonstrating that the formation of two ·OOH and [(^*t*Bu^PCP)­PdO_2_·] radicals is not likely to occur.

**Figure 5:**
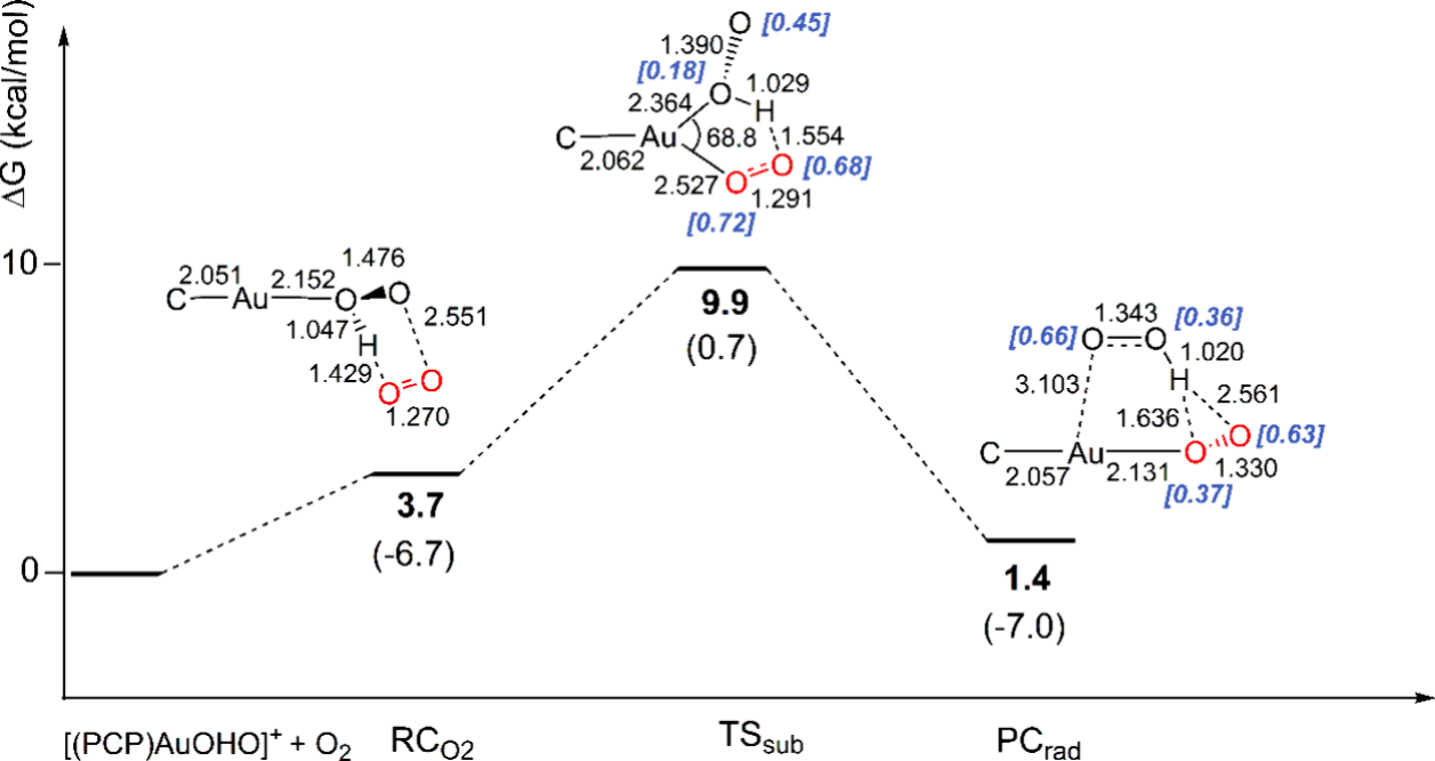
Reaction free energy profile (SOC) and schematic structures (with relevant geometrical parameters in Å and degrees) for the mechanism of the substitution of OOH by O_2_ from the [(PCP)­Au–OHO]^+^ INT structure with the model [(PCP)­Au–H]^+^ complex. Δ*G* values (in kcal/mol) refer to the energy of the isolated reactants ([(PCP)­Au–OHO]^+^ + O_2_) in their ground spin state taken as zero. Values in parentheses are the corresponding Δ*E* values (in kcal/mol). Values in square brackets (blue color) indicate unpaired α spin densities (Mulliken, in e).

Remarkably, for the [(IPr)­Au­(I)–H] reactivity with O_2_, we computationally demonstrated that a mechanism involving two O_2_ molecules was also feasible, showing combined features of the hydrogen abstraction and dioxygen metal coordination mechanisms (O_2_ oxidative addition Au­(I)/Au­(III)/recombination).^14,15^ At variance, for [(^*t*Bu^PCP)­Au­(III)–H]^+^, where the metal is already in its higher oxidized state, the second O_2_ molecule is likely involved only after the hydrogen abstraction step, fully consistent with the picture that the [(^*t*Bu^PCP)­Au·]^+^ radical stabilization, which favors the radical reactivity, could be exploited by an excess of O_2_.

In conclusion, insertion of O_2_ into the Au­(III)–H bond has been found to occur through a two-step hydrogen abstraction/recombination mechanism. At the recombination step, however, two channels are accessible: along the singlet PES, the Au­(III)–OOH product is formed in a radical-like process (“geminate recombination”); along the triplet PES, the [(^*t*Bu^PCP)­AuO_2_·]^+^ and ·OOH radicals are generated (“radical cage escape”), initiating radical chain pathways autoaccelerated by the hydrogen peroxide formed in the initiation, consistent with the experimental kinetic results.^19^ Our findings indicate that the radical chain mechanism is driven by excess of O_2_.

### The M–H (M = Au, Pd) Bond Nature

To elucidate the different reactivity with O_2_, we have performed a M–H (M = Au, Pd) bond analysis aimed at highlighting major differences in the three metal–hydride complexes under study, using a combination of approaches, including the energy decomposition analysis (EDA), charge displacement (CD) analysis, and the natural orbitals for chemical valence (NOCV) method. We previously demonstrated that application of these tools provides an unbiased computational protocol particularly suited for the description of chemical bonds involved in several catalytic processes, including small molecule activation processes.^54,63–65^

Following this procedure, we carry out a comparative EDA to understand the most suitable fragmentation to analyze the nature of the metal–hydride bond in the complexes. In all cases, as shown in Table S2, we find that the fragmentation scheme involving open-shell metal and hydride fragments has an associated less negative orbital interaction energy (Δ*E*_oi_), corresponding to the fragments with an electronic structure that more closely resembles that they acquire upon bond formation. As previously discussed,^63,66^ this result indicates that the open-shell fragmentation scheme is the most suitable to describe the M–H bond in the three complexes, suggesting an electron-sharing bond type. To gather a quantitative perspective on the M–H bond nature and polarization, we carry out a combined CD-NOCV analysis of the Pd–H and Au–H bonds in [(^*t*Bu^PCP)­Pd–H], [(^*t*Bu^PCP)­Au–H]^+^ and [(CNC)­Au–H], respectively (Figure 6).

**Figure 6:**
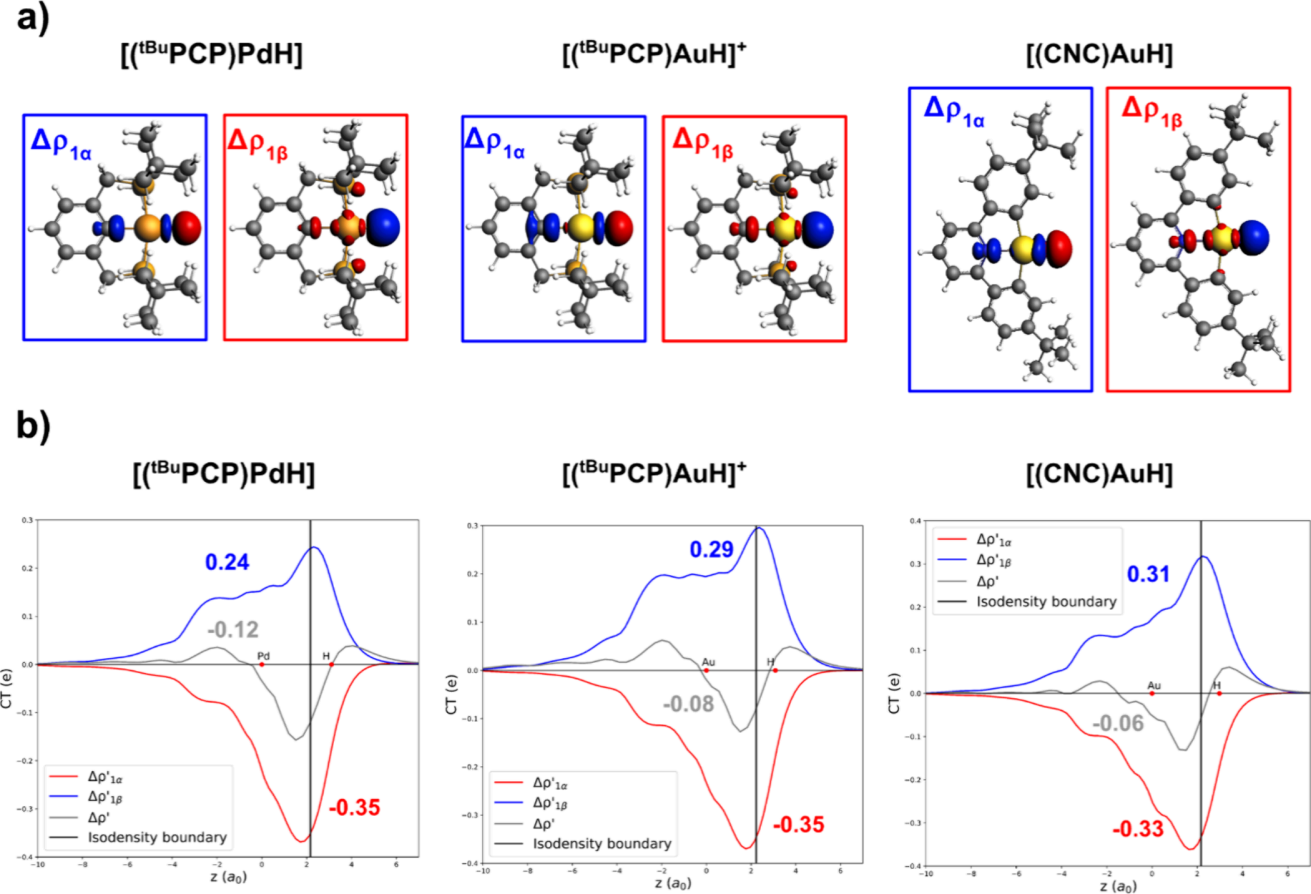
Natural orbitals for chemical valence (NOCV) deformation densities (a) and charge displacement (CD)–NOCV curves associated with the Δρ′_1α_ and Δρ′_1β_ components and the total Δρ′ (b) for the interaction between open-shell doublet [M]^0/+^ (M = [(^*t*Bu^PCP)­Pd]], (^*t*Bu^PCP)­Au]^+^, [(CNC)­Au)]) and [H] fragments in [MH]^0/+^ complexes. Red dots indicate the average positions of the nuclei along the *z*-axis. The metal (Pd in the left panel, Au in the center and right panels) fragment is placed at the left of the isodensity boundary (black vertical line), and the hydrogen fragment is placed at the right. Positive (negative) values of the curve indicate a right-to-left (left-to-right) charge transfer. The CT values calculated at the isodensity boundary are reported for each curve. See the Methodology section for details of the bond analysis.

As shown in Figure 6a, the NOCV analysis reveals that the natures of the Pd–H and Au–H bonds in the three complexes are qualitatively analogous. For all the complexes, the metal–hydride bonds can be described in terms of two main charge transfer components between the metal and ligand fragments: (i) Δρ′_1α_, i.e., H-to-M charge transfer, and (ii) Δρ′_1β_, M-to-H charge transfer. Inspection of the CD-NOCV curves (Figure 6b) displays quantitative differences between the three M–H bonds in terms of bond polarization. Specifically, the net charge transfer (CT_net_, gray curves in Figure 6b) between the metal and hydrogen fragments is always slightly negative, indicating electron sharing bonds with a minor degree of M­(δ+)–H­(δ−) polarization in the three complexes, but to different extents. We find CT_net_ values of −0.12*e*, −0.08*e*, and −0.06*e* for [(^*t*Bu^PCP)­Pd–H], [(^*t*Bu^PCP)­Au–H]^+^, and [(CNC)­Au–H], respectively, suggesting a decreasing M­(δ+)–H­(δ−) polarization in this order. Although differences in bond polarization seem to be small, they are able to induce changes in reactivity that are appreciable from an experimental perspective, as also reported in other cases (for instance, in carbon dioxide reactivity with Au–Al and Au–B complexes^54,59^ or in carbon dioxide, dihydrogen, and alkyne reactivity with coinage metal–aluminyl bonds^54,56,67^ ). Indeed, the bond polarization values that we calculate inherently reflect how the ligand (and the metal) delocalizes the electron density (Figure 6).

The NOCV approach can be coupled with the EDA within the ETS-NOCV framework,^51^ allowing the EDA orbital interaction term Δ*E*_oi_ to be further decomposed into NOCV pairwise orbital terms which associate an energy contribution (Δ*E* ^k^_oi_) to each NOCV deformation density (Δρ′_k_) (see Methodology in the SI). We find that, although similar energy values are associated with Δρ′_1α_ (−49.0/-49.7 kcal/mol), energy values associated with Δρ′_1β_ are significantly different, −17.4, −26.7 and −30.2 kcal/mol for [(^*t*Bu^PCP)­Pd–H], [(^*t*Bu^PCP)­Au–H]^+^ and [(CNC)­Au–H], respectively, quantifying the two opposite charge fluxes resulting in a bond polarization which decreases in the above order (see Table S3).

To rationalize this bond polarity trend, we analyze the nature and orbital composition of the highest-lying singly occupied molecular orbital (SOMO) of the open-shell [(^*t*Bu^PCP)­Pd], [(^*t*Bu^PCP)­Au]^+^, and [(CNC)­Au] fragments, which is directly involved in both Δρ_1α_′ and Δρ_1β_′ charge fluxes. As shown in Figure 7, the three SOMOs, while qualitatively analogous, display quantitatively different composition.

**Figure 7:**
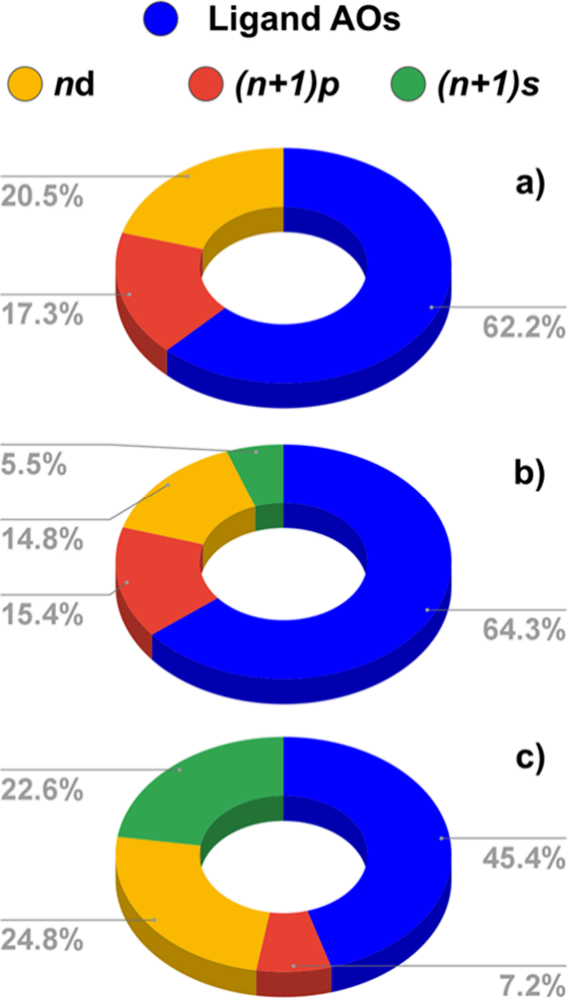
Character of the highest-lying singly occupied molecular orbital (SOMO) in the open-shell [(^*t*Bu^PCP)­Pd] (a), [(^*t*Bu^PCP)­Au]^+^ (b), and [(CNC)­Au] (c) fragments. In each panel, the pie chart corresponding to the percentage composition of the MO in terms of the ligand and the metal atomic orbitals (*n* = 4 for [(^*t*Bu^PCP)­Pd]; *n* = 5 for [(^*t*Bu^PCP)­Au]^+^ and [(CNC)­Au]) is reported for each complex.

The composition of the SOMO of [(^*t*Bu^PCP)­Pd] (Figure 7a) reveals that the orbital is largely localized on the PCP pincer ligand (62.2%), with metal contributions arising from the 4d and 5p Pd atomic orbitals (20.5 and 17.3%, respectively). The SOMO of [(^*t*Bu^PCP)­Au]^+^ features analogous ligand contributions (64.3%), which is expected considering that the two metal fragments share the same pincer ligand (Figure 7b). Interestingly, however, the metal contributions are different: in the case of gold, we not only find contributions from both 5d and 6p atomic orbitals of Au (14.8 and 15.4%, respectively) but also unveil a 5.5% contribution from the 6s orbital of Au. This is not only consistent with the relativistically stabilized 6s orbital of gold,^67^ but also explains the increased degree of covalency for the gold complex compared to the isostructural [(^*t*Bu^PCP)­PdH] complex. In fact, the 6s contribution in the SOMO of [(^*t*Bu^PCP)­Au]^+^ leads to a more efficient charge transfer from H to Au, (due to the enhanced Au electrophilicity, see Δρ_1β_′ charge fluxes 0.29*e* (−26.7 kcal/mol) vs 0.24*e* (−17.4 kcal/mol) for [(^*t*Bu^PCP)­Au–H]^+^ and [(^*t*Bu^PCP)­Pd–H], respectively, Figure 7 and Table S3), and, in turn, to a more efficient charge delocalization from H which decreases its hydride character. As a result, an increased degree of covalency is expected for the Au–H bond, which accounts for the decrease in net bond polarization from −0.12*e* for the Pd complex to −0.08*e* for the Au complex. As shown in Figure 7c, this analysis also accounts for the low degree of polarity of the Au–H bond in [(CNC)­AuH]: the SOMO of this complex features largely decreased contributions from the ligand atomic orbitals (45.4%), and as a result, the 6s atomic orbital contribution at the metal site increases to 22.6%, leading to a further increase of the charge delocalization from H to Au (Δρ_1β_′ charge flux 0.31*e*, −30.2 kcal/mol, see Figure 7 and Table S3), enhancing the electron-sharing character of the Au–H bond (through lowering of the hydride H character) and reducing the degree of bond polarization to −0.06*e*. Remarkably, these results are consistent with the H 1s percentage contribution in the M–H bonding MOs, which varies significantly from 41% for [(^*t*Bu^PCP)­Pd–H], to 30% for [(^*t*Bu^PCP)­Au–H]^+^, and 23% for [(CNC)­Au–H] (see Figure S26), paralleling the decreasing hydride character of the H atom.

This analysis reveals a clear effect of both the metal and the ligand on the bond polarity of the M–H bonds. On one hand, replacing Pd with Au leads to an increased degree of M–H bond covalency due to the increased contributions of the stable 6s atomic orbital of gold. On the other hand, the [CNC]^2–^ ligand, likely due to the higher negative charge at the ligand sites, is less involved in the Au–H bond (and in electron charge delocalization) in the corresponding complex, leading to a decreased bond polarity.

These results provide a rationale for the fine-tuning of the bond polarity in metal–hydride complexes via both metal and ligand design, which is likely related to their different reactivities with O_2_. In fact, as discussed in the Introduction, [(CNC)­AuH] (lowest bond polarity) is not reactive toward O_2_, while [(^*t*Bu^PCP)­AuH]^+^ (intermediate bond polarity) and [(^*t*Bu^PCP)­PdH] (highest bond polarity) both react with O_2_. The former, however, reacts via a radical chain mechanism, while the latter does not. This suggests that the fine-tuning of the degree of the M–H bond polarity is essential not only to ensure efficient reactivity with dioxygen but also to control the nature of the mechanism.

## Conclusions

The rational design of homogeneous catalysts for the aerobic oxidation of organics is hindered by the restricted knowledge of how oxygen reacts with organometallic systems. A detailed mechanism of the reaction of oxygen with the Au­(III) hydride complex [(^*t*Bu^PCP)­Au­(III)–H]^+^, which proceeds to form the insertion product [(^*t*Bu^PCP)­Au­(III)–OOH]^+^, is computationally elucidated here, contributing to the emerging understanding of how oxygen reacts with metal-hydrides. The spin–orbit coupling inclusion in the calculations, as well as the bond nature/reactivity relationship analysis, are key to deeply understanding how O_2_ inserts into the Au­(III)–H bond. This reaction, although experimentally exhibiting kinetic behavior similar to that of previously reported Pd­(II)–H reactions, was found not to be fully consistent with any known O_2_ insertion mechanism. The O_2_ insertion into the Au­(III)–H bond has been found here to occur through a two-step hydrogen abstraction/recombination mechanism. At the recombination step, two channels are accessible: along the singlet PES, the Au­(III)–OOH product is formed in a radical-like process (“geminate recombination”); along the triplet PES, the [(^*t*Bu^PCP)­AuO_2_·]^+^ and ·OOH radicals are generated (“radical cage escape”), initiating radical chain pathways autoaccelerated by the hydrogen peroxide formed in the initiation, consistent with the experimental work. The radical chain mechanism has been unveiled to be driven by excess O_2_. At variance, for Pd­(II)–H, the recombination step, following the hydrogen abstraction step, occurs only along the singlet PES, with the Pd­(II)–OOH product forming through a nonradical process. Different reactivities with O_2_ are proven to be strictly related to the nature of the M–H (M = Au, Pd) bond. By a suitable computational protocol, based on combined EDA and CD-NOCV approaches, we provide a rationale for the fine-tuning of the bond polarity in metal–hydride complexes via both metal and ligand design. Although sharing a common covalent nature of the M–H bond, [(CNC)­AuH] (lowest Au–H bond polarity) is not reactive toward O_2_, while [(^*t*Bu^PCP)­AuH]^+^ (intermediate Au–H bond polarity) and [(^*t*Bu^PCP)­PdH] (highest Pd–H bond polarity) both react with O_2_. Fine-tuning of the extent of the M–H bond polarity is essential not only to ensure efficient reactivity with dioxygen, but also to control the mechanism, radical ([(^*t*Bu^PCP)­AuH]^+^) vs nonradical ([(^*t*Bu^PCP)­PdH]).

We believe that this work will contribute to the nascent knowledge base, which is of vital importance in the design and development of sustainable aerobic oxidation catalysis.

## Supplementary Material



## Notes

Metodology, additional tables (triplet/singlet single point energy of the optimized TSI SOC geometries, M–H (M = Au, Pd) bond analysis), figures (preliminary study on the O_2_ insertion mechanism, SOC structures of stationary points along the adiabatic PES, SOC structures of TSI SOC for the model and real complexes, SOC PES exploration through one-dimensional cuts of the two-dimensional SOC PES for the [(^*t*Bu^PCP)­AuH]^+^ reactivity with O_2_, spin densities of the metal-based radicals, SOC reaction profile for the OOH substitution by O_2_ with [(^*t*Bu^PCP)­PdH], electronic structure of the [(^*t*Bu^PCP)­AuH]^+^, [(^*t*Bu^PCP)­PdH], and [(CNC)­AuH] complexes), atomic displacements for the imaginary frequency of relevant transition states and xyz coordinates of all structures (PDF)
