# Supplementary material for: Mechanistic Insights into Molecular Oxygen Reactivity with Late Transition Metal–Hydride Bonds
Source: Inorg Chem. 2025 Jul 13;64(29):15193–205. doi: 10.1021/acs.inorgchem.5c02310 (PMC12308812; doi:10.1021/acs.inorgchem.5c02310)
Supplement: Supplementary file 1 [file ic5c02310_si_001.pdf]

Supporting Information

Mechanistic Insights into Molecular Oxygen Reactivity with Late Transition Metal-Hydride Bonds

Diego Sorbelli*[a], Leonardo Belpassi[b], Paola Belanzoni*[bc]

[a] *Diego Sorbelli*
Pritzker School of Molecular Engineering
University of Chicago 5640 South Ellis Avenue, Chicago, IL, 60615, United States
E-mail: dsorbelli@uchicago.edu

[b] *Leonardo Belpassi, Prof. Dr. Paola Belanzoni*
CNR Institute of Chemical Science and Technologies "Giulio Natta" (CNR-SCITEC)
Via Elce di Sotto, 8-06123 Perugia, Italy

[c] *Paola Belanzoni*
Department of Chemistry, Biology and Biotechnology
University of Perugia
Via Elce di Sotto, 8-06123 Perugia, Italy
E-mail: paola.belanzoni@unipg.it

Contents

Methodology **S3**
Figures S1-S13. The O_2 insertion mechanism – preliminary study **S5**
Figures S14-S16. SOC structures of intermediates and transition states along the adiabatic PES **S27**
Table S1. Triplet/singlet single-point energy of the optimized TS SOC geometry for the three complexes **S30**
Figure S17. TSI SOC structures of model and real complexes **S31**
Figures S18-S23. PES exploration with SOC: cutting the two-dimensional SOC PES for the $[(^{tBu}PCP)Au\text{-}H]^+$ complex reactivity with O_2. .... **S32**
Figures S24-S25. Spin delocalization in the three metal-based radicals - Reaction profile (SOC) for the OOH substitution by O_2 with [(PCP)Pd-H] **S43**
Table S2-S3. M-H (M = Au, Pd) bond analysis **S44**
Figure S26. Electronic structure of $[(^{tBu}PCP)AuH]^+$, $[(^{tBu}PCP)PdH]$, and [(CNC)AuH] **S45**
References **S48**
Atomic displacements for the imaginary frequency of relevant transition states **S49**
xyz geometries **S57**

Methodology

- **Natural Orbitals for Chemical Valence (NOCV) and Charge Displacement (CD) analysis**

Natural Orbitals for Chemical Valence (NOCV)[1,2] represents a suitable approach for describing the chemical bond. This approach is based on the rearrangement of the electron density occurring when a chemical bond is formed and such rearrangement can be expressed as electron density difference between the formed adduct (AB) and sum of the densities of the two non-interacting fragments (A and B) frozen in their adduct geometry. In this work, the doublet open shell $[M]^{0/+}$ and [H]**,** M= (^{tBu}PCP)Pd, (^{tBu}PCP)Au^{+}, (CNC)Au, fragments are considered to describe the M-H (M = Au, Pd) bond.

This deformation density can be brought into diagonal contributions in terms of NOCVs. In the NOCV scheme, the charge rearrangement taking place upon bond formation is obtained from the occupied orbitals of the two fragments suitably orthogonalized to each other and renormalized (*promolecule*). The resulting electron density rearrangement ($\Delta\rho'$) can be expressed in terms of NOCV pairs which are defined as the eigenfunctions of the so-called ‘‘valence operator’’[3-5] as follows:

$$\Delta\rho' = \sum_k \nu_k \left(|\phi_{+k}|^2 - |\phi_{-k}|^2\right) = \sum_k \Delta\rho'_k \qquad \text{[S1]}$$

where ϕ_{+k} and ϕ_{-k} are the NOCV pairs orbitals and $\nu_{\pm k}$ are the corresponding eigenvalues. Upon formation of the adduct from the promolecule, a fraction ν_k of electrons is transferred from the ϕ_{-k} to the ϕ_{-k} orbital (donor and acceptor orbitals, respectively).

The NOCV scheme can be coupled with the framework of the Charge Displacement (CD)[6] analysis. The CD analysis allows to quantify the amount of electronic charge that is transferred between the two fragments upon the formation of the A-B bond. The Charge Displacement function (Δq) can be defined as the partial progressive integration on a suitable z-axis of the deformation density $\Delta\rho'$:[7]

$$\Delta q(z) = \int_{-\infty}^{z} dz' \int_{-\infty}^{+\infty} \int_{-\infty}^{+\infty} \Delta \rho'(x, y, z') dx dy \qquad [S2]$$

The CD function, Δq(z), quantifies at each point of the chosen z-axis (which usually corresponds to the bond axis) the exact amount of electron charge that, upon formation of the bond, is transferred from the right to the left across a plane perpendicular to the bond axis through z.

When coupled with the NOCV scheme, the density rearrangement due to the bond formation between two fragments, ($\Delta\rho'$), is partitioned into different NOCV deformation densities ($\Delta\rho'_k$) and therefore one is able to quantify the charge transfer (CT) associated to each component. Note that only few of the NOCV pairs contributes to the chemical bond. Therefore, when the CD-NOCV analysis is carried out, usually only the first $\Delta\rho_k'$ components are investigated in order to understand which significant chemical contribution to the bond they represent.

Usually we choose to evaluate the charge transfer between A and B by taking the CD value at the "isodensity boundary", i.e. the z-point where equally valued isodensity surfaces of the isolated fragments become tangent.[7,8]

- **Energy Decomposition Analysis (EDA) and ETS-NOCV approach**

The Energy Decomposition Analysis (EDA)[9–11] has been used in this work to get insights into the M-H (M = Au, Pd) bonds in the $[(^{tBu}PCP)Au\text{-}H]^+$, $[(^{tBu}PCP)Pd\text{-}H]$, and [(CNC)Au-H] complexes. With this approach, the interaction energy between two fragments (in our case $[M]^{0/+}$ and [H], M= $(^{tBu}PCP)Pd$, $(^{tBu}PCP)Au^+$, (CNC)Au fragments) can be decomposed in different contributions as follows:

$$\Delta E_{\text{int}} = \Delta E^{Pauli} + \Delta E_{elst} + \Delta E_{oi} + \Delta E_{disp}$$

[S3]

where ΔE^{Pauli} corresponds the Pauli repulsion interaction between occupied orbitals on the two fragments, ΔE_{elst} represents the quasiclassical electrostatic interaction between the unperturbed

charge distribution of the fragments at their final positions, ΔE_{disp} takes into account the dispersion contribution and ΔE_{oi} is the orbital interaction, which arises from the orbital relaxation and the orbital mixing between the fragments, and accounts for electron pair bonding, charge transfer, and polarization.

The orbital interaction term ΔE_{oi} can be further decomposed within the NOCV[12] scheme into pairwise orbital contributions ($\Delta E_{oi} = \sum_k \Delta E_{oi}^k$) which associates an energy contribution (E_{oi}^k) to each NOCV deformation density ($\Delta\rho_k$).

The O_2 insertion mechanism – preliminary study

Based on the mechanistic and kinetic studies in ref.13, a possible hydrogen abstraction pathway for the O_2 insertion into the M-H (M = Au, Pd) bond has been studied starting with preliminary triplet (T) and open shell (unrestricted) singlet (os S) PES scans. The O_2 insertion mechanism is subsequently explored along the triplet and singlet pathways. Reaction free energy profiles for the hydrogen abstraction mechanism have been calculated along the diabatic open shell singlet, singlet and triplet PESs. Results are reported for [(^{tBu}PCP)Au-H]$^+$, [(^{tBu}PCP)Pd-H], and [(CNC)Au-H] in Figures S1-S13).

In the following calculations, the scalar zero-order regular approximation ZORA model was adopted to include relativistic effects. For all the stationary points along the triplet and the closed (restricted) and open shell (unrestricted) singlet pathways $\langle S^2 \rangle$ values were checked to assess whether spin contamination could influence the results. For triplet state as well as restricted singlet state structures $\langle S^2 \rangle$ values are very close to 2.0 and 0.0, respectively for all the stationary points, resulting in pure spin solutions. On the contrary, unrestricted singlet state structures revealed triplet spin contamination, corresponding to $\langle S^2 \rangle$ values ranging from 0.5 to 1.0. For correcting the mixed spin energies and removing the higher-multiplet spin components, the method proposed by Ovchinnikov and Labanowski[14] was adopted. This correction scheme has been applied to the energy of the stationary points along the unrestricted singlet pathway that revealed to be significantly contaminated with the triplet.

[(^{tBu}PCP)Au-H]$^+$

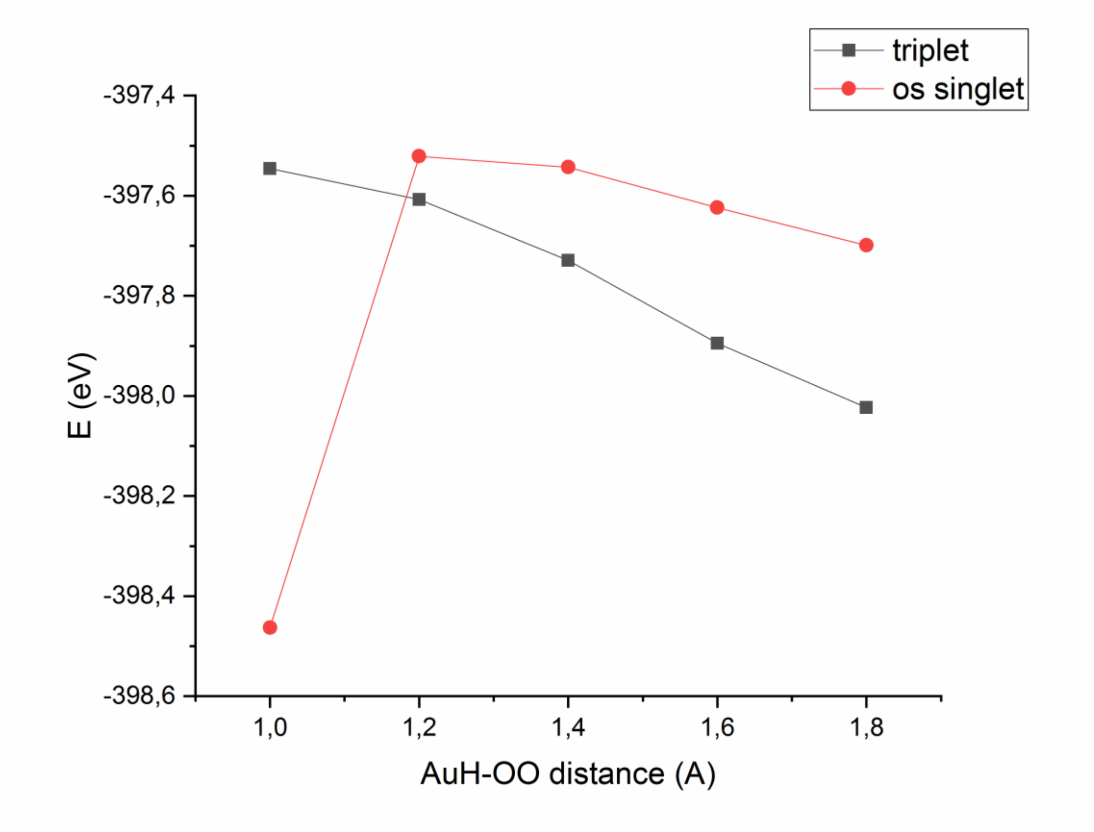

Figure S1. PESs scan (triplet and open shell singlet) using the AuH-OO distance (in Å) as reaction coordinate for O_2 insertion into the Au-H bond of the [(^{tBu}PCP)Au-H]$^+$complex.

The PESs scan (triplet and open shell singlet) using the AuH-OO distance as reaction coordinate for [(^{tBu}PCP)Au-H]$^+$complex is shown in Figure S1. As O_2 approaches the hydride, on the triplet PES the energy increases constantly, whereas on the open shell singlet PES the energy increases until an AuH-OO distance of about 1.2 Å and from this point a sharp lowering of the energy occurs leading to a stabilized intermediate structure (see INT in Figure S3). This crude PESs scan clearly suggests that to locate a triplet transition state the AuH-OO distance as reaction coordinate

is not enough. A two-dimensional triplet PES has been calculated using AuH-O2O1 and Au-O1O2 distances as reaction coordinates (Figure S2).

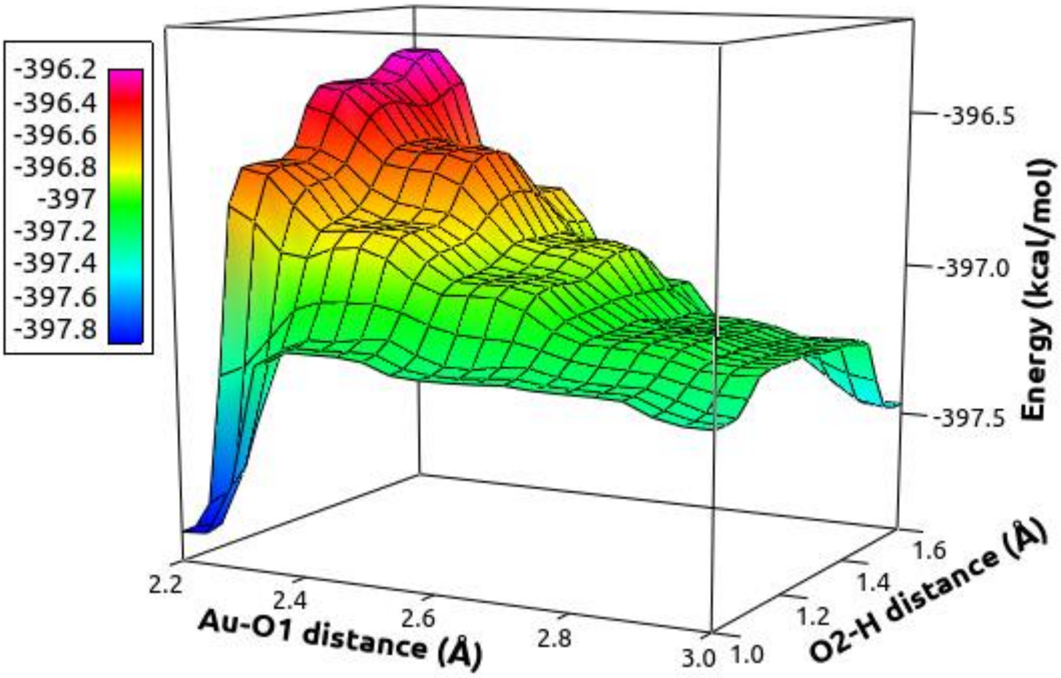

Figure S2. Two-dimensional triplet PES for the H abstraction/OOH rebound steps for [(^{tBu}PCP)Au-H]$^+$. The Au-O1 and O2-H bond distances, as defined in Scheme S1, are employed as reaction coordinates.

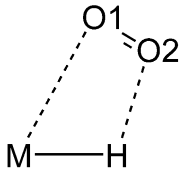

Scheme S1. Definition of the reaction coordinates for the two-dimensional PESs (M = Au, Pd).

From Figure S2, we observe that, starting from RC, the minimum energy path is found along the O2-H distance, describing the H abstraction step, which occurs via an energy barrier. After H abstraction, a very flat path can be seen along the Au-O1 coordinate, describing the OOH rebound step through the oxygen atom not bonded to H, leading to the final insertion product.

From the topology of this PES a triplet transition state and product could be located. Reaction free energy profiles for the hydrogen abstraction mechanism have been calculated along the diabatic open shell singlet, singlet and triplet PESs and are shown in Figure S3.

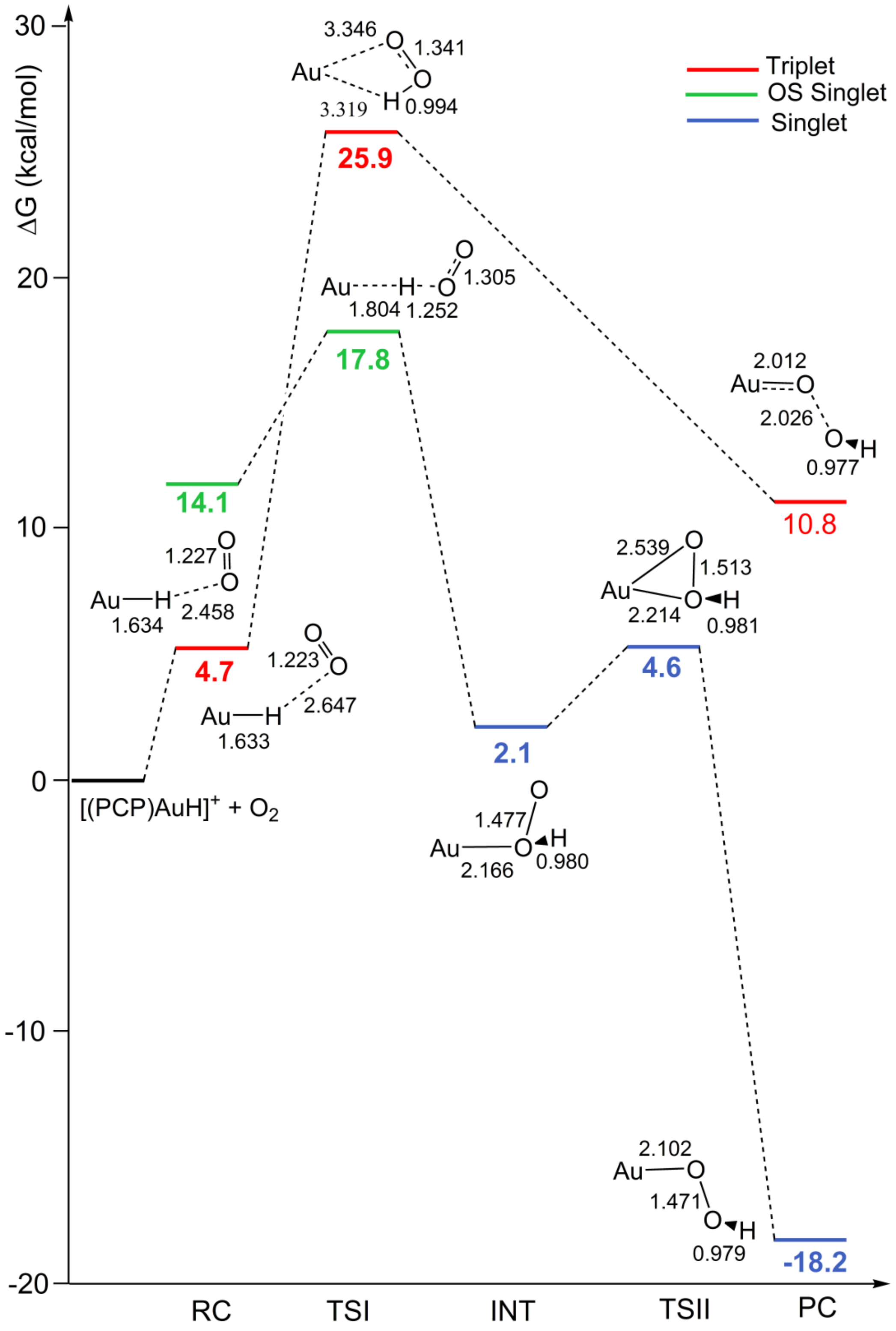

Figure S3. Reaction free energy profiles (triplet in red, singlet in blue and open shell singlet spin in green stationary points) and schematic structures (with relevant geometrical parameters in Å) for the hydrogen abstraction mechanism with $[(^{tBu}PCP)Au\text{-}H]^{+}$. ΔG values (in kcal/mol) refer to the energy of the isolated reactants in their ground spin state taken as zero.

The reactant complex (RC^{T}), involving the formation of a relatively weakly bound $[(^{tBu}PCP)Au\text{-}H]^{+}$ - O_2 complex, has been calculated in its triplet ground state as endergonic (by 4.7 kcal/mol) and in its excited open shell singlet state ($RC^{os\ S}$), significantly higher in energy (14.1 kcal/mol). A singlet RC^{S} calculation could not be converged. A transition state could be located both on the open shell singlet PES ($TSI^{os\ S}$) and on the triplet PES, but not on the singlet PES (Figure S3). Indeed, both $RC^{os\ S}$ and $TSI^{os\ S}$ energies suffer from triplet spin contamination ($\langle S^2 \rangle = 1.00$ and 0.77, respectively), therefore the method mentioned above was applied to correct the calculated too low energies. The resulting reaction energy profiles are shown in Figure S4.

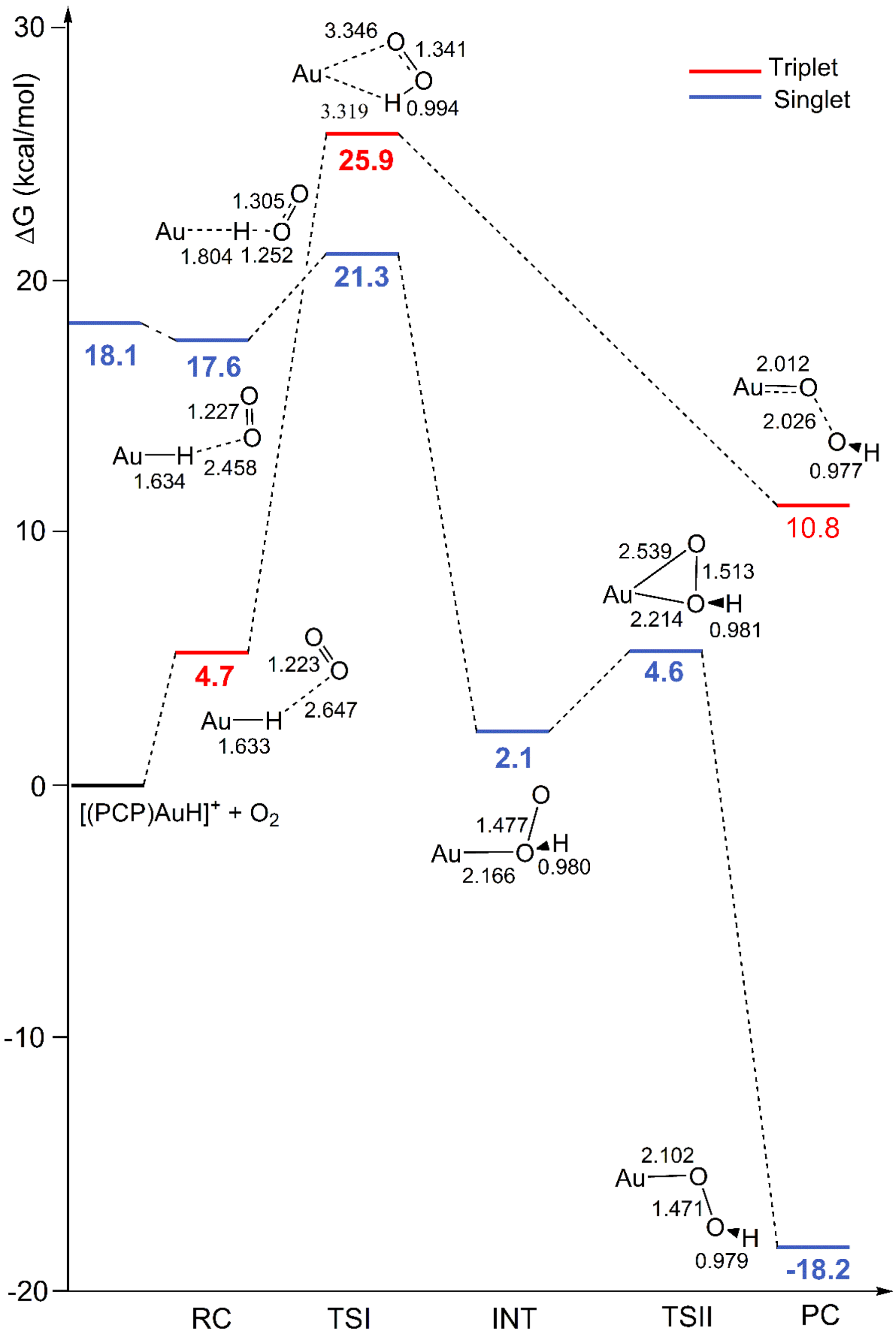

Figure S4. Reaction free energy profiles (triplet in red, singlet with spin correction in blue stationary points) and schematic structures (with relevant geometrical parameters in Å) for the hydrogen abstraction mechanism with $[(^{tBu}PCP)Au\text{-}H]^{+}$. ΔG values (in kcal/mol) refer to the energy of the isolated reactants in their ground spin state taken as zero.

From Figure S3 a different mechanism can be clearly observed along the triplet and singlet pathways. Along the triplet path, the reaction evolves through a concerted abstraction of the hydrogen atom from the gold center by O_2 and formation of the HOO fragment bonding to the metal, overcoming an energy barrier of 25.9 kcal/mol through the TSI^T transition state and leading to the final hydroperoxo product PC^T in an endergonic step (10.8 kcal/mol). Instead, the reaction mechanism along the singlet PES involves a two-step process: 1) a hydrogen atom abstraction resulting in the formation of an intermediate species, $[(^{tBu}PCP)Au\text{-}OHO]^+$ (INT^S), in which the hydrogen atom is bonded to the oxygen coordinated to the Au center (with an energy barrier amounting to 17.8 kcal/mol (21.3 kcal/mol including spin contamination correction) in a nearly thermoneutral process, ΔG = 2.1 kcal/mol); 2) a facile rearrangement step where, from the intermediate, exergonic formation of the final product (PC^S) takes place (-18.2 kcal/mol), overcoming a low barrier (2.5 kcal/mol) associated to the transition state $TSII^S$ for the simultaneous breaking of the bond with proximal oxygen and bond forming with distal oxygen atom. From Figure S3 we could expect the spin crossing to be located before $TSI^{os\ S}$ transition state (at lower energy), thus the free energy activation barrier could be lower than 17.8 kcal/mol (21.3 kcal/mol with spin contamination correction).

$[(^{tBu}PCP)Pd\text{-}H]$

The same procedure has been applied to calculate stationary points along the triplet and singlet PESs for the O_2 insertion into the Pd(II)-H bond in the $[(^{tBu}PCP)Pd\text{-}H]$ complex.

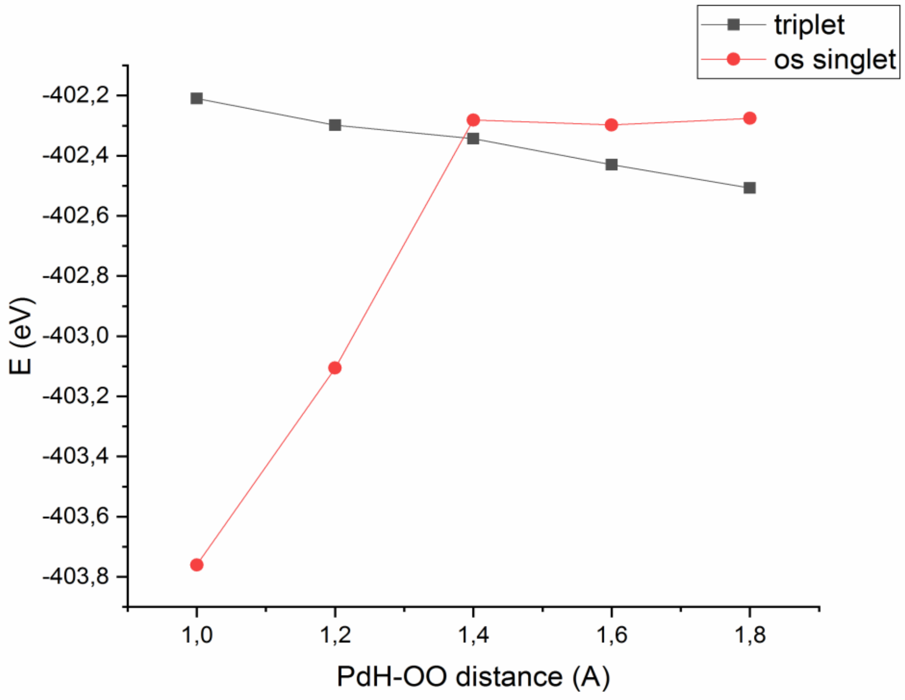

Figure S5. PESs scan (triplet and open shell singlet) using the PdH-OO distance (in Å) as reaction coordinate for O_2 insertion into the Pd-H bond of the [(^{tBu}PCP)Pd-H] complex.

From the PESs scans, upon approach of O_2 to the metal hydride, on the triplet PES the energy continuously increases, whereas on the open shell singlet PES the energy remains nearly constant until a PdH-OO distance of about 1.4 Å and from this point a sharp lowering of the energy leads to a stabilized intermediate (Figure S5). Apart from an earlier spin crossing for [(^{tBu}PCP)Pd-H] (more "reactant-like" transition state) and an initially flat open shell singlet PES, the PESs scans for Au-H and Pd-H are qualitatively similar. Analogously, this PESs scan clearly suggests that to locate a triplet transition state the PdH-OO distance as reaction coordinate is not sufficient and a two-dimensional triplet PES has been calculated using PdH-O2O1 and Pd-O1O2 distances as reaction coordinates (Figure S6).

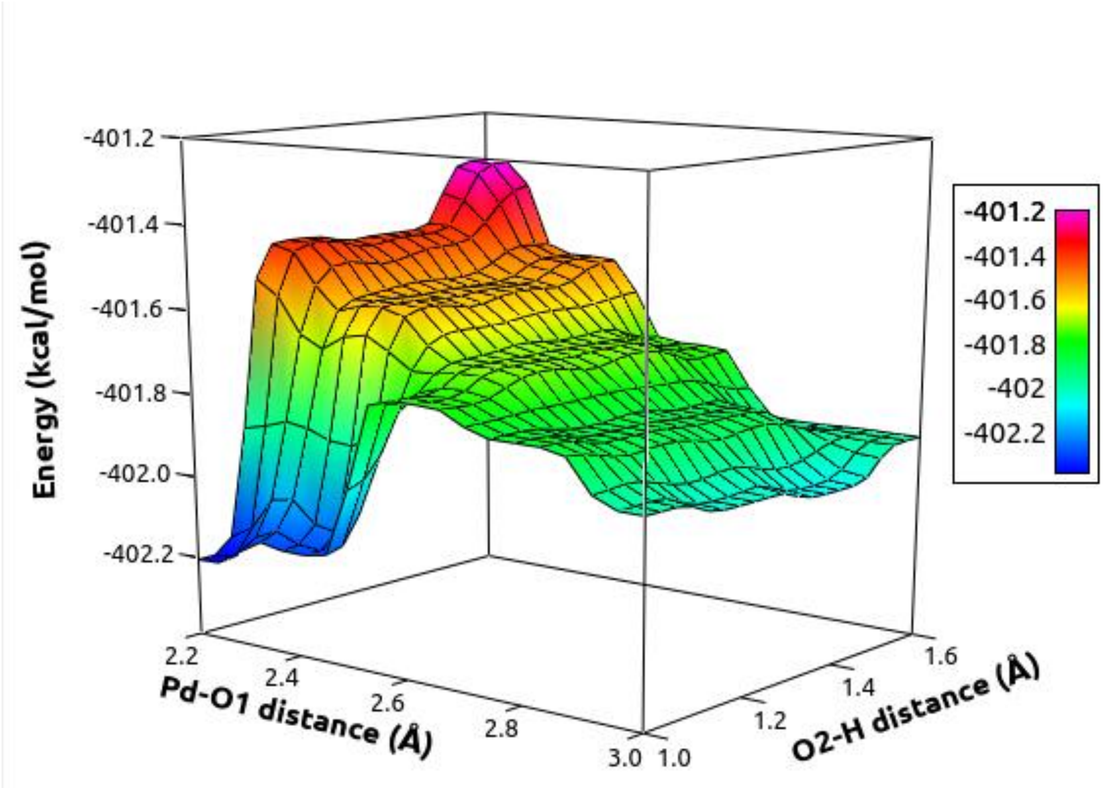

Figure S6. Two-dimensional triplet PES for the H abstraction/OOH rebound steps for [(^{tBu}PCP)Pd-H]. The Pd-O1 and O2-H bond distances, as defined in Scheme S1, are employed as reaction coordinates.

From the topology of this PES, starting from RC, the minimum energy path is similarly found along the O2-H distance (H abstraction step), which occurs very easily (almost barrierless). After H abstraction, a minimum energy path is along the Pd-O1 coordinate, describing the OOH rebound step through the oxygen atom not bonded to H, via a small energy barrier, leading to the final insertion product.

Similarly, the reaction free energy profiles are calculated along the diabatic triplet, open shell singlet and singlet PESs and are depicted in Figure S7.

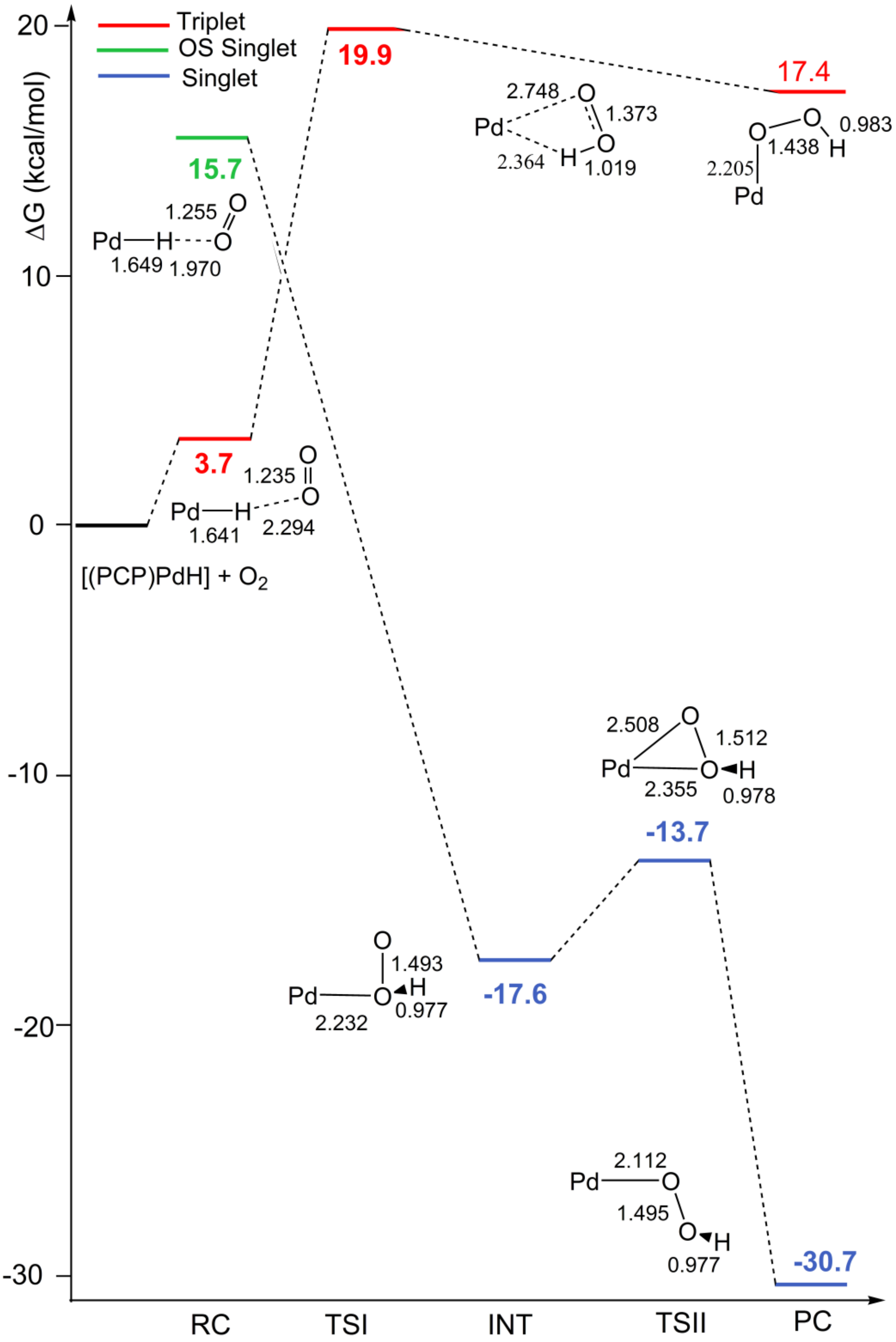

Figure S7. Reaction free energy profiles (triplet in red, singlet in blue and open shell singlet in green stationary points) and schematic structures (with relevant geometrical parameters in Å) for the hydrogen abstraction mechanism with [(^{tBu}PCP)Pd-H]. ΔG values (in kcal/mol) refer to the energy of the isolated reactants in their ground spin state taken as zero.

The reactant complex has been calculated in its triplet ground state (RC^{T}) and open shell singlet excited state (RC$^{os\ S}$), but not in the singlet state (RC^{S}). A transition state on the open shell singlet PES could not be computed, despite all the attempts to find it out, consistent with the flat PES in Figure S5. It is relevant to underline that similar results have been obtained for the same [(^{tBu}PCP)Pd-H] complex by Sicilia and coworkers[15] even using a different exchange-correlation functional (B3LYP), where a crossing occurring in the vicinity of the triplet transition state (TS^{T}) has been calculated that lies lower in energy. Analogous to the above results, the authors found that the mechanism along the singlet PES involves as a result of the hydrogen atom abstraction the formation of a very stable intermediate (INT^{S}), in which hydrogen atom is bonded to the oxygen coordinated to the Pd center. From Figure S7, a spin crossing could be expected before reaching TS^{T}, which lies at 19.9 kcal/mol above the free reactants. Similar to the gold complex, the RC$^{os\ S}$ energy suffers from triplet spin contamination ($<S^2> = 1.00$), therefore the method mentioned above was applied to correct the calculated too low energy. The resulting reaction energy profiles are shown in Figure S8.

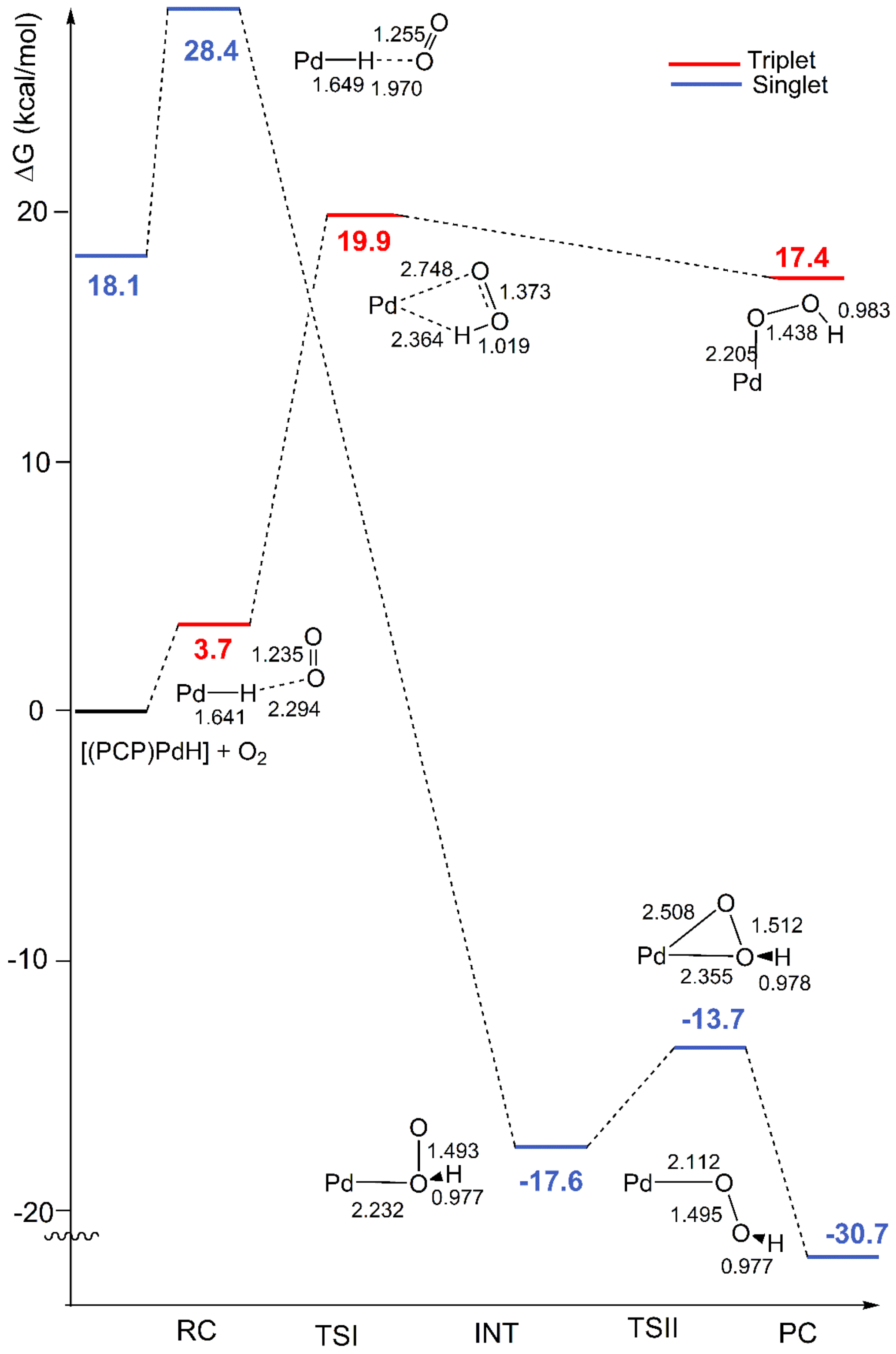

Figure S8. Reaction free energy profiles (triplet in red, singlet with spin correction ref. in blue stationary points) and schematic structures (with relevant geometrical parameters in Å) for the hydrogen abstraction mechanism with [(^{tBu}PCP)Pd-H]. ΔG values (in kcal/mol) refer to the energy of the isolated reactants in their ground spin state taken as zero.

Analogous to $[(^{tBu}PCP)Au\text{-}H]^+$, along the triplet path the reaction mechanism is a concerted one-step process, with an energy barrier of 19.9 kcal/mol for the hydrogen abstraction from the palladium center by O_2, yielding a thermodynamically unstable hydroperoxo product PC^T (17.4 kcal/mol), which suggests a facile reversible process. Along the singlet PES, instead, the reaction mechanism is a two-step process, involving 1) a hydrogen abstraction forming an intermediate species INT^S (pure singlet spin state) with an activation barrier lower than 15.7 kcal/mol (28.4 kcal/mol including the spin contamination correction, Figure S10) in a highly exergonic process (ΔG = -17.6 kcal/mol) and 2) a facile INT^S rearrangement step leading to the thermodynamically highly stable product PC^S.

The second transition state ($TSII^S$) is located on the singlet PES with a lower activation free energy barrier of 3.9 kcal/mol, showing a facile OOH recombination leading to the hydroperoxide product (PC^S). Notably, the overall O_2 insertion into the Pd-H bond along the singlet PES is highly exergonic by -30.7 kcal/mol.

[(CNC)Au-H]

Following the same procedure, stationary points along the triplet and singlet PESs have been calculated for the O_2 insertion into the Au(III)-H bond in the [(CNC)Au-H] complex.

The hydrogen abstraction mechanism by O_2 has been explored starting with triplet (T) and open shell (unrestricted) singlet (os S) potential energy surface (PES) scans to rationalize the ligand, i.e. ^{tBu}PCP vs. CNC, crucial role on gold reactivity with molecular oxygen (Figure S9).

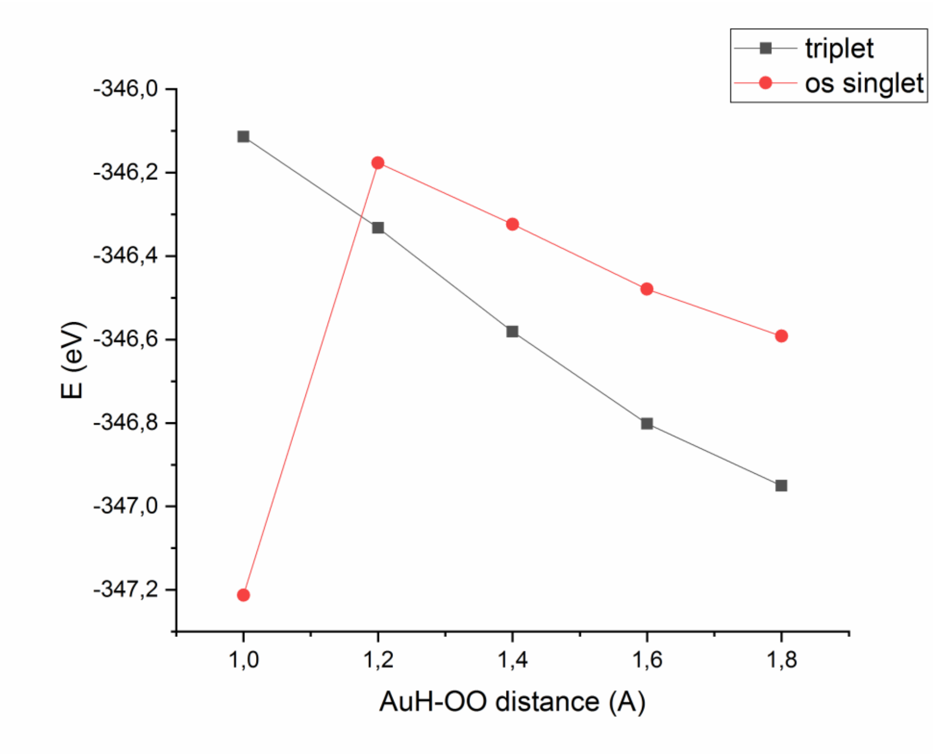

Figure S9. PESs scan (triplet and open shell singlet) using the AuH-OO distance (in Å) as reaction coordinate for O_2 insertion into the Au-H bond of the [(CNC)Au-H] complex.

Similar to [(^{tBu}PCP)Au-H]$^+$ and [(^{tBu}PCP)Pd-H], as O_2 approaches the metal hydride, on the triplet PES the energy continuously increases, whereas on the open shell singlet PES the energy increases until an AuH-OO distance of less than 1.2 Å and from this point the energy sharply decreases leading to a stabilized intermediate. Although the PESs scans for Au-H in [(CNC)Au-H] and [(^{tBu}PCP)Au-H]$^+$ are qualitatively similar, a later spin crossing for [(CNC)Au-H] has been found, suggesting a more "product-like" transition state. A two-dimensional triplet PES has been calculated using AuH-O2O1 and Au-O1O2 distances as reaction coordinates (Figure S10).

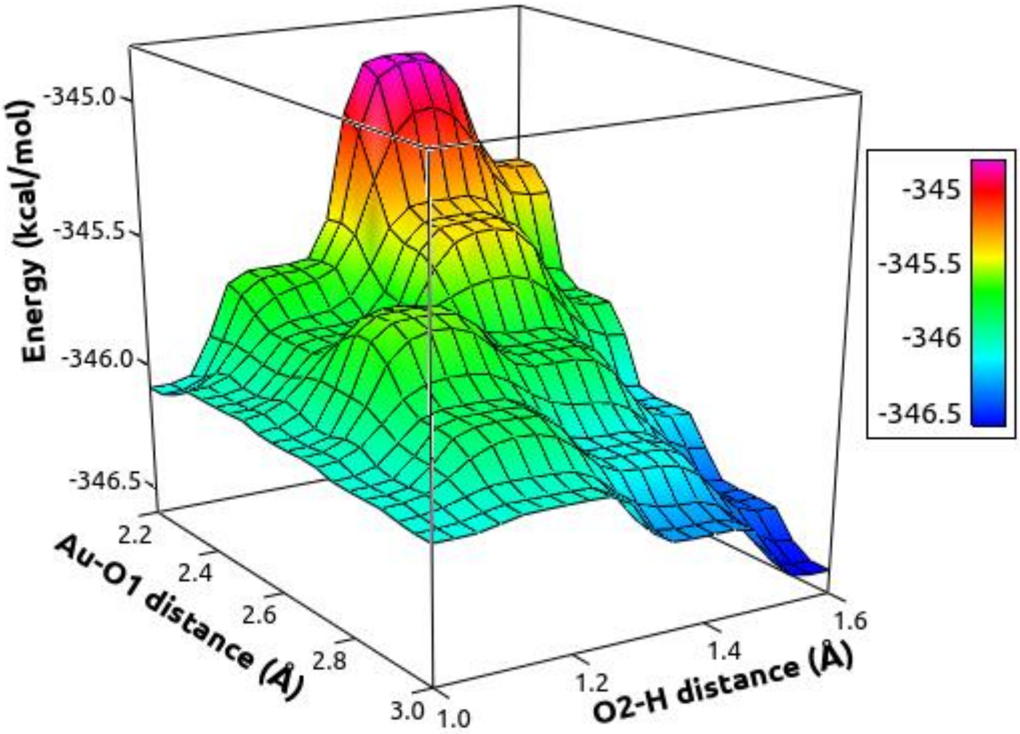

Figure S10. Two-dimensional triplet PES for the H abstraction/OOH rebound steps for [(CNC)Au-H]. The Au-O1 and O2-H bond distances, as defined in Scheme S1, are employed as reaction coordinates.

Starting from RC, the topology of the PES shows that a minimum energy path is similarly found along the O2-H distance, which occurs via a continuous energy increase and a flat region. The PES remains also very flat at the same energy level all along the Au-O1 coordinate, overall hardly leading to the final insertion product.

Reaction free energy profiles along the triplet, open shell singlet and singlet diabatic PESs are shown in Figure S11.

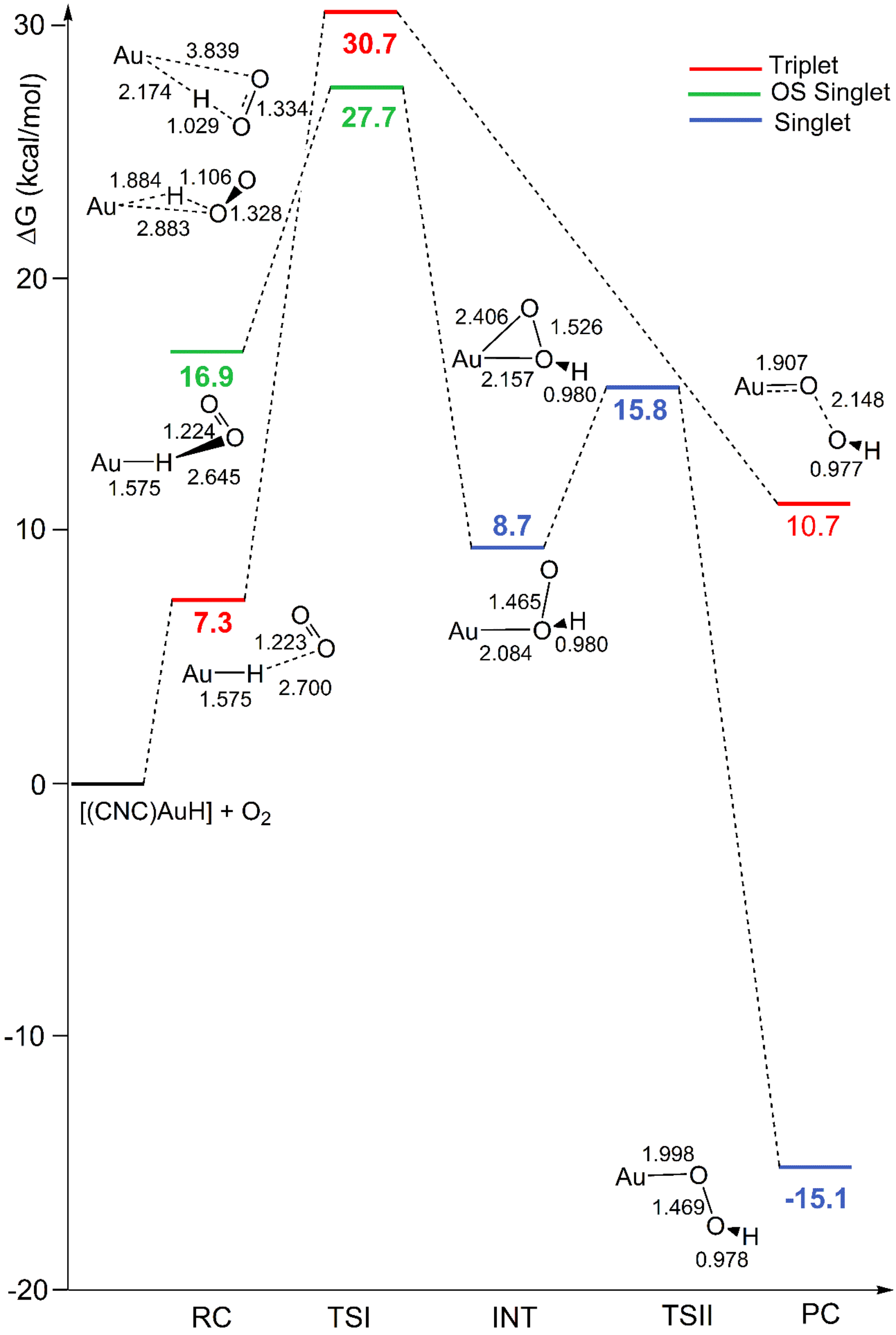
ΔG (kcal/mol)
30
20
10
0
-10
-20
Triplet
OS Singlet
Singlet
30.7
27.7
16.9
7.3
8.7
15.8
10.7
-15.1
[(CNC)AuH] + O2
RC
TSI
INT
TSII
PC
Au 3.839 O 2.174 H 1.029 O 1.334
1.884 1.106 H O Au O 1.328 2.883
2.406 O 1.526 Au O H 2.157 0.980
1.907 Au O 2.148 O H 0.977
O 1.224 O Au H 1.575 2.645
O 1.223 O Au H 1.575 2.700
O 1.465 H Au O 2.084 0.980
1.998 Au O 1.469 O H 0.978

Figure S11. Reaction free energy profiles (triplet in red, singlet in blue and open shell singlet in green stationary points) and schematic structures (with relevant geometrical parameters in Å) for the hydrogen abstraction mechanism with [(CNC)Au-H] complex. ΔG values (in kcal/mol) refer to the energy of the isolated reactants in their ground spin state taken as zero.

Analogous to the previous complexes, the reactant complex (RC^{T}), involving the formation of a relatively weakly bound [(CNC)Au-H] - O_2 complex, has been calculated in its triplet ground state as endergonic by 7.3 kcal/mol and only in its open shell singlet state ($RC^{os\ S}$), significantly higher in energy (16.9 kcal/mol). A transition state could be located both on the open shell singlet PES ($TSI^{os\ S}$) and on the triplet PES (TSI^{T}) (Figure S11). Similarly, both $RC^{os\ S}$ and $TSI^{os\ S}$ species showed a triplet spin contamination ($\langle S^2 \rangle = 1.00$ and 0.50, respectively), and their energies were corrected resulting in the reaction energy profiles depicted in Figure S12.

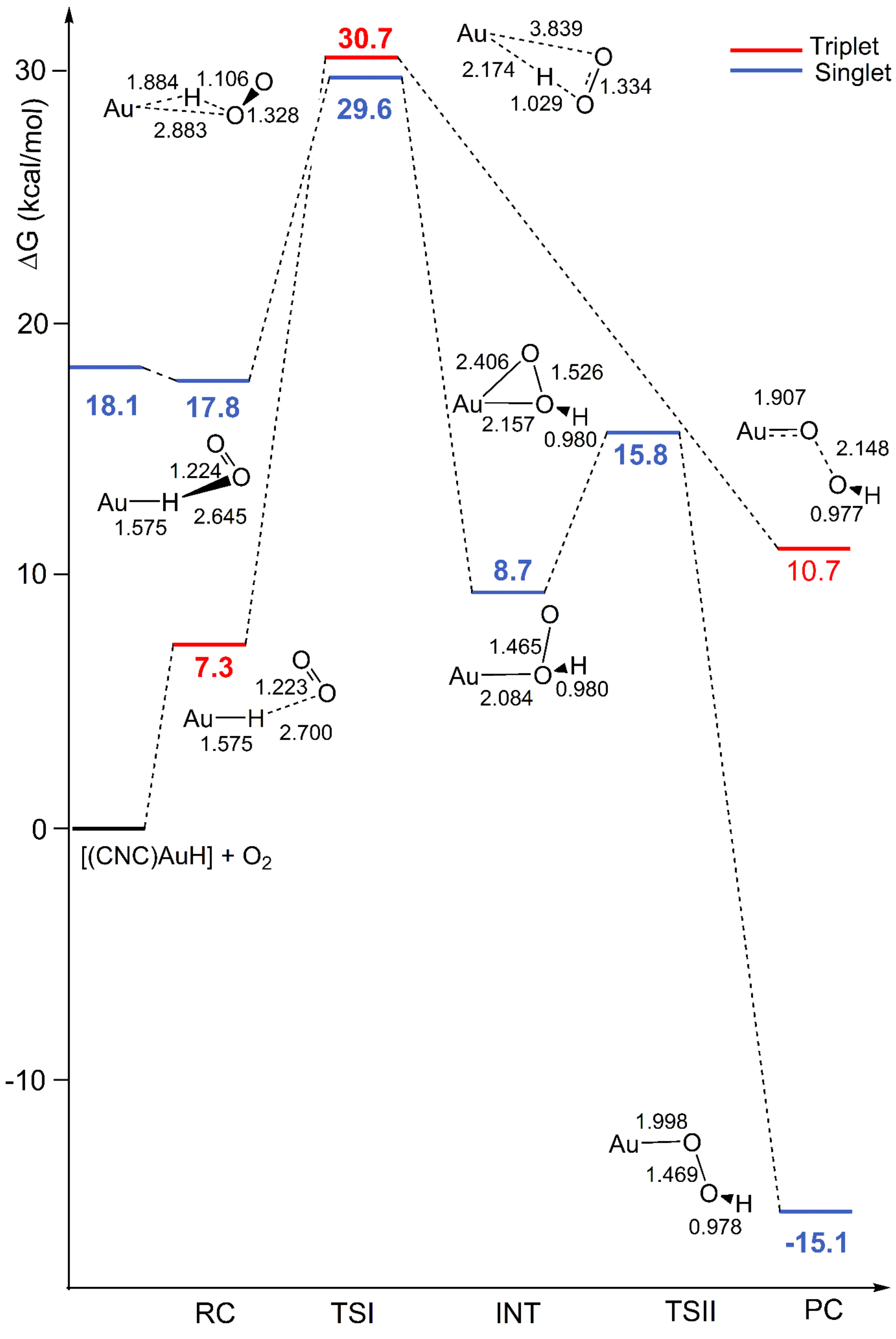
ΔG (kcal/mol)
30
20
10
0
-10
Triplet
Singlet
30.7
29.6
18.1
17.8
7.3
8.7
15.8
10.7
-15.1
[(CNC)AuH] + O2
RC
TSI
INT
TSII
PC
1.884
1.106
1.328
2.883
3.839
2.174
1.334
1.029
1.224
2.645
1.575
1.223
2.700
1.575
2.406
1.526
2.157
0.980
1.465
2.084
0.980
1.907
2.148
0.977
1.998
1.469
0.978

Figure S12. Reaction free energy profiles (triplet in red, singlet with spin correction in blue stationary points) and schematic structures (with relevant geometrical parameters in Å) for the hydrogen abstraction mechanism with [(CNC)Au-H] complex. ΔG values (in kcal/mol) refer to the energy of the isolated reactants in their ground spin state taken as zero.

Likewise, a different mechanism is observed along the triplet (one-step) and singlet (two-step) pathways. Along the triplet path, the concerted abstraction of the hydrogen atom from the gold center by O_2 and formation of the HOO fragment bonding to the metal, requires however overcoming a high energy barrier of 30.7 kcal/mol for the formation of the TSI^T transition state and leading to the final product PC^T in an endergonic step (10.7 kcal/mol). Along the singlet PES, the hydrogen atom abstraction resulting in the formation of an intermediate species, [(CNC)Au-OHO] (INT^S), in which the hydrogen atom is bonded to the oxygen coordinated to the Au center, occurs through a high activation energy barrier of 27.7 kcal/mol (29.7 kcal/mol including the spin contamination correction) in a significantly endergonic process, ΔG = 8.7 kcal/mol. Notably, the intermediate species INT^S is not efficiently stabilized, lying at almost the same energy as that of PC^T. The rearrangement step where, from the intermediate, exergonic formation of the final product (PC^S) takes place (-15.1 kcal/mol), with a low barrier (7.1 kcal/mol) associated to the transition state $TSII^S$, would be a feasible step. From Figure S11 we should analogously expect the spin crossing to occur before $TSI^{os\ S}$ transition state. However, the activation energy barriers on both the triplet and singlet PESs are very high, consistent with the lack of reactivity with O_2 experimentally reported for this complex.

To summarize: the main findings highlight a different mechanism along the triplet and singlet pathways for all the three complexes (Scheme S2).

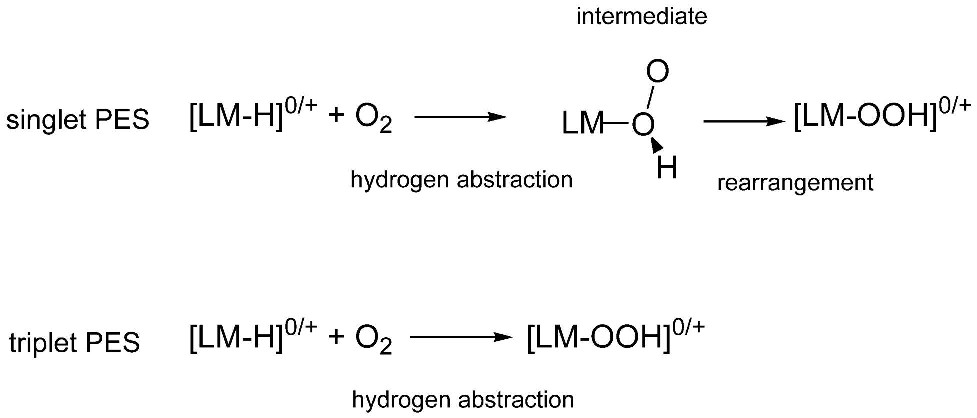

Scheme S2. Schematic reaction mechanisms on the diabatic singlet and triplet PESs for molecular oxygen insertion into M-H bond of $[(^{tBu}PCP)Au\text{-}H]^+$, $[(^{tBu}PCP)Pd\text{-}H]$ and [(CNC)Au-H].

Along the triplet path, the reaction evolves through a concerted abstraction of the hydrogen atom from the gold center by O_2 and formation of the HOO fragment bonding to the metal in a highly endergonic step. Instead, the reaction mechanism along the singlet PES involves a two-step process: : 1) a hydrogen atom abstraction resulting in the formation of an intermediate species, $[(^{tBu}PCP)Au\text{-}OHO]^+$, $[(^{tBu}PCP)Pd\text{-}OHO]$, and [(CNC)Au-OHO], in which the hydrogen atom is bonded to the oxygen coordinated to the metal center; 2) a facile rearrangement step where, from the intermediate, exergonic formation of the final product, $[(^{tBu}PCP)Au\text{-}OOH]^+$, $[(^{tBu}PCP)Pd\text{-}OOH]$, and [(CNC)Au-OOH], takes place, overcoming a lower barrier associated to the transition state for the simultaneous breaking of the bond with proximal oxygen and bond forming with distal oxygen atom. The stabilized intermediate species (singlet spin state) forms after the crossing between triplet and singlet PESs. Quantitatively, relevant differences emerge between the three complexes. For $[(^{tBu}PCP)Au\text{-}H]^+$, the energy barrier for the hydrogen abstraction amounts to 25.9 kcal/mol on the triplet PES and 21.3 kcal/mol on the singlet PES, in a nearly

thermoneutral process (ΔG = 2.1 kcal/mol) leading to the intermediate species. For [(^{tBu}PCP)Pd-H], an energy barrier for the hydrogen atom abstraction of 19.9 kcal/mol and 28.4 kcal/mol on the triplet and singlet PES, respectively, is calculated, in a highly exergonic step to intermediate formation (ΔG = -17.6 kcal/mol). For [(CNC)Au-H], the hydrogen abstraction step requires overcoming a high energy barrier of 30.7 and 29.7 kcal/mol on the triplet and singlet PES, respectively, in a significantly exergonic process leading to intermediate species (ΔG = 8.7 kcal/mol).

These preliminary results are fully consistent with the experimental evidence that hydrogen abstraction reactivity of Au-H bonds with O_2 strongly depends on the ligand nature (hydrogen abstraction with [(CNC)Au-H] is not feasible), whereas the reaction mechanism (radical/non radical) strongly depends on the metal nature, through modulation of the M-H bond nature. Indeed, the same hydrogen abstraction mechanism has been found for Au and Pd, which experimentally has a radical character for Au and a non-radical character for Pd. However, these results suggest that the observed difference might not be due to the M• and •OOH recombination rate, which has been hypothesized slower in [(^{tBu}PCP)Au-H]$^+$ than in [(^{tBu}PCP)Pd-H], or to the radical cage escape, but rather to the metal effect which critically affects both the energy stabilization and the M-OHO bond nature of the hydrogen abstraction transition state and intermediate.

O_2 metal coordination/H migration mechanism

As an alternative, the O_2 metal coordination/H migration mechanism has been explored. In this path, first molecular oxygen coordinates to the metal, then hydrogen atom migration leads to O_2 insertion into the M-H bond. This mechanism has been explored for [(^{tBu}PCP)Au-H]$^+$ and [(^{tBu}PCP)Pd-H], starting from triplet and open shell singlet PESs scans.

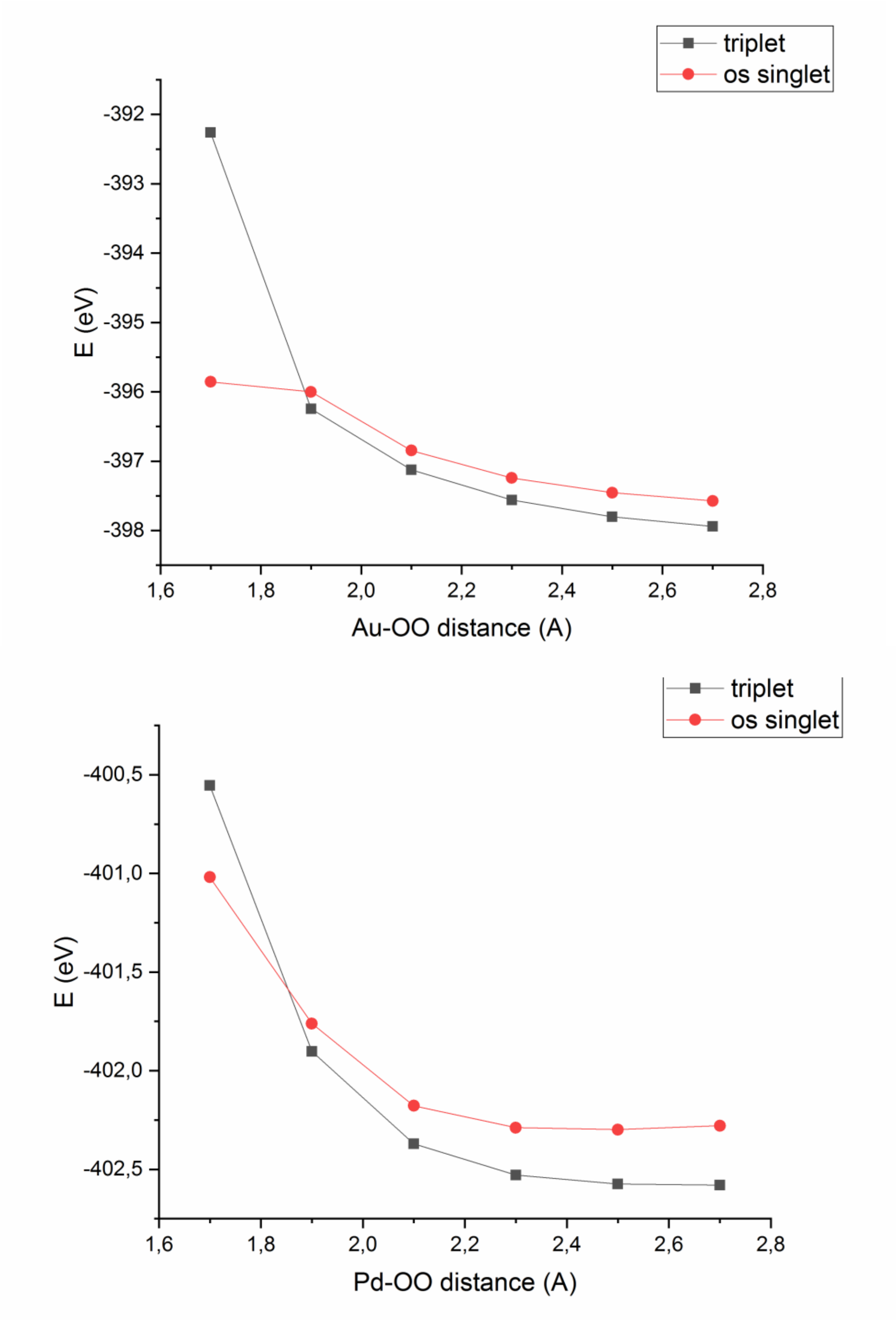

Figure S13. PESs scan (triplet and open shell singlet) using the Au-OO (top) and Pd-OO (bottom) distance (in Å) as reaction coordinate for O_2 insertion with $[(^{tBu}PCP)Au\text{-}H]^+$ and $[(^{tBu}PCP)Pd\text{-}H]$.

In Figure S13 the plot of the PESs scan using the M-OO (M = Au, Pd) distance (in Å) as reaction coordinate for the O_2 metal coordination pathway is shown. The energy continuously increases on both PESs, not allowing to locate any Au or Pd stable adduct. This mechanism has not been considered further.

SOC structures of intermediates and transition states along the adiabatic PES

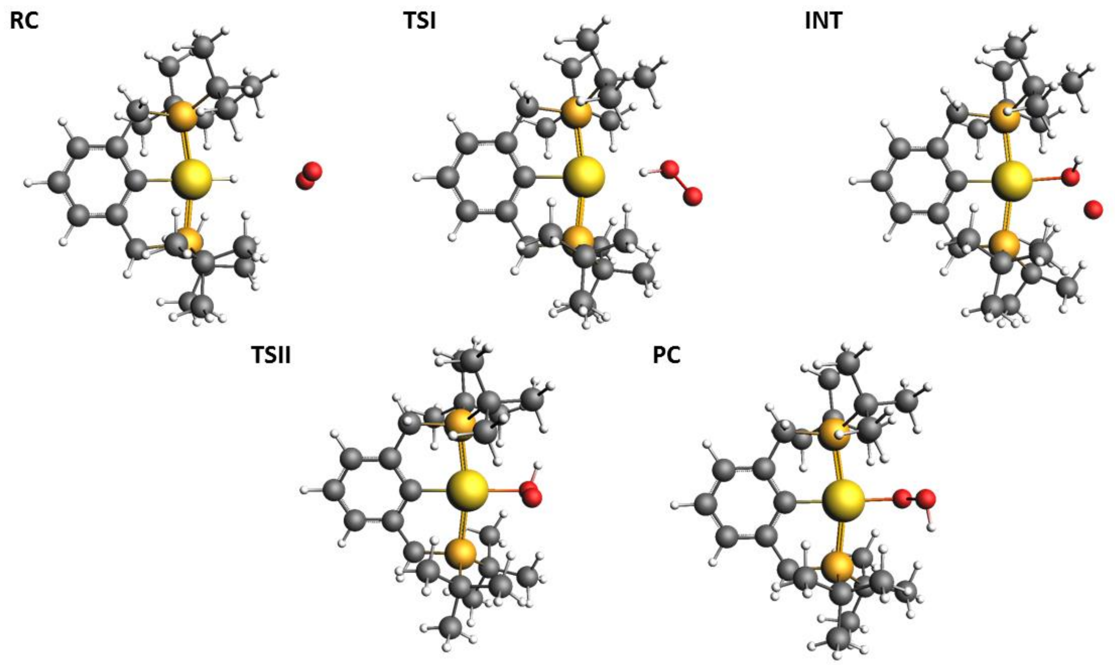

Figure S14. Calculated SOC structures of minima and transition states localized along the relativistic SOC path for the insertion of molecular oxygen into the M-H bond of the [(^{tBu}PCP)Au-H]$^+$ complex (Figure 1 in the main text).

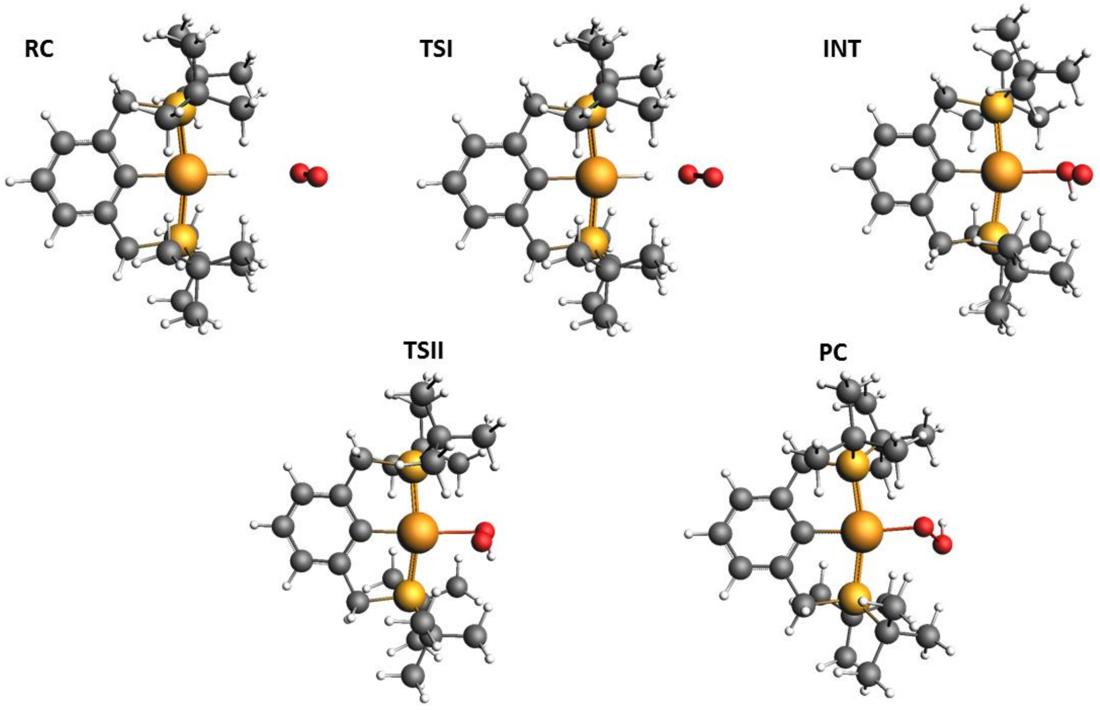

Figure S15. Calculated SOC structures of minima and transition states localized along the relativistic SOC path for the insertion of molecular oxygen into the M-H bond of the [(^{tBu}PCP)Pd-H] complex (Figure 1 in the main text).

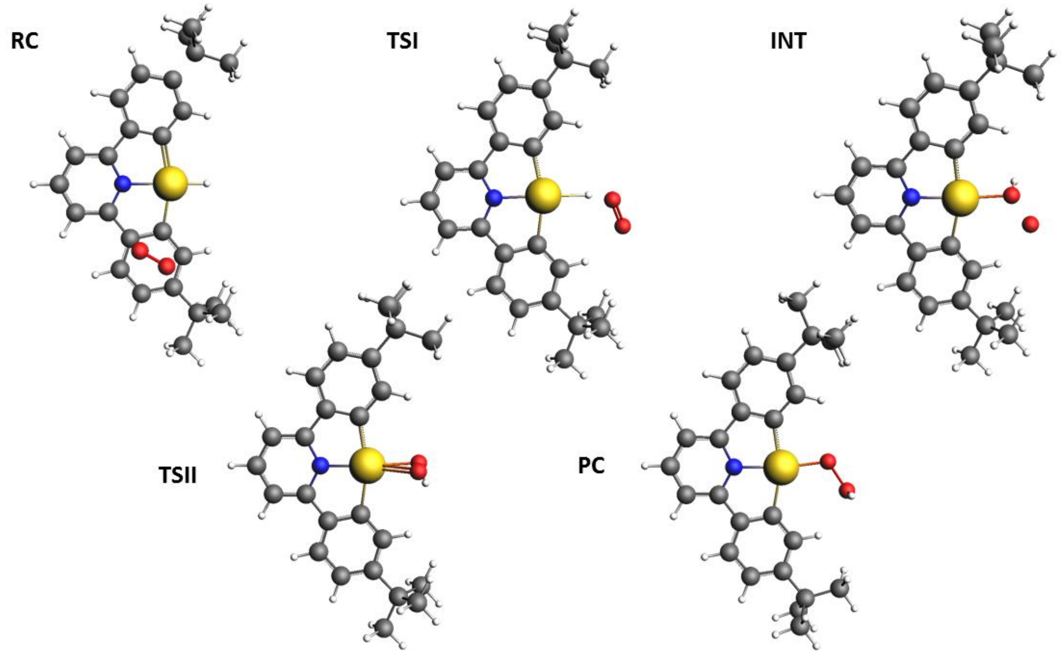

Figure S16. Calculated SOC structures of minima and transition states localized along the relativistic SOC path for the insertion of molecular oxygen into the M-H bond of the [(CNC)Au-H] complex (Figure 1 in the main text).

	Singlet (eV)	Open shell singlet (eV)	Triplet (eV)	ΔE (T-S) (kcal/mol)
[(^{tBu}PCP)Au-H]$^+$	-397.37	-397.56 (<S^2 > = 0.74)	-397.50	-3.1
[(^{tBu}PCP)Pd-H]	-402.22	-402.22	-402.34	-2.7
[(CNC)Au-H]	-346.02	-346.02	-346.05	-0.8

Table S1. Singlet, open shell singlet and triplet spin state single-point energy calculations on the corresponding optimized TS SOC geometry for the [(^{tBu}PCP)Au-H]$^+$, [(^{tBu}PCP)Pd-H] and [(CNC)Au-H] complexes (Figure 1 in the main text). ΔE values refer to triplet/singlet relative energy (triplet minus singlet) (T-S).

In Figure S17, the TSI SOC structures for the model ([(PCP)AuH]$^+$, [(PCP)PdH], [(CNC')AuH]) and real ([(^{tBu}PCP)AuH]$^+$, [(^{tBu}PCP)PdH] , [(CNC)AuH]) complexes are compared.

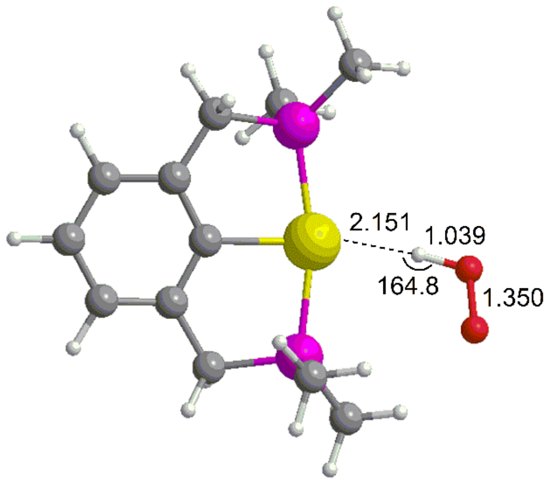

[(PCP)AuH]$^+$

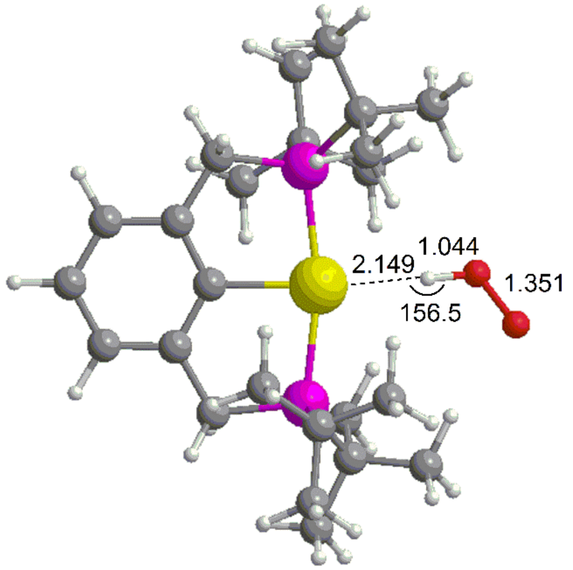

[(^{tBu}PCP)AuH]$^+$

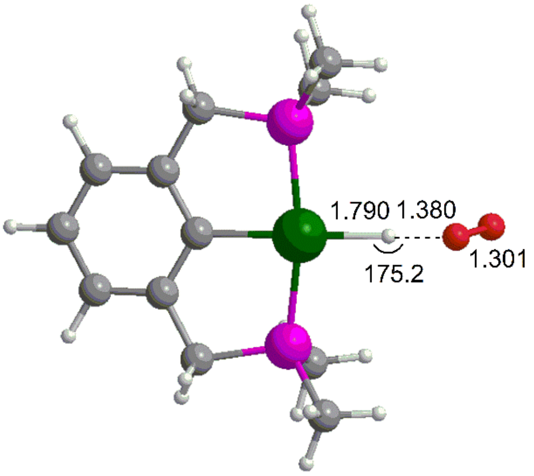

[(PCP)PdH]

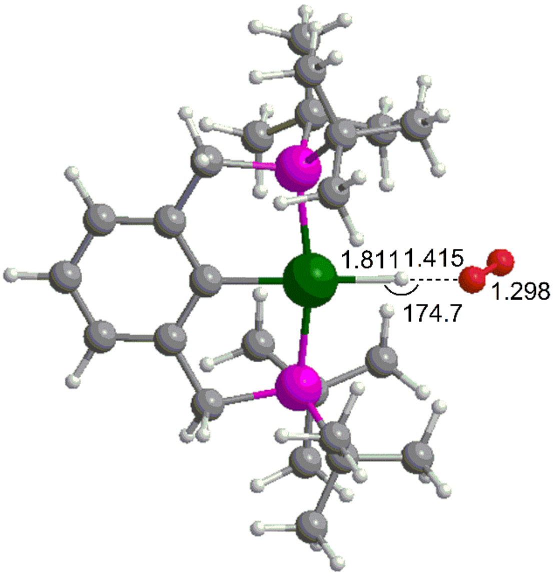

[(^{tBu}PCP)PdH]

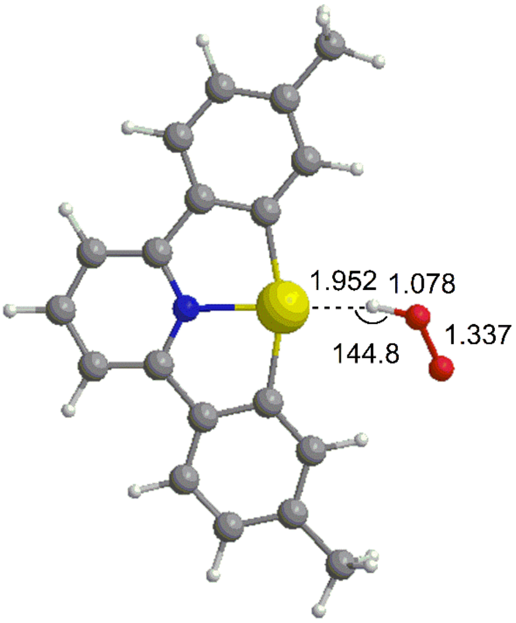

[(CNC')AuH]

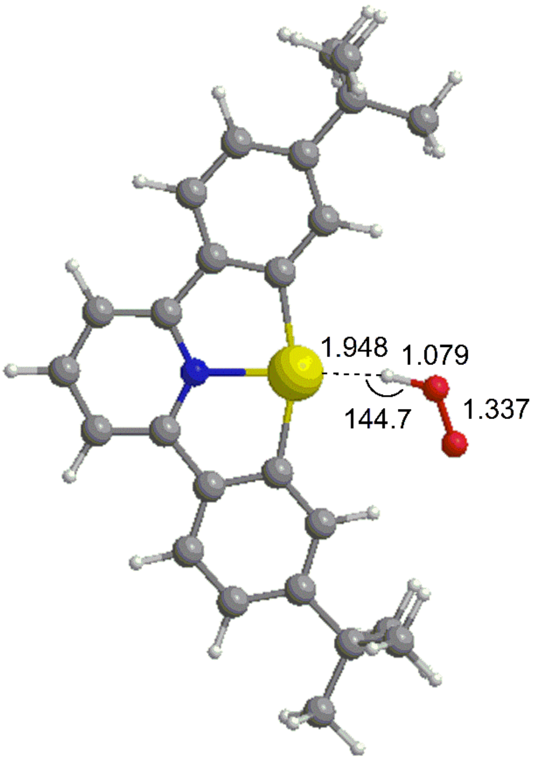

[(CNC)AuH]

Figure S17. TSI SOC structures (with relevant geometrical parameters in Å and degrees) for the dioxygen insertion with model ([(PCP)AuH]$^+$, [(PCP)PdH], [(CNC')AuH] on the left) and real ([(^{tBu}PCP)AuH]$^+$, [(^{tBu}PCP)PdH] , [(CNC)AuH] on the right) complexes.

The TSI SOC geometries in FigureS17 show that no significant structural change is introduced. We find that the simplified structures are very reasonable models for all the real complexes.

PES exploration with SOC: cutting the two-dimensional SOC PES for the [(^{tBu}PCP)Au-H]$^+$ complex reactivity with O_2.

A major drawback in TS SOC approach is that in ADF analytical frequency calculations using SOC ZORA are not supported, and very time consuming numerical frequency calculations need to be performed to confirm that the optimized saddle point geometry (TS SOC) has one imaginary frequency which, for the large systems under study, are practically unaffordable. Alternatively, to make sure that the optimized saddle point geometries are the desired transition state structures that connect the reactants RC and the intermediate species INT, one may perform a two-dimensional SOC PES scan. Similarly, due to the SOC high computational cost, two-dimensional PESs describing the O_2 insertion into the M-H bond are far from being practicable. However, possible solutions to solve the above highly demanding computational problems are: i) using a simplified model of the system; ii) exploring more viable one-dimensional cuts of the two-dimensional SOC PES, which can give an idea about the reaction paths landscape. It should be emphasized that, although the resulting one-dimensional SOC PESs are crude simplifications of the actual multidimensional surfaces, a reasonable guess for the transition state can be obtained which may serve to confirm the structure of the calculated TS SOCs. We applied these two methodologies for [(^{tBu}PCP)Au-H]$^+$, and complete details of the study ii) are reported here. The main results suggest that the formation of the [(^{tBu}PCP)Au•]$^+$ radical after hydrogen abstraction can be only stabilized through interaction with the •OOH radical.

The SOC optimized geometries of the minimum energy points along the hydrogen abstraction path (RC, INT and PC) are sketched in Scheme S3.

To find the most suitable reaction coordinate for studying the reaction in a one-dimensional PES, one possibility is to use the Au…HOO distance, since it largely changes from RC to INT (Scheme 3, values in red colour).

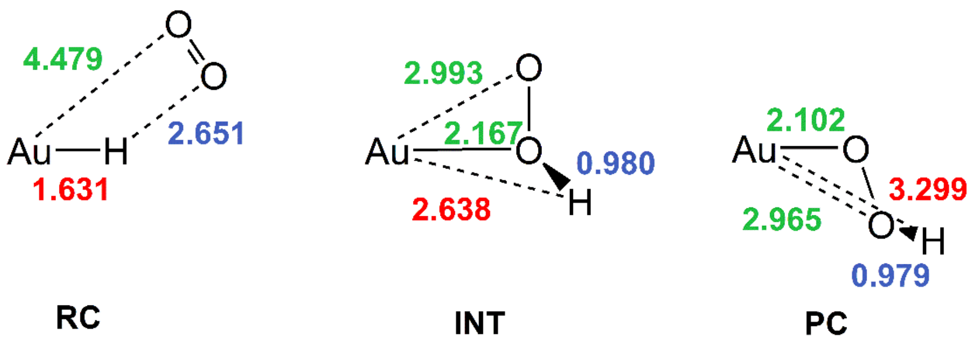

Scheme S3: SOC optimized geometrical structure of RC (left), INT (middle) and PC (right). Au-HOO (red), AuH-OO (blue) and Au-OOH (green) bond distances (in Å) as plausible reaction coordinates are shown.

Starting from the INT structure, the Au…HOO distance has been decreased from 2.6 Å (INT) to 1.6 Å (RC) (Scheme S3), building up a SOC PES scan (Figure S18).

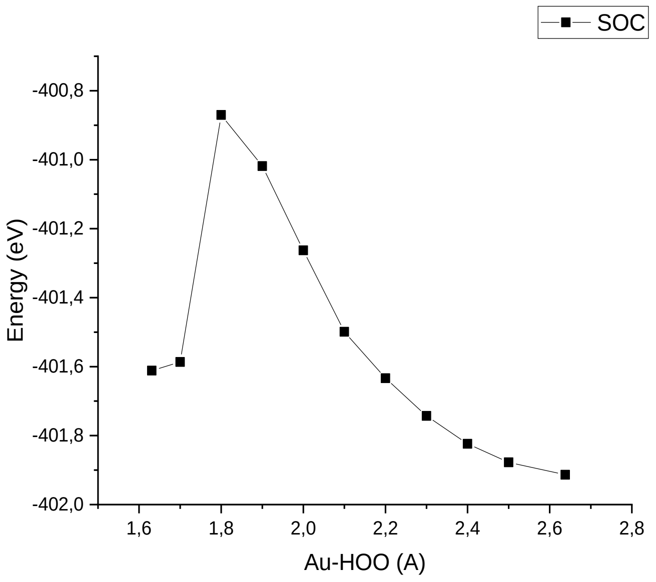

Figure S18: PES scan (SOC) from INT to RC structure using the Au…HOO distance (Å) as reaction coordinate.

The maximum energy value (-400.8704 eV) is calculated for the Au-HOO distance of 1.8 Å, corresponding to the structure shown in Scheme S3 (left), which is compared to the TS SOC structure (energy value -400.9905 eV) (Scheme S4 middle).

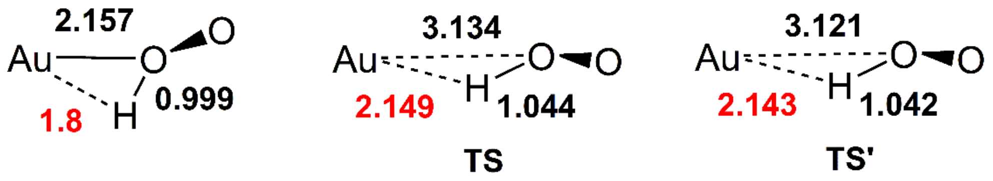

Scheme S4: SOC optimized geometrical structure at fixed Au-HOO distance of 1.8 Å, corresponding to the maximum energy value in Figure S15 (left), calculated TS SOC structure (middle), calculated TS SOC structure starting from the structure at fixed Au-HOO distance of 1.8 Å (right).

In the TS structure the Au-H, Au-O and O-H distances are larger and the incipient OOH is reoriented more parallel to the complex plane. In both structures the O-H distance (0.999 and 1. 044 Å, respectively) indicates that the bond between oxygen and hydrogen is forming. As it is evident from both geometry and energy ($\Delta E = 2.8$ kcal/mol) of these two points, the Au-HOO 1.8 Å guess structure is not too far from the actual TS. This structure has been used as a starting point for the TS search (it has been submitted to full optimization) and a TS' structure (E = -400.9904 eV) could be found which is also shown in Scheme S4 (right). Indeed, TS and TS' are isoenergetics and feature a very similar structure. The important (sharp) decrease of the energy after the Au-HOO 1.8 Å distance means that the Au-HOO bond lenght is not a good reaction coordinate. However, the above comparison between the Au-HOO 1.8 Å maximum energy, TS and TS' structures and Figure S18 suggest that the TS structure could be envisaged as the transition state for an initial H abstraction and subsequent barrierless OOH rebound through the oxygen atom which is bonded to H or as the transition state for a concerted H abstraction from Au and OOH rebound through the closest oxygen atom. Indeed, starting from the TS structure and selecting the Au-OHO distance as a reaction coordinate, which is decreased from 3.134 (TS) to 2.167 Å (INT), the SOC PES scan depicted in Figure S19 could be constructed, which demonstrates that the rebound step through the oxygen atom bonded to hydrogen is actually barrierless.

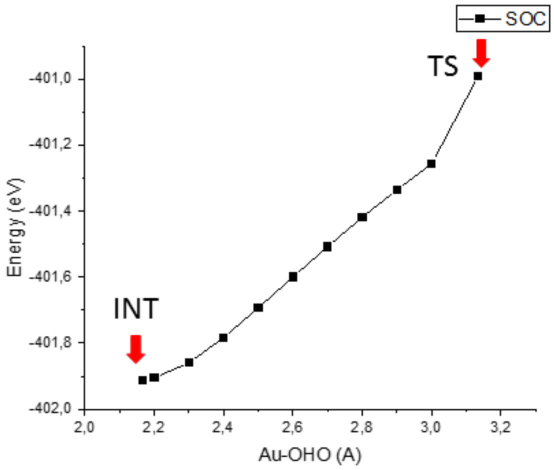

Figure S19: PES scan (SOC) from TS (Scheme S4) to INT (Scheme S3) structure using the Au…OHO distance (Å) as reaction coordinate.

Alternatively, the AuH…OO distance has been considered (Scheme S3, values in blue colour). A similar PES scan has been performed starting from the RC structure, where the AuH…OO distance has been decreased from 2.6 Å (RC) to 1.0 Å (INT) (Scheme S3), building up a SOC PES scan (Figure S20).

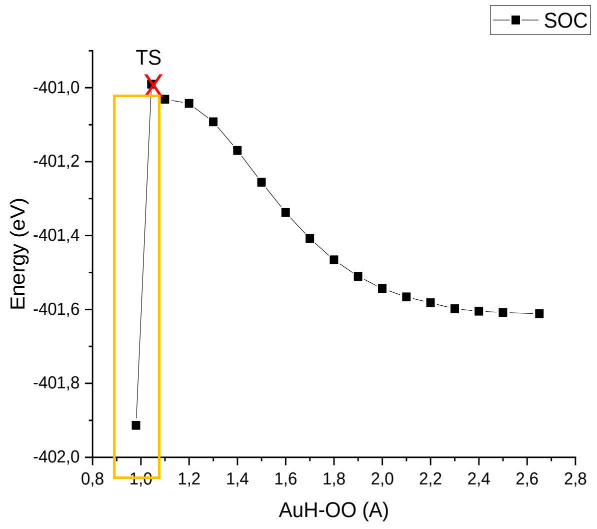

Figure S20: PES scan (SOC) from RC to INT structure using the AuH…OO distance (Å) as reaction coordinate. The TS SOC and INT energies and AuH…OO distances are shown within the orange panel.

The PES shape indicates a smooth increasing of the energy until a plateau is reached at AuH-OO distances of 1.2 and 1.1 Å. The TS SOC and INT energies and AuH…OO distances are also shown within the orange panel. The structure at 1.1 Å AuH-OO distance (energy value -401.0314 eV) is compared to the calculated TS SOC structure (AuH-OO distance = 1.044 Å, energy value -400.9905 eV) in Scheme S5.

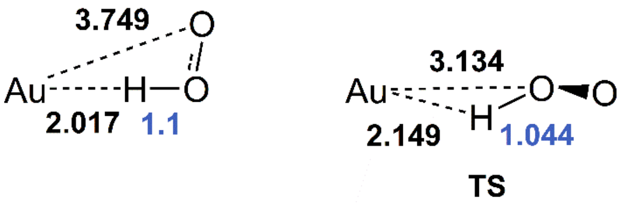

Scheme S5: SOC optimized geometrical structure at fixed AuH-OO distance of 1.1 Å, corresponding to the plateau energy value in Figure S17 (left), and calculated TS SOC structure (right).

In both structures the O-H distance (1.1 and 1. 044 Å, respectively) indicates that the bond between oxygen and hydrogen is forming and the main difference resides in the HOO orientation, enabling (TS) or not enabling (O-H 1.1 Å) a Au-OHO interaction. The fixed AuH-OO 1.1 Å distance structure suggests that from this point on the one-dimensional PES (Figure S20), a deviation of the Au-H-O bond from linearity would lead to INT via TS. Notably, the two structures in Scheme S5 are almost isoenergetics (ΔE = 0.9 kcal/mol). This finding strongly suggests that formation of the $[(^{tBu}PCP)Au\bullet]^{+}$ radical from hydrogen abstraction can be only stabilized through interaction with the resulting •OOH radical.

To search for a possible reaction path which could directly lead from the reactants to the product, the Au…HOO distance can also be used, which largely changes from RC to PC (Scheme S3, values in red colour).

Starting from the PC structure, the Au…HOO distance has been decreased from 3.1 Å (PC) to 1.6 Å (RC) (Scheme S3), building up a SOC PES scan (Figure S21).

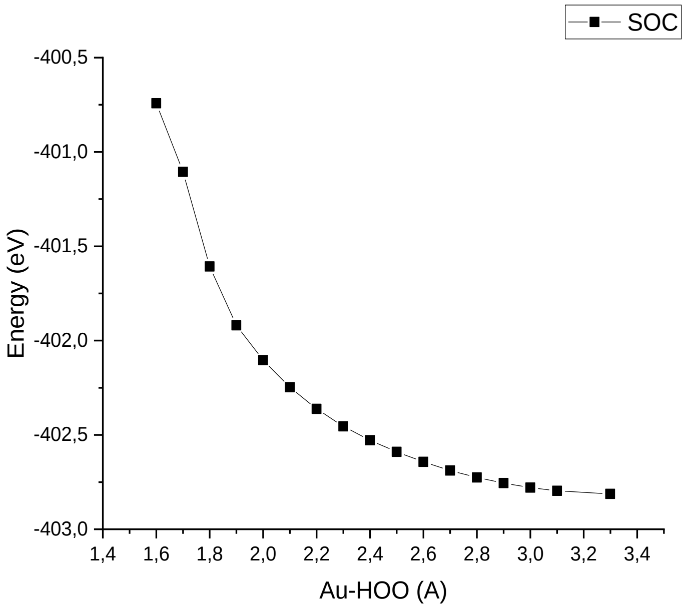

Figure S21: PES scan (SOC) from PC to RC structure using the Au…HOO distance (Å) as reaction coordinate.

The SOC PES energy increases constantly and the RC structure could not be recovered. The energy value (-400.7418 eV) calculated for the Au-HOO distance of 1.6 Å corresponds to the structure shown in Scheme S6, which is very different from both the RC and TS structures and is at higher energy than TS (ΔE = 5.7 kcal/mol).

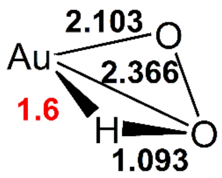

Scheme S6: SOC optimized geometrical structure at fixed Au-HOO distance of 1.6 Å, corresponding to the maximum energy value in Figure S21.

As expected, attempt to use this structure as the starting point for a TS searching was unsuccessfull, giving back the PC structure. The Au-HOO bond lenght is not a good reaction coordinate in this case, suggesting that a first hydrogen abstraction followed by a OOH rebound is likely to occur, consistent with experiment.

Nudget Elastic Band (NEB) approach

Given the unsymmetrical shape of the one-dimensional PESs shown above, the Nudget Elastic Band (NEB) method has been applied. The Nudget Elastic Band (NEB) method[16] is used to identify suitable reaction coordinates for this mechanism, where the two-dimensional PES can be very flat, and to find a rough approximation of the reaction path which is built as a set of images created by performing a linear interpolation between the initial (RC) and final (INT or PC) systems. With this method, a rough saddle point representing the transition state between the reactant and the product state can be envisaged. Through this method, a set of images is created by performing a linear interpolation between the initial (RC) and final (INT or PC) systems, ensuring that the images are evenly distributed along the path by connecting adjacent images with equally stiff springs. In Figure S22 ten images have been computed with NEB

from RC (initial system) to INT (final system). At each image, a single point calculation in the triplet, singlet, open shell singlet spin states and SOC state has been performed, allowing to construct crude approximations of the corresponding PESs.

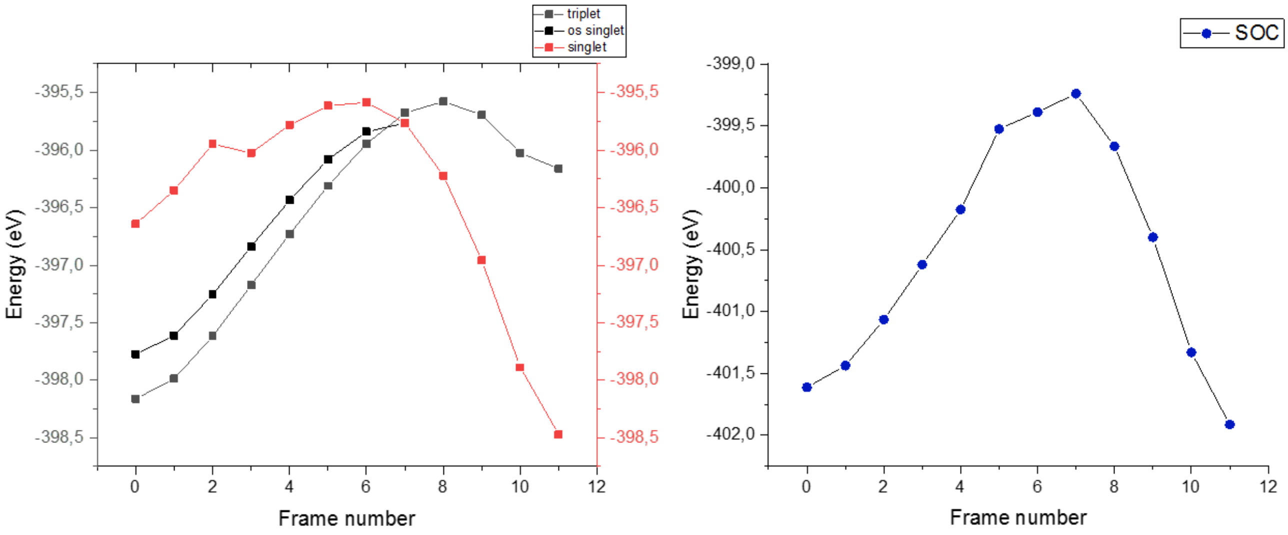

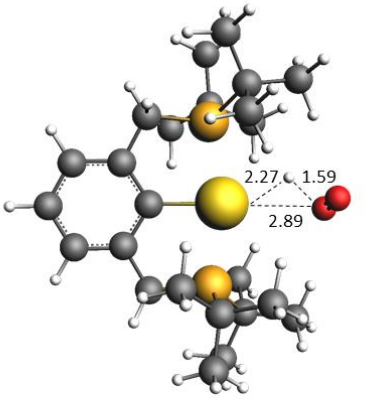

Figure S22: Reaction path computed with NEB using **RC** as the initial (frame number 0) and **INT** as the final (frame number 11) systems. For each image along the path a single point calculation has been performed in the triplet, singlet, open shell singlet spin states (left) and

SOC state (right). Image 7 corresponding to both triplet/singlet crossing and SOC maximum energy is also shown (distances in Å).

In Figure S23 the same approach has been applied by selecting PC as the final system, instead.

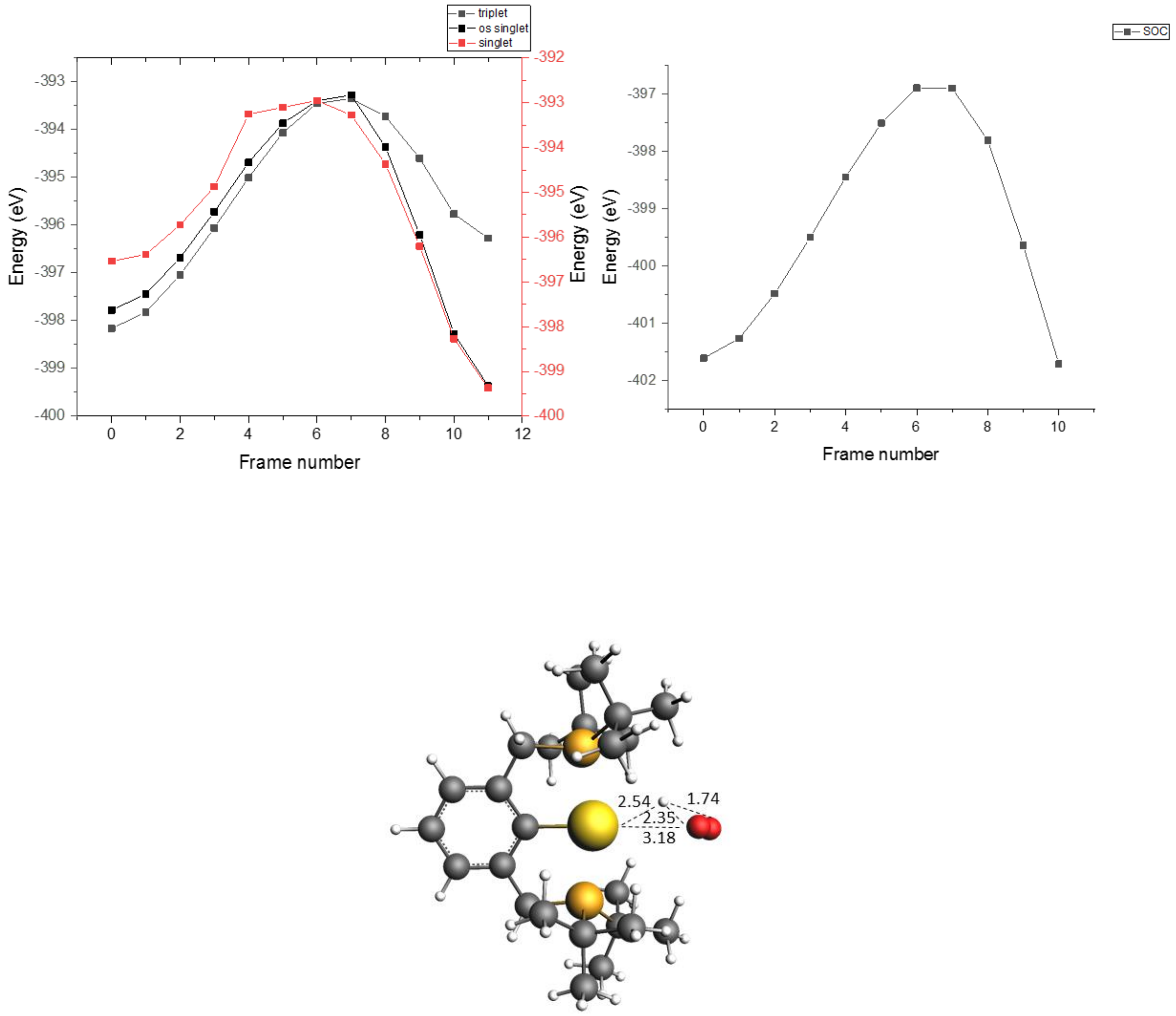

Figure S23: Reaction path computed with NEB using **RC** as the initial (frame number 0) and **PC** as the final (frame number 11) systems. For each image along the path a single point calculation has been performed in the triplet, singlet, open shell singlet spin states (left) and SOC state (right). Image 6 corresponding to both triplet/singlet crossing and SOC maximum energy is also shown (distances in Å).

From Figure S22 (left), we observe that the triplet path is lower than both the singlet and open shell singlet paths until frame number 7 is reached. However, the open shell singlet path reveals triplet spin contamination, corresponding to $\langle S^2 \rangle$ values ranging from 0.78 to 1.0 for images from 0 (RC) to 6. Moreover, along the singlet path images from 0 (RC) to 6 converge to a non-aufbau electronic structure. For images from 7 to 11 (INT) singlet and open shell singlet paths have exactly the same energy, with $\langle S^2 \rangle$ values equal to 0.00 and regular aufbau electronic structures, indicating a singlet pure spin state. The SOC path (Figure S22 right) shows a similar trend, with a maximum energy value calculated at frame number 7, where the triplet to singlet crossing occurs.

Analogous considerations apply for Figure S23, except that frame number 6 (instead of 7) represents in this case the triplet to singlet crossing point and the maximum SOC energy point.

NEB analysis suggests that the triplet/singlet crossing and TS SOC should be located at the same complex geometrical structure and that, as discussed above, more than a reaction coordinate is needed to describe the complete reaction path. From image 7 (Figure S22) and image 6 (Figure S23), we observe that the hydrogen abstraction should occur before (or concerted with) the OOH rebound which can take place through one oxygen or the other, leading to INT or PC, respectively. The NEB analysis is thus fully consistent with the above results.

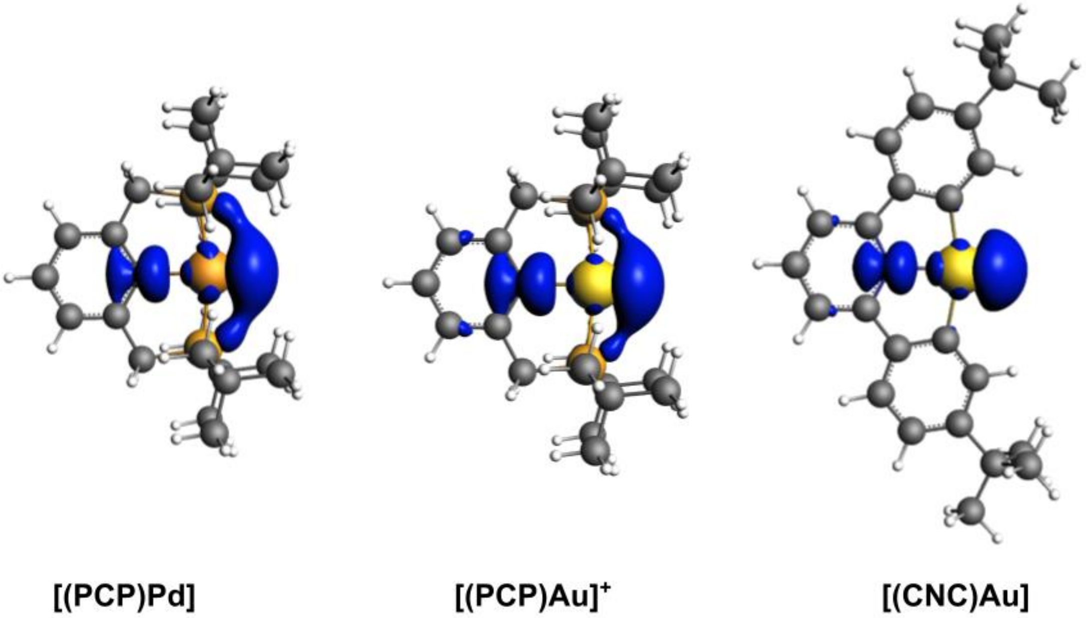

Figure S24. Spin densities of the [(^{tBu}PCP)Pd•] (left), [(^{tBu}PCP)Au•]$^+$ (center) and [(CNC)Au•] (right) open-shell metal fragments. The isosurface is set to 3 me/a_0^3.

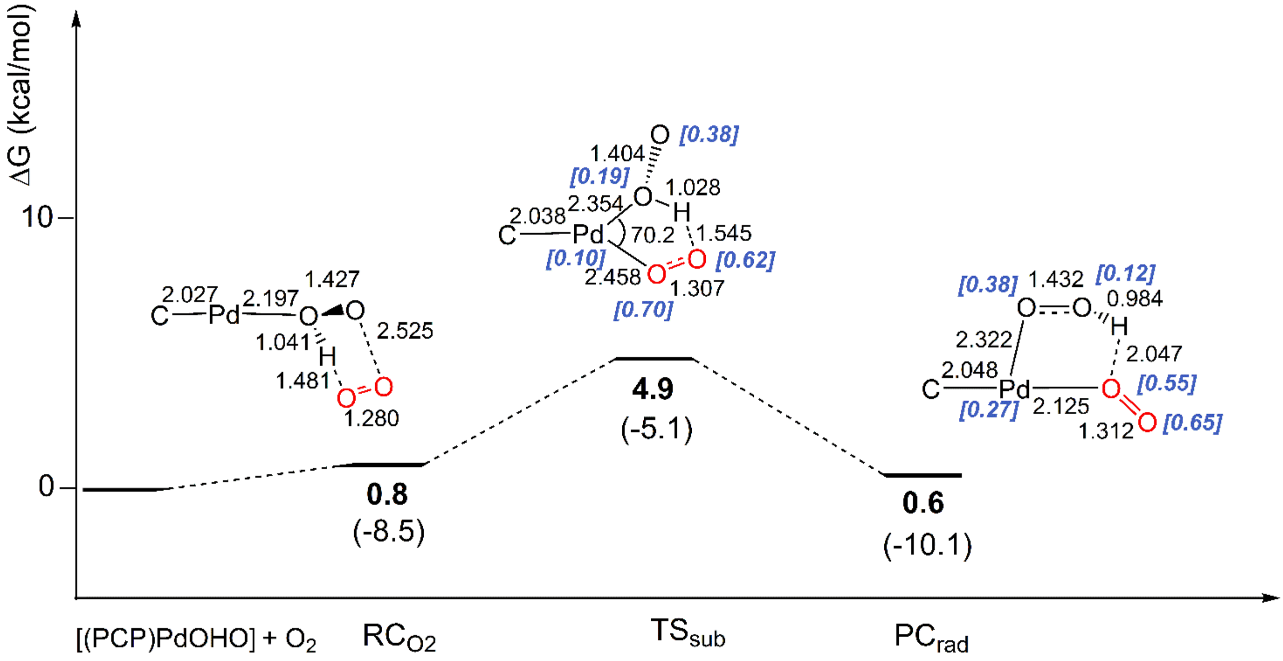

Figure S25. Reaction free energy profile (SOC) and schematic structures (with relevant geometrical parameters in Å and degrees) for the mechanism of the OOH substitution by O_2 from

INT structure with the model [(PCP)Pd-H] complex. ΔG values (in kcal/mol) refer to the energy of the isolated reactants ([(PCP)Pd-OHO] + O_2) in their ground spin state taken as zero. Values in parenthesis are the corresponding ΔE values (in kcal/mol). Values in square brackets (blue color) indicate unpaired α spin densities.

M-H (M = Au, Pd) bond analysis

**Complex**	[(^{tBu}PCP)PdH]		[(^{tBu}PCP)Au-H]$^+$		[(CNC)Au-H]	
**Fragments**	[(^{tBu}PCP)Pd]$^+$ - - - [H]$^-$	[(^{tBu}PCP)Pd] - - - [H]	[(^{tBu}PCPAu]$^{2+}$ - - - [H]$^-$	[(^{tBu}PCP)Au]$^+$ - - - [H]	[(CNC)Au]$^+$ - - - [H]$^-$	[(CNC)Au] - - - [H]
**ΔE_{Pauli}**	217.4	63.0	293.8	92.5	274.8	88.7
**ΔE_{elst}**	-291.0	-69.0	-423.8	-87.7	-366.0	-93.0
**ΔE_{oi}**	-92.7	-72.4	-132.2	-83.7	-91.2	-85.0
**ΔE**	-169.0	-80.2	-273.8	-81.4	-137.6	-90.7

Table S2. Comparative Energy Decomposition Analysis (EDA) of the interaction between [M] and [H] (M= (^{tBu}PCP)Pd, (^{tBu}PCP)Au, (CNC)Au)) fragments using different fragmentation schemes, i.e. singlet closed shell $[M]^{+/2+}$ and $[H]^-$ fragments or doublet open shell $[M]^{0/+}$ and [H] fragments. All energies are reported in kcal/mol.

	[(^{tBu}PCP)Pd-H]	[(^{tBu}PCP)Au-H]$^+$	[(CNC)Au-H]
ΔE_{oi} (kcal/mol)	-72.4	-83.7	-85.0
$\Delta E_{oi}{}^{1\alpha}$ (kcal/mol)	-49.7	-49.3	-49.0
$\Delta E_{oi}{}^{1\beta}$ (kcal/mol)	-17.4	-26.7	-30.2
$\lvert\nu_{1\alpha}\rvert$ (e)	0.52	0.51	0.46
$\lvert\nu_{1\beta}\rvert$ (e)	0.42	0.46	0.47
CT_{net} (e)	-0.12	-0.08	-0.06

Table S3. Comparative EDA and CD-NOCV (ETS-NOCV) results for the interaction between [M] and [H] (M= (^{tBu}PCP)Pd, (^{tBu}PCP)Au, (CNC)Au)) fragments using doublet open shell $[M]^{0/+}$ and [H] fragments. Eigenvalues ($\lvert\nu_k\rvert$) and orbital interaction energies ($\Delta E_{oi}{}^k$) associated to the first NOCV deformation density (k = 1) and to the corresponding α and β components. Total orbital interaction energies (ΔE_{oi}) and charge transfer (CT_{net}) are also reported.

Electronic structure of [(^{tBu}PCP)Au-H]$^+$, [(^{tBu}PCP)Pd-H], and [(CNC)Au-H]

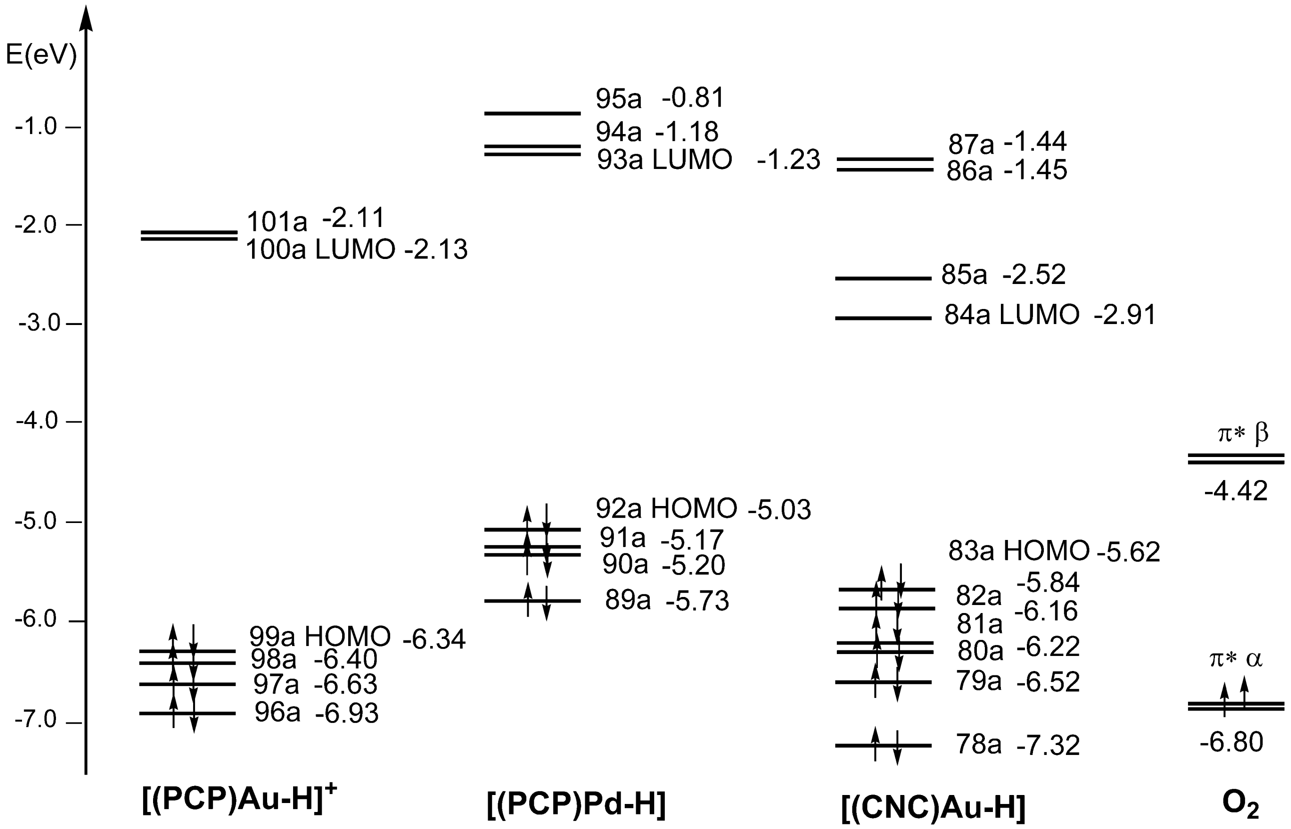

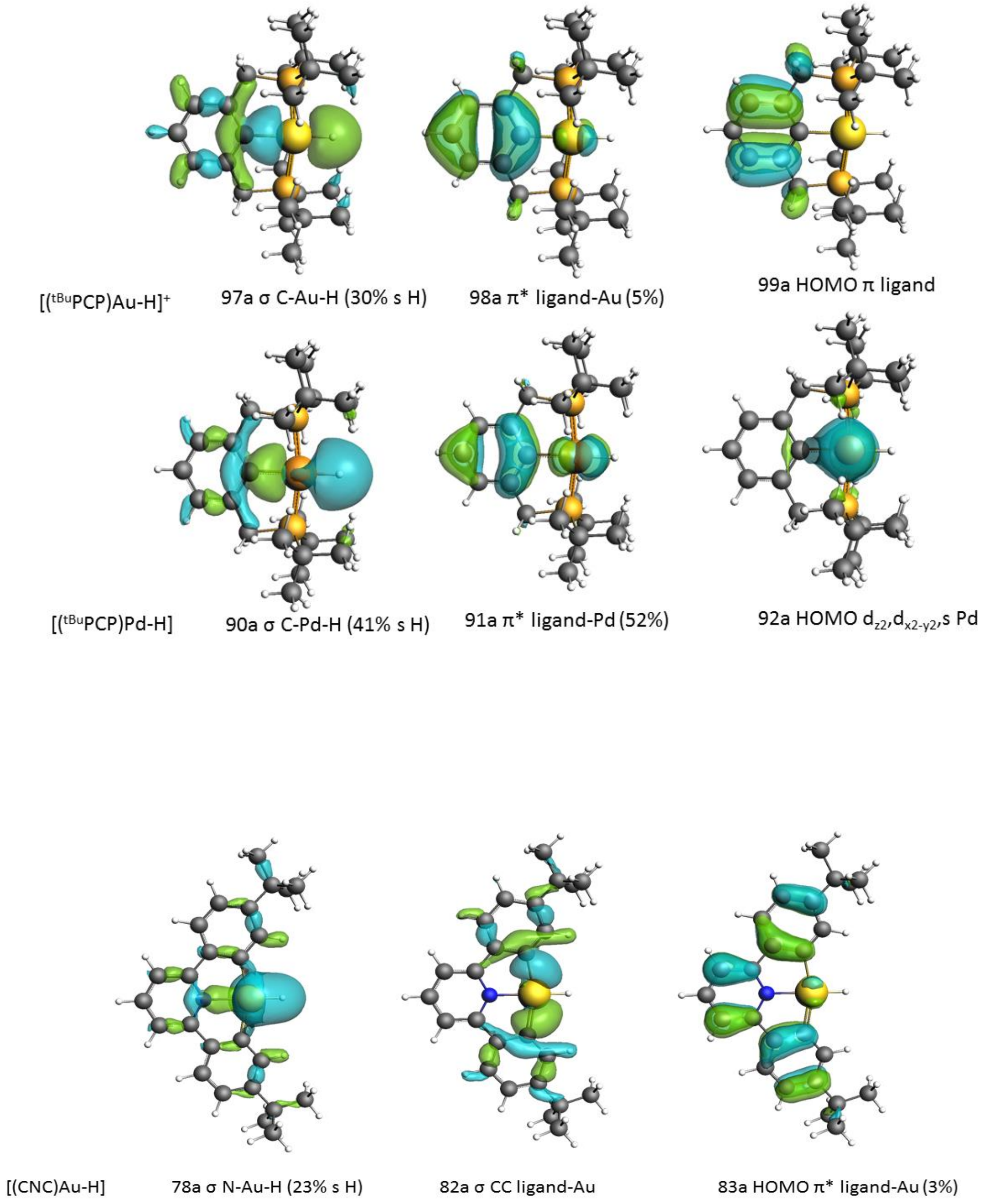

Figure S26. Ground state electronic structure of [(^{tBu}PCP)Au-H]$^+$, [(^{tBu}PCP)Pd-H], and [(CNC)Au-H] (singlet spin state) and O_2 (triplet spin state). Orbital energies are in eV. Relevant frontier molecular orbitals are visualized at a contour level of 0.03 a.u.

The ground state electronic structures of [(^{tBu}PCP)Au-H]$^+$, [(^{tBu}PCP)Pd-H], and [(CNC)Au-H] (singlet spin state) complexes and O_2 (triplet spin state) are reported in Figure S2. The most relevant molecular orbitals for describing the complex – O_2 interaction are HOMO-2 (97a MO for [(^{tBu}PCP)Au-H]$^+$ and 90a MO for [(^{tBu}PCP)Pd-H]) and HOMO-5 (78a MO for [(CNC)Au-H]), representing the σ M-H bonding orbital, and the doubly occupied π* antibonding orbital for O_2. Remarkably, the H 1s percentage contribution in these MOs varies significantly from 41% for [(^{tBu}PCP)Pd-H], to 30% for [(^{tBu}PCP)Au-H]$^+$, and 23% for [(CNC)Au-H]. In addition, a not negligible contribution from the ligand donor atom in trans position to H is found only for [(^{tBu}PCP)Au-H]$^+$ and [(^{tBu}PCP)Pd-H] (26% and 23% C $2p_z$, respectively). Instead, for [(CNC)Au-H], the ligand contribution is very small (4% N $2p_z$), and, correspondingly, a larger metal involvement is observed (Au: 18% 6s + 18% 5d) with respect to [(^{tBu}PCP)Au-H]$^+$ and [(^{tBu}PCP)Pd-H] (14% Au $6p_z$ and 12% Pd $5p_z$, respectively). These relevant differences in the nature of the σ M-H bonding orbitals do have an impact on the reactivity with dioxygen.

References

[1] M. Mitoraj, A. Michalak, *J. Mol. Model.* **2007**, *13*, 347–355.

[2] A. Michalak, M. Mitoraj, T. Ziegler, *J. Phys. Chem. A* **2008**, *112*, 1933–1939.

[3] R. F. Nalewajski, J. ozek, *Int. J. Quantum Chem.* **1994**, *51*, 187–200.

[4] R. F. Nalewajski, J. Mrozek, A. Michalak, *Int. J. Quantum Chem.* **1997**, *61*, 589–601.

[5] T. Lu, F. Chen, *J. Phys. Chem. A* **2013**, *117*, 3100–3108.

[6] L. Belpassi, I. Infante, F. Tarantelli, L. Visscher, *J. Am. Chem. Soc.* **2008**, *130*, 1048–1060.

[7] G. Bistoni, S. Rampino, F. Tarantelli, L. Belpassi, *J. Chem. Phys.* **2015**, *142*, 084112.

[8] N. Salvi, L. Belpassi, F. Tarantelli, *Chem. - A Eur. J.* **2010**, *16*, 7231–7240.

[9] F. M. Bickelhaupt, E. J. Baerends, in *Rev. Comput. Chem.*, Wiley-VCH Verlag, **2007**, pp. 1–86.

[10] K. Morokuma, *J. Chem. Phys.* **1971**, *55*, 1236–1244.

[11] L. Zhao, M. von Hopffgarten, D. M. Andrada, G. Frenking, *Wiley Interdiscip. Rev. Comput. Mol. Sci.* **2018**, *8*, 1345.

[12] M. P. Mitoraj, A. Michalak, T. Ziegler, *J. Chem. Theory Comput.* **2009**, *5*, 962–975.

[13] A.S. Phearman, Y. Ardon, K.I. Goldberg, *J. Am. Chem. Soc.* **2024**, *146*, 4045-4059.

[14] A.A. Ovchinnikov, J.K. Labanowski, *Phys. Rev. A* **1996**, *53*, 3946-3952.

[15] S. Chowdhury, I. Rivalta, N. Russo, E. Sicilia, *Chem. Phys. Lett.* **2007**, *443*, 183-189.

[16] G. Henkelman, B.P. Uberuaga, H. Jonsson, *J. Chem. Phys.* **2000**, *113*, 9901-9904.

Atomic displacements for the imaginary frequency of relevant transition states

[^{tBu}PCP-Au-H]+ TSI triplet

-95.813

1.C	-0.002	-0.001	-0.004
2.C	0.000	0.003	0.014
3.C	0.000	0.006	0.034
4.C	-0.001	0.004	0.013
5.C	-0.001	0.000	-0.005
6.C	-0.002	-0.001	-0.009
7.C	-0.000	-0.002	0.005
8.P	0.004	0.000	-0.018
9.C	0.022	-0.019	-0.015
10.C	0.061	-0.053	-0.035
11.AU	0.003	0.001	-0.012
12.P	0.002	0.001	-0.010
13.C	0.013	0.008	-0.007
14.C	0.011	0.009	-0.005
15.C	0.001	0.001	0.002
16.C	-0.003	-0.001	-0.006
17.C	-0.002	-0.006	0.002
18.C	-0.005	-0.002	-0.005
19.C	-0.011	-0.003	-0.011
20.C	-0.013	0.012	-0.013
21.C	-0.014	0.014	-0.003

22.C	-0.016	0.015	-0.025
23.C	-0.014	0.016	-0.009
24.C	0.024	-0.015	-0.033
25.C	0.027	-0.024	0.023
26.C	0.014	0.009	-0.013
27.C	0.013	0.009	-0.005
28.O	0.010	-0.277	0.135
**29.O**	**-0.131**	**0.326**	**0.142**
**30.H**	**-0.320**	**-0.556**	**0.513**
31.H	-0.002	-0.001	-0.018
32.H	-0.002	-0.004	-0.023
33.H	-0.002	-0.003	-0.016
34.H	-0.001	0.006	0.012
35.H	0.000	-0.018	0.007
36.H	-0.002	0.007	0.001
37.H	0.006	-0.007	0.002
38.H	0.022	0.009	-0.011
39.H	0.012	0.013	-0.022
40.H	0.007	0.007	-0.010
41.H	0.017	0.012	-0.004
42.H	0.011	0.007	-0.005
43.H	0.011	0.008	0.000
44.H	0.008	0.011	-0.006
45.H	0.012	0.009	-0.003
46.H	0.011	0.009	-0.000

47.H	-0.002	-0.001	-0.007
48.H	-0.009	-0.004	-0.008
49.H	-0.008	-0.001	0.001
50.H	-0.021	-0.012	-0.009
51.H	-0.015	0.006	-0.025
52.H	-0.004	-0.003	-0.006
53.H	-0.007	-0.006	0.006
54.H	0.000	-0.005	-0.001
55.H	0.003	-0.010	0.005
56.H	-0.019	0.023	-0.025
57.H	-0.014	0.011	-0.030
58.H	-0.015	0.015	-0.028
59.H	-0.024	0.016	-0.001
60.H	-0.010	0.013	0.003
61.H	-0.010	0.013	0.001
62.H	-0.016	0.024	-0.010
63.H	-0.014	0.013	-0.009
64.H	-0.012	0.015	-0.001
65.H	0.038	-0.040	0.020
66.H	0.013	-0.005	0.030
67.H	0.032	-0.029	0.043
68.H	0.039	-0.028	-0.034
69.H	0.027	0.002	-0.054
70.H	0.013	-0.012	-0.025
71.H	0.078	-0.059	-0.028

72.H	0.089	-0.061	-0.017
73.H	0.052	-0.063	-0.093

$[PCP\text{-}Au\text{-}H]^+$ model TSI SOC

-703.63

1.C	0.008	-0.001	-0.000
2.C	0.009	0.008	0.003
3.C	-0.007	-0.002	0.015
4.C	0.010	0.000	0.005
5.C	0.004	0.005	-0.003
6.C	0.004	0.002	0.001
7.C	0.001	0.002	0.000
8.P	-0.008	0.006	-0.001
9.C	0.015	-0.009	0.006
10.AU	-0.001	-0.001	-0.003
11.P	0.001	-0.009	-0.002
12.C	0.000	0.006	0.001
13.C	0.002	-0.000	0.003
14.C	-0.001	0.001	-0.001
15.C	0.003	-0.002	0.001
16.O	-0.006	-0.047	-0.053
17.O	-0.018	0.062	0.017
**18.H**	**0.177**	**-0.174**	**0.960**
19.H	0.017	0.005	-0.006
20.H	0.004	0.003	0.003
21.H	0.016	0.010	-0.001

22.H -0.000 -0.003 0.002

23.H -0.002 -0.004 -0.001

24.H -0.001 0.000 0.003

25.H -0.001 0.002 -0.001

26.H -0.006 0.005 -0.008

27.H -0.000 0.001 0.002

28.H 0.003 0.000 -0.001

29.H 0.001 0.001 0.001

30.H -0.000 0.002 0.001

31.H -0.000 -0.001 -0.003

32.H 0.035 -0.026 0.008

33.H 0.029 0.006 0.040

34.H -0.008 -0.000 -0.000

35.H 0.005 0.001 -0.005

36.H 0.014 -0.004 -0.014

37.H 0.001 -0.000 0.002

[CNC’-Au-H] model TSI SOC

-494.082

1.C -0.000 -0.001 -0.001

2.C -0.002 -0.002 0.000

3.C -0.001 -0.003 0.001

4.C 0.000 -0.000 0.000

5.C 0.000 0.000 -0.000

6.C -0.000 -0.000 -0.000

7.AU	0.001	-0.000	-0.004
8.N	0.000	-0.005	0.013
9.C	0.004	-0.006	0.003
10.C	-0.002	-0.005	-0.001
11.C	-0.001	-0.004	0.000
12.C	0.002	-0.007	-0.001
13.C	-0.003	-0.008	0.003
14.C	0.002	0.001	0.001
15.C	0.003	0.003	-0.001
16.C	-0.007	-0.004	0.000
17.C	-0.003	-0.003	0.001
18.C	-0.001	-0.002	-0.000
19.C	0.002	0.000	0.000
20.C	-0.001	-0.001	-0.000
21.C	-0.000	-0.000	-0.001
22.O	-0.025	0.099	-0.022
23.O	0.040	-0.019	0.008
**24.H**	**-0.282**	**-0.602**	**0.737**
25.H	-0.001	-0.011	-0.003
26.H	-0.001	-0.004	0.000
27.H	0.000	-0.009	-0.005
28.H	0.001	-0.001	-0.000
29.H	0.001	0.001	-0.001
30.H	-0.004	-0.002	-0.004
31.H	-0.023	-0.007	0.003

32.H -0.000 -0.005 -0.001

33.H 0.003 0.000 0.002

34.H -0.003 0.002 -0.000

35.H -0.000 -0.002 -0.001

36.H -0.001 -0.000 -0.001

37.H -0.000 -0.000 -0.001

38.H -0.000 0.000 -0.001

39.H 0.000 0.000 -0.001

[PCP-Pd-H] model TSI SOC

-162.265

1.C 0.032 0.029 0.013

2.C 0.037 0.020 0.001

3.C 0.027 0.016 -0.011

4.C 0.030 0.022 -0.004

5.C 0.026 0.030 0.011

6.C 0.022 0.028 0.004

7.C 0.040 0.014 -0.003

8.P 0.029 0.019 0.026

9.C -0.019 -0.000 -0.009

10.PD 0.022 0.018 0.008

11.P 0.024 0.004 0.011

12.C -0.006 -0.042 0.016

13.C 0.026 0.016 -0.039

14.C	0.073	0.070	0.034
15.C	-0.044	0.044	0.015
**16.O**	**-0.304**	**-0.254**	**-0.087**
**17.O**	**-0.149**	**-0.134**	**-0.066**
**18.H**	**0.166**	**0.209**	**0.157**
19.H	0.025	0.029	-0.016
20.H	0.015	0.033	-0.010
21.H	0.034	0.030	0.029
22.H	0.027	0.019	-0.021
23.H	0.048	0.007	-0.005
24.H	0.005	-0.038	-0.110
25.H	0.027	0.073	-0.058
26.H	-0.021	0.022	0.020
27.H	-0.023	-0.019	-0.029
28.H	0.033	-0.064	0.021
29.H	-0.012	-0.030	-0.008
30.H	-0.093	0.015	0.016
31.H	0.017	0.047	0.047
32.H	0.023	0.099	0.065
33.H	-0.001	0.018	0.023
34.H	0.084	0.032	0.031
35.H	-0.077	-0.059	0.017
36.H	0.008	0.013	0.014
37.H	0.033	-0.097	0.028

xyz geometries

[(PCP)Au-H]+

71

AU	-0.002608	-0.001168	0.167896
H	-0.031488	-0.000976	1.800851
C	0.034189	-0.000794	-1.920977
C	-1.172566	-0.000088	-2.651843
C	-1.137747	0.000040	-4.051993
C	0.084107	-0.000230	-4.725003
C	1.281326	-0.000648	-4.009083
C	1.266061	-0.000849	-2.608667
C	-2.510596	0.000916	-1.947279
H	-2.069412	0.000435	-4.618556
H	0.103701	-0.000047	-5.814371
H	2.232576	-0.000757	-4.542120
C	2.578292	-0.001029	-1.857516
P	2.322573	-0.000408	-0.024704
H	3.183595	-0.876873	-2.126411
H	3.184523	0.873935	-2.127194
P	-2.319783	-0.000119	-0.106466
H	-3.107501	-0.873679	-2.238105
H	-3.105662	0.877073	-2.237238
C	-3.074908	-1.598893	0.504157
C	-3.073509	1.598850	0.505129
C	-2.231911	2.707560	-0.157371
C	-4.545502	1.739109	0.092451
C	-2.932744	1.708599	2.030500
C	-4.547058	-1.737764	0.091456
C	-2.234342	-2.707935	-0.159087
C	-2.934350	-1.710018	2.029520
H	-3.205260	-2.731630	2.328831
H	-1.905034	-1.518849	2.356604
H	-3.603212	-1.020904	2.554650
H	-4.885454	-2.750225	0.351819
H	-5.188344	-1.026396	0.621512
H	-4.697806	-1.607656	-0.987153
H	-2.635785	-3.681287	0.153151
H	-2.265148	-2.667130	-1.254359
H	-1.184730	-2.656639	0.158894
H	-3.203074	2.730107	2.330711
H	-3.602091	1.019407	2.554627
H	-1.903617	1.516493	2.357663
H	-2.632409	3.681082	0.155549
H	-1.182338	2.655027	0.160584
H	-2.262796	2.667589	-1.252677
H	-4.883101	2.751628	0.353646
H	-4.696281	1.609952	-0.986287
H	-5.187267	1.027820	0.622032
C	3.054473	1.599153	0.611597
C	3.055942	-1.598949	0.612389
C	2.237311	-2.707701	-0.078028
C	4.540880	-1.738410	0.248917
C	2.864352	-1.708817	2.132190
C	4.539213	1.739792	0.247821
C	2.235003	2.706877	-0.079527

C	2.862529	1.709779	2.131363
H	3.124896	-2.730203	2.441150
H	3.515639	-1.019537	2.678599
H	1.824787	-1.516951	2.424577
H	4.870284	-2.750931	0.520408
H	4.727614	-1.608389	-0.824035
H	5.164227	-1.027099	0.800098
H	2.625929	-3.681234	0.249562
H	1.177396	-2.653896	0.203138
H	2.306546	-2.668804	-1.171614
H	4.867507	2.752879	0.518567
H	5.163480	1.029669	0.799332
H	4.725838	1.609201	-0.825061
H	3.121562	2.731750	2.439694
H	1.823222	1.516681	2.423790
H	3.514629	1.021781	2.678420
H	2.622973	3.680919	0.247312
H	2.304085	2.667299	-1.173093
H	1.175160	2.652510	0.201826

O2 triplet

2

O	0.000000	0.000000	-0.041202
O	0.000000	0.000000	1.181202

[(PCP)Au-H]+ - O2 RC triplet

73

C	1.393831	-3.926050	0.258006
C	1.329400	-2.544364	0.043382
C	0.103353	-1.870332	0.207255
C	-1.050323	-2.587220	0.580581
C	-0.973023	-3.970969	0.776603
C	0.245663	-4.633195	0.618353
C	2.556048	-1.781800	-0.401219
P	2.317876	0.034981	-0.157299
C	3.104615	0.875852	-1.628403
C	2.171863	0.571496	-2.816680
AU	-0.006129	0.194811	-0.113703
P	-2.301720	-0.180100	0.024671
C	-3.181126	0.980819	1.195659
C	-2.225667	1.152019	2.392597
C	-2.352386	-1.854103	0.807156
C	-3.033216	-0.342269	-1.690157
C	-4.552911	-0.547840	-1.637247
C	-2.681662	0.904989	-2.517002
C	-2.360859	-1.568781	-2.335754
C	3.021280	0.471108	1.521325
C	4.554474	0.419459	1.520409
C	2.524053	1.865237	1.936284
C	2.462736	-0.567902	2.511761
C	3.153839	2.392518	-1.402606
C	4.508920	0.322431	-1.917564
C	-3.394705	2.342579	0.522006
C	-4.517605	0.397083	1.680214
O	0.041432	4.326594	0.424672
O	-0.176250	4.316460	1.628435

H	-0.091482	1.805534	-0.371182
H	-1.863617	-4.531797	1.061302
H	0.301100	-5.709664	0.777565
H	2.340658	-4.452753	0.135877
H	3.469094	-2.122260	0.104595
H	2.723132	-1.934677	-1.477268
H	-2.507511	-1.691578	1.883645
H	-3.222709	-2.421048	0.451488
H	-3.746343	3.056360	1.279371
H	-2.461747	2.731669	0.095986
H	-4.152573	2.295531	-0.267850
H	-4.955063	1.096075	2.406380
H	-5.236242	0.268686	0.865214
H	-4.389158	-0.566511	2.187125
H	-2.708548	1.801442	3.135219
H	-1.992935	0.199559	2.885248
H	-1.284674	1.625618	2.085484
H	-3.049757	0.757680	-3.541399
H	-3.141550	1.815334	-2.120835
H	-1.596486	1.059900	-2.561778
H	-2.726734	-1.652511	-3.368031
H	-1.269182	-1.465793	-2.367595
H	-2.602625	-2.502439	-1.815503
H	-4.910749	-0.760176	-2.654231
H	-4.836417	-1.396633	-1.002375
H	-5.074925	0.347683	-1.282659
H	2.882431	2.074012	2.953374
H	2.894495	2.656936	1.278314
H	1.428174	1.908941	1.944050
H	4.908087	0.558920	2.551367
H	4.938340	-0.545538	1.166365
H	4.990368	1.217825	0.909886
H	2.812554	-0.300390	3.518000
H	1.365763	-0.573813	2.521384
H	2.809430	-1.584262	2.293478
H	4.889602	0.809651	-2.825787
H	5.213977	0.529170	-1.106945
H	4.498540	-0.758017	-2.103326
H	3.457493	2.875905	-2.341082
H	2.170937	2.789551	-1.119372
H	3.884503	2.669669	-0.634585
H	2.608172	1.010987	-3.723949
H	2.052524	-0.504932	-2.993749
H	1.179044	1.014110	-2.666091

[(PCP)Au-H]+ - O2 RC open shell singlet

73

C	1.395574	-3.925538	0.231859
C	1.330516	-2.542159	0.027975
C	0.106035	-1.870025	0.205765
C	-1.045849	-2.587034	0.583346
C	-0.967610	-3.972364	0.768115
C	0.249154	-4.634200	0.594695
C	2.554543	-1.776110	-0.418838
P	2.319826	0.038540	-0.154194
C	3.106429	0.895560	-1.616165
C	2.167495	0.615087	-2.805682
AU	-0.004325	0.197880	-0.102509

P	-2.299220	-0.179049	0.040445
C	-3.183323	0.983228	1.207001
C	-2.228222	1.166299	2.402911
C	-2.344210	-1.852531	0.826086
C	-3.031605	-0.345697	-1.674900
C	-4.552270	-0.545055	-1.620348
C	-2.675252	0.898858	-2.504310
C	-2.365015	-1.576165	-2.318710
C	3.022362	0.456917	1.530358
C	4.555624	0.403001	1.529326
C	2.526740	1.847579	1.959301
C	2.462309	-0.591130	2.510775
C	3.167707	2.408435	-1.368000
C	4.505733	0.335243	-1.917842
C	-3.406594	2.340493	0.526908
C	-4.515893	0.393015	1.695013
O	-0.029223	4.205366	0.208568
O	-0.034324	4.404245	1.419165
H	-0.090604	1.811350	-0.346984
H	-1.856379	-4.534841	1.055196
H	0.304770	-5.711942	0.744579
H	2.341037	-4.451950	0.098991
H	3.471214	-2.123109	0.075802
H	2.711958	-1.916932	-1.497835
H	-2.484911	-1.688256	1.904192
H	-3.219757	-2.418320	0.481719
H	-3.766643	3.053744	1.280740
H	-2.476179	2.736937	0.102039
H	-4.161974	2.283891	-0.264657
H	-4.957910	1.093027	2.417461
H	-5.233519	0.255310	0.880753
H	-4.380682	-0.566902	2.207102
H	-2.716433	1.813451	3.144017
H	-1.987454	0.217186	2.898210
H	-1.291410	1.647027	2.094052
H	-3.049249	0.753696	-3.526883
H	-3.126127	1.812814	-2.106218
H	-1.589208	1.045322	-2.554641
H	-2.733678	-1.661144	-3.349832
H	-1.273165	-1.477146	-2.353620
H	-2.608868	-2.507553	-1.795497
H	-4.911546	-0.757800	-2.636691
H	-4.838379	-1.391731	-0.983912
H	-5.070759	0.352883	-1.266826
H	2.882416	2.044071	2.979697
H	2.900899	2.645807	1.311512
H	1.430751	1.894259	1.964704
H	4.909142	0.535555	2.561183
H	4.937373	-0.560672	1.169587
H	4.992927	1.204092	0.923468
H	2.813879	-0.334528	3.519153
H	1.365407	-0.594019	2.521909
H	2.805711	-1.606104	2.281509
H	4.889834	0.836030	-2.817211
H	5.213354	0.520094	-1.104141
H	4.484840	-0.741356	-2.124044
H	3.468327	2.903753	-2.301166
H	2.189601	2.808149	-1.072455
H	3.904986	2.669107	-0.600631
H	2.605871	1.060723	-3.708877

H	2.036791	-0.457850	-2.994821
H	1.179821	1.066094	-2.646847

[(PCP)Au-H]+ - O2 TSI open shell singlet H-abstraction

73

C	1.340181	-2.535994	0.022032
C	0.108233	-1.895493	0.202226
C	-1.055605	-2.582429	0.568640
C	-0.973999	-3.970434	0.741205
C	0.244253	-4.628707	0.568439
C	1.395335	-3.922351	0.218058
AU	-0.000073	0.193027	-0.073181
P	-2.311186	-0.155878	0.057412
C	-3.031251	-0.292664	-1.662172
C	-2.666724	0.970362	-2.459586
C	-2.347439	-1.840792	0.817714
C	2.561076	-1.760607	-0.410551
P	2.337365	0.054630	-0.135190
C	3.031148	0.467890	1.550353
C	2.530232	1.861248	1.964661
C	3.134510	0.906547	-1.595336
C	3.242066	2.415692	-1.339815
C	-3.204085	0.979538	1.243308
C	-3.492563	2.329851	0.573099
C	-4.506499	0.344423	1.754748
C	-2.226722	1.190939	2.415843
C	-2.359704	-1.509107	-2.326024
C	-4.551685	-0.493649	-1.618004
C	4.517646	0.312907	-1.905718
C	2.180302	0.657339	-2.778894
C	2.459536	-0.576604	2.526556
C	4.563646	0.410995	1.558936
H	-0.151803	1.983720	-0.227800
H	-1.865676	-4.532378	1.019332
H	0.297686	-5.707227	0.711088
H	2.342139	-4.447144	0.089745
H	3.474244	-2.114722	0.084698
H	2.720304	-1.895294	-1.489930
H	-2.481072	-1.690137	1.898635
H	-3.223976	-2.402739	0.470783
H	-3.870546	3.021089	1.338528
H	-2.589389	2.773982	0.139071
H	-4.258482	2.246334	-0.205679
H	-4.966642	1.038932	2.471086
H	-5.227445	0.168173	0.950367
H	-4.331006	-0.601290	2.280385
H	-2.727918	1.793971	3.184991
H	-1.917884	0.247185	2.883592
H	-1.328988	1.735830	2.093556
H	-3.029012	0.849292	-3.489286
H	-3.119953	1.876221	-2.046437
H	-1.578944	1.115262	-2.499936
H	-2.723458	-1.575062	-3.360214
H	-1.267649	-1.407713	-2.356969
H	-2.604552	-2.450968	-1.822328
H	-4.908367	-0.672543	-2.641684
H	-4.837583	-1.362025	-1.011368

H	-5.072326	0.390885	-1.235239
H	2.870132	2.063620	2.989287
H	2.912890	2.656344	1.318365
H	1.432991	1.912916	1.957355
H	4.911478	0.553409	2.591363
H	4.945405	-0.557387	1.211834
H	5.006648	1.204657	0.947449
H	2.804896	-0.321749	3.537433
H	1.362171	-0.573243	2.532786
H	2.799999	-1.593491	2.300834
H	4.913775	0.819958	-2.796343
H	5.229768	0.467086	-1.089305
H	4.471232	-0.758706	-2.131178
H	3.568131	2.900716	-2.269947
H	2.277401	2.851947	-1.053002
H	3.982953	2.649141	-0.567232
H	2.622783	1.095110	-3.683773
H	2.017397	-0.410605	-2.972502
H	1.205679	1.135116	-2.613433
O	-0.095685	3.227215	-0.363762
O	-0.109746	3.851535	0.782349

[(PCP)Au-H]+ - O2 TSI triplet H-abstraction

73

C	1.215415	-3.958843	0.215693
C	1.224994	-2.586771	-0.071299
C	-0.005162	-1.933548	-0.169230
C	-1.232944	-2.585922	-0.044709
C	-1.216922	-3.957982	0.243879
C	0.000682	-4.629032	0.382885
C	2.513505	-1.833054	-0.321722
P	2.320168	-0.013694	-0.014409
C	3.332418	0.841297	-1.337138
C	2.517683	0.680394	-2.633346
AU	-0.005541	0.221012	-0.171741
P	-2.334835	-0.007874	-0.014363
C	-2.886815	0.326530	1.747859
C	-2.160083	-0.706808	2.628250
C	-2.526915	-1.835631	-0.275012
C	-3.365272	0.803312	-1.350730
C	-4.746652	0.149581	-1.509337
C	-3.511398	2.300983	-1.050297
C	-2.554914	0.618713	-2.647796
C	2.885601	0.279898	1.750313
C	4.405430	0.139438	1.902488
C	2.418811	1.672332	2.204446
C	2.174751	-0.782312	2.609387
C	3.461768	2.331001	-0.990904
C	4.719837	0.210468	-1.530576
C	-2.425543	1.733863	2.159844
C	-4.403889	0.180245	1.919641
O	0.155354	3.612331	-2.006922
O	-0.188833	3.517559	-0.714566
H	0.620643	2.759629	-2.215729
H	-2.155774	-4.500308	0.362510
H	0.003063	-5.692616	0.619295
H	2.156437	-4.501610	0.312635
H	3.350556	-2.233866	0.263649

H	2.793722	-1.941648	-1.379365
H	-3.347912	-2.224800	0.340279
H	-2.834986	-1.968485	-1.321981
H	-2.661291	1.884123	3.222325
H	-1.341253	1.848649	2.029332
H	-2.923333	2.522837	1.587892
H	-4.646501	0.272832	2.987614
H	-4.953836	0.963470	1.386570
H	-4.767638	-0.798217	1.581718
H	-2.392381	-0.489138	3.679403
H	-2.474228	-1.735245	2.418660
H	-1.071188	-0.649114	2.504522
H	-3.951884	2.795074	-1.927116
H	-4.174252	2.483879	-0.197169
H	-2.540762	2.772458	-0.848727
H	-3.116614	1.060589	-3.481894
H	-1.582067	1.123582	-2.585062
H	-2.378716	-0.438153	-2.885332
H	-5.270514	0.647620	-2.337236
H	-4.675641	-0.915456	-1.759245
H	-5.363698	0.255708	-0.612002
H	2.655933	1.791816	3.270445
H	2.912868	2.480268	1.656286
H	1.333879	1.786676	2.079412
H	4.658353	0.206332	2.969949
H	4.772013	-0.827497	1.535830
H	4.944953	0.939955	1.384610
H	2.416451	-0.590682	3.663402
H	1.084390	-0.730468	2.498003
H	2.494821	-1.802172	2.369438
H	5.227063	0.732096	-2.354140
H	5.346340	0.305745	-0.638814
H	4.657350	-0.849923	-1.801332
H	3.861478	2.867452	-1.861875
H	2.493947	2.777312	-0.726873
H	4.149380	2.495381	-0.154088
H	3.024794	1.211656	-3.449093
H	2.402133	-0.369165	-2.930836
H	1.501598	1.094184	-2.527363

[(PCP)Au-H]+ - O2 INT open shell singlet H-abstraction

73

C	1.365517	-2.481727	0.053110
C	0.135527	-1.829491	0.253855
C	-1.018291	-2.547811	0.616030
C	-0.922520	-3.933271	0.788184
C	0.296760	-4.585162	0.614855
C	1.432761	-3.865676	0.250021
AU	0.007847	0.183313	-0.079524
P	-2.320156	-0.177573	0.077365
C	-3.034109	-0.302864	-1.644509
C	-2.610026	0.942508	-2.441995
C	-2.332532	-1.849032	0.843957
C	2.583737	-1.726484	-0.410309
P	2.375058	0.077803	-0.124133
C	3.078389	0.469016	1.563172
C	2.705591	1.907920	1.956877

C	3.149856	0.951295	-1.581928
C	3.266333	2.456266	-1.300752
C	-3.197665	0.953398	1.279335
C	-3.424619	2.339334	0.660192
C	-4.530497	0.336198	1.732021
C	-2.233677	1.090181	2.475417
C	-2.404001	-1.545901	-2.297253
C	-4.561539	-0.440111	-1.613372
C	4.526819	0.355589	-1.915647
C	2.183546	0.719926	-2.759509
C	2.408580	-0.511069	2.544689
C	4.600238	0.271207	1.581702
H	-0.937536	2.479871	-0.962487
H	-1.812159	-4.498983	1.063383
H	0.360682	-5.662606	0.759206
H	2.383367	-4.378415	0.105886
H	3.502339	-2.092801	0.064804
H	2.714902	-1.864121	-1.493139
H	-2.494793	-1.698807	1.920508
H	-3.183388	-2.437575	0.478540
H	-3.921974	2.968293	1.411812
H	-2.467326	2.825885	0.424428
H	-4.074417	2.307753	-0.220865
H	-4.960859	0.995459	2.498590
H	-5.254125	0.256385	0.915191
H	-4.402724	-0.653522	2.185689
H	-2.731690	1.691798	3.248613
H	-1.978826	0.122996	2.927143
H	-1.316162	1.614179	2.170533
H	-3.039854	0.880356	-3.450400
H	-2.963378	1.877375	-1.992916
H	-1.518234	0.983994	-2.559562
H	-2.745977	-1.593343	-3.339780
H	-1.307902	-1.494709	-2.304296
H	-2.704633	-2.475884	-1.802083
H	-4.914331	-0.620471	-2.638021
H	-4.886910	-1.286463	-0.995957
H	-5.048485	0.470973	-1.249354
H	2.905646	2.031289	3.030256
H	3.309254	2.645129	1.418595
H	1.646076	2.137906	1.765798
H	4.952398	0.407788	2.613632
H	4.899091	-0.734879	1.261089
H	5.114210	1.009456	0.956636
H	2.799524	-0.300421	3.548994
H	1.319620	-0.380459	2.572218
H	2.625123	-1.559828	2.311917
H	4.919531	0.881781	-2.796777
H	5.245898	0.485512	-1.100995
H	4.471226	-0.710069	-2.166485
H	3.544377	2.959978	-2.236593
H	2.319486	2.883444	-0.950992
H	4.048041	2.670594	-0.564066
H	2.636114	1.138215	-3.668599
H	1.991298	-0.344452	-2.946278
H	1.225917	1.229303	-2.596660
O	-0.076834	2.300179	-0.529279
O	-0.179709	3.057092	0.734747

[(PCP)Au-H]+ - O2 PC triplet H-abstraction

73

C	1.338330	-3.893358	0.149664
C	1.246559	-2.523297	-0.090696
C	0.036033	-1.850432	0.105237
C	-1.088522	-2.570611	0.553108
C	-0.989164	-3.950711	0.819791
C	0.227980	-4.604310	0.603310
C	-0.064544	-0.371457	-0.168168
P	-1.505790	0.333678	0.727591
C	-0.959100	0.825931	2.448139
C	-0.117966	-0.334795	3.008278
AU	-2.869488	-1.592174	0.874449
P	-3.717718	-3.795496	0.950822
C	-4.278300	-4.350897	-0.747831
C	-3.029468	-4.467696	-1.638204
C	-2.176743	-4.706444	1.360852
C	-2.221965	1.684389	-0.341783
C	-1.110026	2.618867	-0.846912
C	-2.885243	0.968391	-1.534098
C	-3.287192	2.464798	0.438882
C	-4.923967	-4.037273	2.355008
C	-5.016859	-5.522821	2.742057
C	-4.361604	-3.228264	3.539936
C	-6.297248	-3.476107	1.963480
C	-4.986053	-5.710877	-0.665331
C	-5.209363	-3.286498	-1.346291
C	-0.112451	2.106379	2.400825
C	-2.195363	1.021768	3.340092
O	-5.979445	-0.234943	-0.049582
H	-5.584829	-0.879875	-0.667694
H	0.305356	-5.673689	0.797005
H	2.283543	-4.409438	-0.011964
H	2.119780	-1.968598	-0.432794
H	0.855658	0.164247	0.097682
H	-0.239547	-0.189391	-1.238249
H	-2.124227	-4.763757	2.457764
H	-2.224105	-5.738310	0.990920
H	-6.946008	-3.503106	2.849566
H	-6.226269	-2.433288	1.628170
H	-6.779937	-4.072129	1.181215
H	-5.686713	-5.602793	3.609107
H	-5.432062	-6.139906	1.940449
H	-4.044910	-5.935704	3.037059
H	-5.000491	-3.412102	4.414044
H	-3.340246	-3.525417	3.809936
H	-4.376821	-2.151622	3.330539
H	-5.567987	-3.640410	-2.321590
H	-6.082483	-3.080543	-0.719337
H	-4.655677	-2.353290	-1.523444
H	-3.366544	-4.722451	-2.651828
H	-2.472436	-3.525134	-1.693604
H	-2.347514	-5.256308	-1.302887
H	-5.201246	-6.044660	-1.689593
H	-4.358660	-6.475805	-0.191887
H	-5.938641	-5.649707	-0.128963
H	-1.851209	1.234817	4.360899
H	-2.822934	1.855838	3.013854
H	-2.816771	0.118228	3.372927

H	0.278968	2.294045	3.410064
H	0.746016	2.013829	1.724252
H	-0.704314	2.979741	2.107118
H	0.173665	-0.071496	4.033672
H	-0.685167	-1.272318	3.048235
H	0.797368	-0.508400	2.431647
H	-1.572517	3.363371	-1.509394
H	-0.613110	3.157513	-0.034667
H	-0.351237	2.084518	-1.430529
H	-3.794289	3.145581	-0.258461
H	-4.044729	1.794847	0.864607
H	-2.848830	3.073402	1.237186
H	-3.234872	1.732132	-2.241735
H	-2.193214	0.310485	-2.074465
H	-3.756968	0.389779	-1.207128
O	-4.552540	-0.593712	1.343756

[(PCP)Au-H]+ - O2 TSII open shell singlet H-abstraction

73

C	1.192342	2.457064	0.130680
C	0.004786	1.752745	-0.143012
C	-1.178752	2.435412	-0.479799
C	-1.149976	3.832004	-0.567765
C	0.028414	4.535652	-0.331638
C	1.190347	3.852009	0.019200
AU	-0.008221	-0.274398	0.020033
P	-2.354486	-0.015915	-0.086235
C	-3.048089	-0.009114	1.654080
C	-2.566285	-1.269235	2.391737
C	-2.458141	1.692905	-0.752189
C	2.436717	1.739335	0.576354
P	2.348300	-0.034239	0.103494
C	3.119585	-0.185048	-1.599277
C	2.912522	-1.602305	-2.156304
C	3.165292	-0.997803	1.481068
C	3.362601	-2.459627	1.054992
C	-3.223653	-1.102437	-1.337259
C	-3.300322	-2.547385	-0.825736
C	-4.625489	-0.558443	-1.656073
C	-2.347936	-1.063369	-2.605281
C	-2.460713	1.227261	2.358294
C	-4.580192	0.066446	1.655882
C	4.506996	-0.361721	1.879545
C	2.191893	-0.933855	2.673726
C	2.369439	0.818211	-2.496115
C	4.613181	0.166765	-1.556643
H	-0.904667	-2.751101	0.725603
H	-2.065123	4.364625	-0.824913
H	0.039242	5.621392	-0.412688
H	2.111190	4.399753	0.217681
H	3.346948	2.208584	0.183710
H	2.512504	1.773195	1.672670
H	-2.621091	1.601711	-1.834968
H	-3.332352	2.216942	-0.345969
H	-3.800069	-3.158066	-1.590323
H	-2.292089	-2.963514	-0.692996
H	-3.880951	-2.639403	0.098413

H	-5.035095	-1.155791	-2.482532
H	-5.312726	-0.642982	-0.809882
H	-4.603398	0.486761	-1.987492
H	-2.829212	-1.691803	-3.367349
H	-2.256679	-0.053490	-3.023577
H	-1.349369	-1.467525	-2.395504
H	-2.978024	-1.258156	3.409676
H	-2.901802	-2.196200	1.913280
H	-1.472355	-1.283943	2.480824
H	-2.784012	1.204117	3.407652
H	-1.363879	1.225723	2.342003
H	-2.813673	2.166700	1.918597
H	-4.917216	0.205416	2.692325
H	-4.955212	0.913400	1.068344
H	-5.035960	-0.855509	1.279505
H	3.138153	-1.583184	-3.231387
H	3.585783	-2.326062	-1.686573
H	1.879877	-1.946631	-2.006829
H	4.988694	0.184355	-2.589432
H	4.806655	1.153933	-1.118939
H	5.192176	-0.583756	-1.008207
H	2.796449	0.749999	-3.505577
H	1.300470	0.581307	-2.563210
H	2.474391	1.854544	-2.155396
H	4.917451	-0.936614	2.721397
H	5.239952	-0.389020	1.067688
H	4.393706	0.676168	2.214306
H	3.657010	-3.040575	1.939754
H	2.442162	-2.894937	0.649115
H	4.161013	-2.559077	0.311896
H	2.666584	-1.427405	3.532776
H	1.957504	0.095607	2.972625
H	1.255988	-1.460467	2.455290
O	-0.002342	-2.434161	0.508811
O	0.054913	-2.601528	-0.993909

[(PCP)Au-H]+ - O2 PC open shell singlet H-abstraction

73

C	1.400229	-3.853481	0.111263
C	1.345711	-2.465747	-0.059559
C	0.127846	-1.794676	0.160050
C	-1.018304	-2.506003	0.562760
C	-0.942296	-3.895142	0.712205
C	0.260535	-4.563354	0.487201
C	2.574736	-1.704234	-0.487281
P	2.382230	0.085214	-0.097598
C	3.191012	1.025424	-1.493100
C	2.247273	0.852262	-2.699296
AU	0.034343	0.246448	-0.050601
O	-0.239369	2.840098	-1.460093
O	-0.013799	2.347311	-0.092212
C	-2.302395	-1.777880	0.873198
P	-2.294567	-0.111942	0.089380
C	-3.148720	1.045437	1.284333
C	-2.163634	1.220099	2.457220
C	-3.078445	-0.302119	-1.601657
C	-4.595472	-0.504644	-1.491778
C	-2.756038	0.928051	-2.462748

C	-2.429301	-1.536589	-2.254154
C	3.069289	0.385226	1.615458
C	4.601584	0.309310	1.623965
C	2.586416	1.755511	2.119370
C	2.485958	-0.711404	2.524907
C	3.293540	2.514397	-1.134212
C	4.575212	0.447945	-1.827929
C	-3.383916	2.410198	0.623168
C	-4.468895	0.451517	1.798271
H	-1.212845	2.941223	-1.476529
H	-1.828770	-4.452261	1.014839
H	0.311703	-5.644160	0.611903
H	2.341482	-4.377924	-0.052836
H	3.490319	-2.107275	-0.036139
H	2.704078	-1.775909	-1.576711
H	-2.386363	-1.615815	1.957431
H	-3.189850	-2.348511	0.571960
H	-3.788883	3.097523	1.378499
H	-2.439741	2.836818	0.261816
H	-4.106741	2.357883	-0.197917
H	-4.883211	1.139831	2.548065
H	-5.211999	0.335619	1.003925
H	-4.324801	-0.519320	2.286920
H	-2.641316	1.849885	3.220033
H	-1.900187	0.267671	2.934045
H	-1.244509	1.721827	2.130627
H	-3.191989	0.779706	-3.459817
H	-3.181214	1.850824	-2.053176
H	-1.673510	1.055756	-2.586220
H	-2.823485	-1.624088	-3.275579
H	-1.338544	-1.440482	-2.319191
H	-2.663596	-2.465437	-1.722598
H	-4.985526	-0.739160	-2.491946
H	-4.858437	-1.338792	-0.829609
H	-5.104774	0.399548	-1.141046
H	2.920162	1.878350	3.158675
H	2.990746	2.585170	1.532709
H	1.491732	1.827560	2.102388
H	4.946854	0.381794	2.664638
H	4.973003	-0.639527	1.216999
H	5.055589	1.135226	1.065699
H	2.838709	-0.525493	3.548203
H	1.389121	-0.693437	2.535775
H	2.811227	-1.716689	2.233774
H	4.978386	1.008895	-2.682724
H	5.282914	0.547983	-0.999330
H	4.526699	-0.607501	-2.120997
H	3.607738	3.065369	-2.031347
H	2.327317	2.912828	-0.803083
H	4.043889	2.694544	-0.356052
H	2.710959	1.334078	-3.571373
H	2.083060	-0.202188	-2.956030
H	1.278270	1.333502	-2.519860

[(PCP)Pd-H]

71

PD	-0.001946	0.000261	0.197703
H	-0.029501	0.000569	1.841728

C	0.034109	0.000008	-1.876546
C	-1.166503	0.000250	-2.631437
C	-1.135604	-0.000036	-4.033603
C	0.084074	-0.000543	-4.713911
C	1.279129	-0.000705	-3.991292
C	1.260478	-0.000429	-2.589040
C	-2.504795	0.000924	-1.925187
H	-2.069442	0.000150	-4.599717
H	0.103487	-0.000798	-5.804259
H	2.232344	-0.001052	-4.524153
C	2.573314	-0.000608	-1.836888
P	2.263734	-0.000042	-0.001303
H	3.180439	-0.875883	-2.106618
H	3.180988	0.874126	-2.107130
P	-2.260128	0.000352	-0.079452
H	-3.103021	-0.873595	-2.216400
H	-3.101812	0.876435	-2.215890
C	-3.102642	-1.584156	0.497099
C	-3.103072	1.584092	0.498546
C	-2.272954	2.707433	-0.153939
C	-4.571777	1.715627	0.077699
C	-2.974448	1.710880	2.023798
C	-4.571409	-1.715405	0.076447
C	-2.272570	-2.706534	-0.157104
C	-2.973531	-1.712750	2.022183
H	-3.247008	-2.735451	2.320812
H	-1.942677	-1.514791	2.343393
H	-3.641680	-1.022218	2.548531
H	-4.921685	-2.731446	0.311859
H	-5.214621	-1.011412	0.614988
H	-4.714804	-1.559156	-1.000376
H	-2.656390	-3.679509	0.182352
H	-2.329396	-2.683517	-1.252584
H	-1.215094	-2.622891	0.128665
H	-3.248362	2.733128	2.323579
H	-3.642542	1.019491	2.549099
H	-1.943624	1.512874	2.345091
H	-2.656919	3.679906	0.186793
H	-1.215516	2.623516	0.131900
H	-2.329608	2.685952	-1.249454
H	-4.922280	2.731311	0.314313
H	-4.714927	1.560703	-0.999348
H	-5.214973	1.010853	0.615229
C	3.084368	1.584877	0.604342
C	3.084026	-1.584594	0.605811
C	2.275313	-2.706309	-0.075900
C	4.566214	-1.717520	0.236265
C	2.902143	-1.711318	2.125663
C	4.566418	1.717270	0.234127
C	2.275572	2.706032	-0.078194
C	2.902988	1.712809	2.124152
H	3.163550	-2.734025	2.434693
H	3.552992	-1.021249	2.673857
H	1.861167	-1.511233	2.410859
H	4.907762	-2.733221	0.485599
H	4.746864	-1.563456	-0.835201
H	5.190801	-1.012715	0.795373
H	2.645280	-3.679598	0.277825
H	1.208675	-2.620347	0.172647
H	2.370376	-2.684655	-1.168707

H	4.908269	2.733086	0.482573
H	5.191047	1.012734	0.793503
H	4.746607	1.562359	-0.837294
H	3.164358	2.735797	2.432283
H	1.862144	1.512791	2.409875
H	3.554150	1.023261	2.672614
H	2.645936	3.679621	0.274286
H	2.370091	2.683131	-1.171034
H	1.209039	2.620560	0.170971

[(PCP)Pd-H] - O2 RC triplet

73

C	1.197969	-2.590205	0.283208
C	-0.013963	-1.891953	0.064048
C	-1.232990	-2.575116	0.290943
C	-1.233750	-3.881163	0.799157
C	-0.027081	-4.534661	1.062476
C	1.185900	-3.896084	0.791555
PD	-0.002897	0.125845	-0.437572
P	-2.254724	-0.061223	-0.206554
C	-3.324119	0.476813	-1.657476
C	-3.399717	2.009106	-1.691983
C	-2.534667	-1.893043	-0.067724
C	2.505531	-1.923750	-0.083239
P	2.247565	-0.088063	-0.212835
C	2.842326	0.594607	1.442535
C	2.360873	2.047533	1.584993
C	3.320983	0.445195	-1.662598
C	3.419261	1.976475	-1.685436
C	-2.838000	0.636032	1.446644
C	-2.342055	2.084886	1.580846
C	-4.356166	0.568120	1.644586
C	-2.139940	-0.211734	2.525967
C	-2.550463	-0.006319	-2.899960
C	-4.732454	-0.133642	-1.663590
C	4.719767	-0.186485	-1.676803
C	2.537871	-0.016777	-2.907120
C	2.139487	-0.251531	2.519964
C	4.360343	0.510570	1.635733
H	0.005693	1.725120	-0.805196
H	-2.179399	-4.392038	0.991048
H	-0.032114	-5.546679	1.468505
H	2.126438	-4.418430	0.977673
H	3.323889	-2.180014	0.603546
H	2.821361	-2.268139	-1.079679
H	-3.350518	-2.135714	0.626922
H	-2.863418	-2.237782	-1.059830
H	-2.563533	2.447134	2.595511
H	-1.259437	2.139341	1.410479
H	-2.826804	2.758711	0.866650
H	-4.596747	0.865898	2.676096
H	-4.886810	1.251859	0.972004
H	-4.748774	-0.445643	1.492403
H	-2.349126	0.229226	3.511057
H	-2.493405	-1.249613	2.535719
H	-1.053664	-0.224855	2.372511
H	-3.838094	2.327559	-2.649050
H	-4.032117	2.405176	-0.888589

H	-2.399416	2.453206	-1.603381
H	-3.093376	0.302610	-3.805295
H	-1.542625	0.429546	-2.922387
H	-2.449217	-1.099395	-2.924446
H	-5.245390	0.165163	-2.589901
H	-4.705964	-1.230136	-1.641893
H	-5.339318	0.211626	-0.820900
H	2.588496	2.403091	2.600655
H	2.849815	2.720023	0.872440
H	1.278249	2.112815	1.418123
H	4.606805	0.802089	2.667631
H	4.741983	-0.506653	1.478859
H	4.896136	1.191215	0.964105
H	2.355840	0.182858	3.506438
H	1.052678	-0.253899	2.369979
H	2.483201	-1.292708	2.523800
H	5.236607	0.114887	-2.600101
H	5.332534	0.139450	-0.830671
H	4.675983	-1.282579	-1.667825
H	3.859933	2.295727	-2.641171
H	2.425843	2.434626	-1.591019
H	4.059401	2.357033	-0.880712
H	3.083880	0.290355	-3.811197
H	2.420113	-1.108032	-2.939258
H	1.536814	0.434535	-2.924527
O	0.035025	4.018122	-0.746253
O	0.033498	4.528285	0.378800

[(PCP)Pd-H] - O2 RC open shell singlet

73

C	1.213930	2.603878	0.105016
C	-0.002552	1.887306	0.179516
C	-1.220885	2.600247	0.101489
C	-1.215122	3.983436	-0.126283
C	-0.006013	4.671588	-0.252511
C	1.204788	3.987010	-0.122686
PD	0.000563	-0.191672	0.257882
P	-2.258226	0.040184	0.106244
C	-3.299555	-0.754227	1.456817
C	-3.387883	-2.266866	1.211929
C	-2.525258	1.866778	0.319066
C	2.519607	1.873810	0.325988
P	2.258756	0.047153	0.106698
C	2.876296	-0.322545	-1.636198
C	2.416440	-1.732519	-2.041090
C	3.304505	-0.748900	1.453046
C	3.400550	-2.259754	1.200335
C	-2.875488	-0.337870	-1.634782
C	-2.415070	-1.749472	-2.033024
C	-4.394145	-0.203622	-1.793199
C	-2.166866	0.674875	-2.553559
C	-2.498981	-0.508075	2.750680
C	-4.703268	-0.150314	1.603150
C	4.705186	-0.138503	1.601911
C	2.503483	-0.513496	2.748604
C	2.167899	0.693929	-2.550943
C	4.394965	-0.186990	-1.793554
H	0.002586	-1.840587	0.271794

H	-2.158945	4.526319	-0.204940
H	-0.007352	5.745660	-0.440479
H	2.147234	4.532670	-0.198580
H	3.331747	2.252468	-0.309360
H	2.847390	2.024434	1.365292
H	-3.335600	2.240851	-0.321272
H	-2.858444	2.020078	1.356242
H	-2.646295	-1.914213	-3.095601
H	-1.333729	-1.862649	-1.883185
H	-2.916967	-2.530368	-1.452576
H	-4.654654	-0.307807	-2.856938
H	-4.931772	-0.985337	-1.244473
H	-4.761374	0.774578	-1.456605
H	-2.403506	0.428483	-3.598466
H	-2.487560	1.706397	-2.366422
H	-1.078325	0.628210	-2.421775
H	-3.805585	-2.751963	2.106076
H	-4.044716	-2.505723	0.367170
H	-2.395813	-2.695317	1.017993
H	-3.030183	-0.969785	3.595785
H	-1.495776	-0.948807	2.677452
H	-2.384830	0.561916	2.968914
H	-5.199811	-0.613431	2.468698
H	-4.670316	0.931160	1.783835
H	-5.328718	-0.332286	0.723760
H	2.652371	-1.893903	-3.103147
H	2.914753	-2.515856	-1.460817
H	1.334312	-1.844506	-1.896590
H	4.655759	-0.286368	-2.857683
H	4.761557	0.789930	-1.452608
H	4.932908	-0.970785	-1.248120
H	2.405270	0.452128	-3.596767
H	1.079312	0.646554	-2.420036
H	2.488283	1.724680	-2.359158
H	5.204575	-0.603751	2.464679
H	5.331155	-0.312363	0.721248
H	4.666615	0.941744	1.788624
H	3.820975	-2.747344	2.091846
H	2.410747	-2.692516	1.004367
H	4.058346	-2.490831	0.354156
H	3.036841	-0.978103	3.590754
H	2.385443	0.554766	2.973135
H	1.501956	-0.957675	2.673258
O	0.018101	-3.808435	0.185180
O	-0.041813	-4.296812	-0.969662

[(PCP)Pd-H] - O2 INT open shell singlet H-abstraction

73

C	1.210988	-2.555513	0.208826
C	-0.001803	-1.835459	0.056237
C	-1.220245	-2.516042	0.305091
C	-1.210901	-3.834512	0.777846
C	-0.007341	-4.508355	0.981707
C	1.197569	-3.873702	0.679997
PD	0.014851	0.142345	-0.381206
P	-2.271862	-0.024464	-0.147805
C	-3.321793	0.501526	-1.614550

C	-3.369063	2.033210	-1.705721
C	-2.536286	-1.844298	0.002261
C	2.518935	-1.911799	-0.178955
P	2.300154	-0.080354	-0.177600
C	2.892599	0.497646	1.514140
C	2.387362	1.930902	1.748408
C	3.343309	0.553539	-1.604725
C	3.473619	2.080841	-1.516505
C	-2.830052	0.706324	1.496404
C	-2.272987	2.134875	1.615576
C	-4.351129	0.706931	1.681381
C	-2.171081	-0.153399	2.590164
C	-2.546630	-0.027238	-2.838860
C	-4.739646	-0.085564	-1.592894
C	4.726306	-0.106337	-1.685410
C	2.518705	0.206147	-2.860577
C	2.219259	-0.425508	2.545672
C	4.414824	0.427480	1.679643
H	-0.750630	2.684463	-0.489425
H	-2.157715	-4.335693	0.984580
H	-0.008754	-5.529878	1.361810
H	2.141276	-4.404832	0.812504
H	3.356259	-2.235049	0.453112
H	2.775201	-2.197165	-1.209987
H	-3.317023	-2.087442	0.734619
H	-2.906635	-2.195111	-0.972012
H	-2.566663	2.558998	2.586287
H	-1.175012	2.125543	1.576756
H	-2.653641	2.804476	0.835548
H	-4.586018	1.025811	2.707472
H	-4.845956	1.404936	0.996565
H	-4.783633	-0.291088	1.536186
H	-2.350052	0.320141	3.565597
H	-2.581960	-1.168875	2.626711
H	-1.086702	-0.227157	2.434750
H	-3.927349	2.312130	-2.611910
H	-3.883087	2.489906	-0.852016
H	-2.352401	2.443707	-1.814795
H	-3.107299	0.230450	-3.749712
H	-1.554551	0.443652	-2.887756
H	-2.425984	-1.118437	-2.818473
H	-5.239632	0.176257	-2.537080
H	-4.736501	-1.179881	-1.515790
H	-5.344256	0.315387	-0.773709
H	2.652652	2.241016	2.769392
H	2.824499	2.650230	1.048789
H	1.295708	1.978514	1.645213
H	4.671528	0.668953	2.721584
H	4.806516	-0.575323	1.465847
H	4.931145	1.148682	1.036188
H	2.440960	-0.045452	3.552902
H	1.130088	-0.441907	2.413032
H	2.585296	-1.456952	2.484480
H	5.237895	0.261311	-2.587278
H	5.356086	0.134711	-0.823066
H	4.658762	-1.198170	-1.769027
H	3.949575	2.445986	-2.438424
H	2.487354	2.553671	-1.432512
H	4.102209	2.393882	-0.674524
H	3.085495	0.516672	-3.751143

H	2.327874	-0.872288	-2.946451
H	1.557170	0.741316	-2.846653
O	0.089196	2.326083	-0.836619
O	-0.102206	2.475134	-2.309282

[(PCP)Pd-H] - O2 PC triplet H-abstraction

73

C	1.268188	2.248933	0.704544
C	0.038276	1.558835	0.699862
C	-1.170225	2.278866	0.615270
C	-1.139679	3.677023	0.521476
C	0.080456	4.357915	0.525762
C	1.280084	3.647022	0.616827
PD	0.022692	-0.502563	0.254208
P	-2.304950	-0.179167	-0.070168
C	-3.644518	-1.132759	0.871677
C	-3.781980	-2.534412	0.260488
C	-2.489935	1.541070	0.613463
C	2.562153	1.469093	0.765136
P	2.353085	-0.195775	-0.036846
C	2.790977	0.094931	-1.864307
C	2.181328	-1.062282	-2.672576
C	3.652928	-1.258053	0.833980
C	3.707165	-2.622801	0.133079
C	-2.729239	-0.011965	-1.911098
C	-2.278222	-1.299389	-2.619700
C	-4.203880	0.270189	-2.213618
C	-1.873428	1.151917	-2.436006
C	-3.125405	-1.247322	2.319278
C	-5.018903	-0.446818	0.913123
C	5.051964	-0.628641	0.912969
C	3.129382	-1.459107	2.268493
C	2.108198	1.405751	-2.287279
C	4.289447	0.211520	-2.161324
H	-1.294517	-3.187728	1.413185
H	-2.073613	4.236059	0.434848
H	0.097068	5.445037	0.445654
H	2.231504	4.182486	0.601991
H	3.401303	2.031217	0.334122
H	2.824117	1.264012	1.813810
H	-3.272400	2.103746	0.086830
H	-2.839280	1.416311	1.649224
H	-2.393205	-1.175221	-3.706555
H	-1.222194	-1.510965	-2.402772
H	-2.867184	-2.171497	-2.315065
H	-4.311855	0.489271	-3.286602
H	-4.840744	-0.592972	-1.990599
H	-4.582469	1.138082	-1.658881
H	-1.980657	1.209467	-3.528679
H	-2.172624	2.116849	-2.010641
H	-0.813165	0.994188	-2.204093
H	-4.379873	-3.170891	0.928261
H	-4.286339	-2.505524	-0.712627
H	-2.806422	-3.018734	0.108598
H	-3.763530	-1.951825	2.871997
H	-2.079943	-1.581970	2.370242
H	-3.175616	-0.282012	2.840073
H	-5.670015	-1.011480	1.597807

H	-4.952490	0.578546	1.297057
H	-5.508709	-0.419584	-0.063530
H	2.349263	-0.887196	-3.745502
H	2.626238	-2.029563	-2.411172
H	1.099651	-1.130293	-2.493409
H	4.418751	0.483350	-3.220015
H	4.768716	0.992981	-1.558553
H	4.819352	-0.733481	-1.998591
H	2.179680	1.504632	-3.380144
H	1.048609	1.422568	-2.009487
H	2.586333	2.282255	-1.834914
H	5.679475	-1.256985	1.563769
H	5.546397	-0.561451	-0.059148
H	5.023014	0.374314	1.357264
H	4.339624	-3.307136	0.717527
H	2.705158	-3.065914	0.055109
H	4.135819	-2.552905	-0.873930
H	3.794437	-2.166385	2.786459
H	3.139499	-0.522031	2.841093
H	2.105530	-1.853732	2.270971
O	-0.311325	-3.180111	1.401416
O	-0.006148	-1.893256	1.965447

[(PCP)Pd-H] - O2 TSII open shell singlet H-abstraction

73

C	1.240905	2.316442	0.801631
C	0.016948	1.617925	0.616295
C	-1.189292	2.360248	0.723539
C	-1.157223	3.747878	0.908811
C	0.055398	4.426594	1.023847
C	1.247737	3.703650	0.987548
PD	-0.001871	-0.342990	0.162819
P	-2.281020	-0.038113	0.002528
C	-3.370955	-1.149054	1.059447
C	-3.397641	-2.561004	0.457208
C	-2.518423	1.651468	0.696577
C	2.540513	1.555860	0.843325
P	2.284682	-0.083006	0.044010
C	2.856507	0.140627	-1.740164
C	2.331928	-1.031170	-2.584710
C	3.344376	-1.280032	1.030821
C	3.394791	-2.630590	0.302743
C	-2.836388	0.090705	-1.797436
C	-2.341915	-1.137889	-2.577117
C	-4.353156	0.243346	-1.956974
C	-2.123932	1.330535	-2.368368
C	-2.646687	-1.217615	2.418957
C	-4.794946	-0.614135	1.260233
C	4.758276	-0.757110	1.318386
C	2.581312	-1.467098	2.357256
C	2.191009	1.433590	-2.244485
C	4.378468	0.261715	-1.879340
H	-0.891895	-2.720410	-0.991276
H	-2.097668	4.298264	0.965628
H	0.070856	5.508288	1.155413
H	2.201889	4.219739	1.104994
H	3.375292	2.115015	0.400853

H	2.815794	1.356723	1.889565
H	-3.289787	2.215894	0.156883
H	-2.888126	1.526623	1.724752
H	-2.628780	-1.031895	-3.633053
H	-1.247634	-1.210912	-2.532429
H	-2.777753	-2.074744	-2.210980
H	-4.579508	0.466803	-3.009822
H	-4.884372	-0.678210	-1.693366
H	-4.753819	1.064401	-1.349124
H	-2.308377	1.374713	-3.450927
H	-2.488186	2.264047	-1.924543
H	-1.040732	1.271627	-2.201882
H	-3.960764	-3.224200	1.130597
H	-3.890893	-2.590601	-0.521118
H	-2.372809	-2.956682	0.387053
H	-3.209567	-1.889424	3.083672
H	-1.630650	-1.612082	2.284084
H	-2.587576	-0.237072	2.909194
H	-5.305086	-1.251325	1.997816
H	-4.802057	0.410999	1.651924
H	-5.384082	-0.635017	0.338733
H	2.584281	-0.850753	-3.639598
H	2.770075	-1.989671	-2.288136
H	1.242189	-1.116928	-2.494635
H	4.619986	0.519897	-2.920996
H	4.791643	1.050757	-1.237966
H	4.886675	-0.680767	-1.646567
H	2.394390	1.535672	-3.319860
H	1.104434	1.402284	-2.096623
H	2.577888	2.326610	-1.740092
H	5.266336	-1.471995	1.982682
H	5.362562	-0.658058	0.411545
H	4.743394	0.212905	1.831214
H	3.873019	-3.370880	0.961103
H	2.381012	-2.980818	0.070695
H	3.984778	-2.578973	-0.620190
H	3.142131	-2.171747	2.989302
H	2.482587	-0.526048	2.914945
H	1.580197	-1.873790	2.157121
O	0.022326	-2.535495	-0.697664
O	-0.088282	-2.768630	0.792582

[(PCP)Pd-H] - O2 PC open shell singlet H-abstraction

73

PD	0.006622	0.333191	-0.041832
O	0.076811	2.424249	-0.330046
C	0.015428	-1.697206	0.059874
C	-1.145260	-2.445462	-0.254773
C	-1.137138	-3.843749	-0.176703
C	0.022770	-4.525550	0.195289
C	1.182430	-3.806172	0.487735
C	1.185726	-2.406965	0.425709
C	-2.394016	-1.721264	-0.695337
H	-2.044567	-4.402661	-0.412292
H	0.024303	-5.614213	0.253037
H	2.094409	-4.335852	0.768841
C	2.444736	-1.642242	0.758815
P	2.279692	0.071113	0.085718

H	2.551399	-1.542808	1.849227
H	3.351082	-2.149385	0.401129
P	-2.276565	0.041988	-0.148235
H	-3.311472	-2.211816	-0.342925
H	-2.449548	-1.703987	-1.794057
C	-3.136366	0.090597	1.528528
C	-3.169796	1.019988	-1.481145
C	-2.187688	1.038190	-2.669575
C	-4.499436	0.394926	-1.924182
C	-3.376493	2.463007	-0.999800
C	-4.643825	-0.173740	1.453425
C	-2.462737	-1.004257	2.377788
C	-2.860557	1.449002	2.192836
H	-3.179859	1.401166	3.244444
H	-1.793758	1.701690	2.153707
H	-3.411796	2.262977	1.710869
H	-5.042856	-0.260536	2.475206
H	-5.176840	0.647177	0.960142
H	-4.877470	-1.108434	0.927107
H	-2.870966	-0.952262	3.397448
H	-2.650208	-2.011158	1.986504
H	-1.376261	-0.856757	2.425643
H	-3.696174	3.081254	-1.851763
H	-4.156045	2.528980	-0.231753
H	-2.440675	2.875653	-0.601489
H	-2.645002	1.595514	-3.500566
H	-1.249425	1.530587	-2.384785
H	-1.953777	0.028481	-3.031986
H	-4.911058	0.989531	-2.753532
H	-4.370335	-0.632083	-2.288096
H	-5.242997	0.383662	-1.120993
C	3.103182	0.049449	-1.605868
C	3.155034	1.169663	1.333869
C	2.219058	1.191557	2.559235
C	4.532966	0.652417	1.768583
C	3.263275	2.588811	0.754318
C	4.627476	-0.095366	-1.552683
C	2.494294	-1.150983	-2.354307
C	2.706145	1.331525	-2.356812
H	3.508167	3.295325	1.560461
H	4.058109	2.656920	0.001839
H	2.319090	2.892156	0.278471
H	4.922868	1.306976	2.562464
H	4.478994	-0.364434	2.177448
H	5.256987	0.655948	0.948245
H	2.626895	1.886089	3.307910
H	1.204923	1.514878	2.289227
H	2.136263	0.203802	3.030575
H	5.010030	-0.234735	-2.574741
H	5.108048	0.799191	-1.140311
H	4.940278	-0.964781	-0.959955
H	3.049354	1.255332	-3.398808
H	1.616155	1.461590	-2.354895
H	3.154835	2.227944	-1.917160
H	2.869714	-1.143575	-3.387751
H	2.770511	-2.109429	-1.898789
H	1.398872	-1.088060	-2.377868
O	-0.290715	3.160835	0.918241
H	0.594015	3.301947	1.308764

[(CNC)Au-H]

55

C	0.028854	-0.170008	0.245273
N	-0.028573	-0.053104	1.590509
C	-0.312095	1.082677	2.266931
C	-0.572781	2.238841	1.524793
C	-0.525970	2.165838	0.131575
C	-0.226096	0.970406	-0.523050
AU	0.343872	-1.740359	2.700324
C	-0.297462	0.913081	3.729011
C	0.362896	-1.528944	-0.211682
C	0.572868	-2.531620	0.787552
H	0.627794	-3.035798	3.553178
H	-0.807051	3.176242	2.022438
H	-0.727013	3.060190	-0.456268
H	-0.191834	0.926199	-1.608472
C	-0.555688	1.969436	4.612963
C	-0.523610	1.759517	5.987088
C	-0.234842	0.494475	6.525975
C	0.019843	-0.557652	5.629229
C	-0.002078	-0.384795	4.243528
H	-0.782556	2.965317	4.231555
H	-0.726360	2.600343	6.649233
C	-0.204331	0.308481	8.045909
H	0.245008	-1.546016	6.023700
C	0.480489	-1.861235	-1.564343
C	0.799719	-3.164109	-1.949026
C	1.008952	-4.168089	-0.995867
C	0.888099	-3.819261	0.365825
H	1.049374	-4.589221	1.120198
C	1.359055	-5.609146	-1.375928
H	0.883605	-3.387921	-3.009861
H	0.324070	-1.105102	-2.334100
C	0.121861	-1.136157	8.452657
C	0.871984	1.239525	8.645168
C	-1.585185	0.680289	8.628250
C	1.453555	-5.807404	-2.895779
C	2.720934	-5.978812	-0.749135
C	0.267881	-6.553289	-0.826369
H	0.129784	-1.212518	9.547849
H	-0.626538	-1.844010	8.072290
H	1.109616	-1.447677	8.087628
H	0.904908	1.121764	9.737163
H	1.864021	0.996091	8.241482
H	0.662554	2.293388	8.423313
H	-1.577095	0.559089	9.720265
H	-1.851017	1.720784	8.404094
H	-2.368348	0.030800	8.214657
H	0.509284	-7.594541	-1.080765
H	0.183877	-6.480840	0.265166
H	-0.710746	-6.309499	-1.261362
H	2.982225	-7.015327	-1.003379
H	3.513862	-5.319966	-1.127874
H	2.698894	-5.892332	0.344232
H	1.706757	-6.853768	-3.111124
H	0.501167	-5.583428	-3.394436
H	2.234210	-5.175291	-3.339551

[(CNC)Au-H] - O2 RC triplet

57

C	0.980294	-3.787162	0.404637
C	0.643867	-2.503539	0.819913
C	0.411858	-1.510950	-0.184595
C	0.530754	-1.849634	-1.535490
C	0.871752	-3.149005	-1.913511
C	1.102057	-4.143061	-0.954990
C	0.052850	-0.155929	0.265323
N	-0.004381	-0.032637	1.609812
C	-0.309345	1.100709	2.280794
C	-0.593544	2.247644	1.532875
C	-0.548081	2.167754	0.139960
C	-0.225933	0.974920	-0.508877
AU	0.403674	-1.706317	2.729216
C	0.031552	-0.350156	4.264977
C	-0.290722	0.938598	3.743865
C	-0.572068	1.993222	4.622868
C	-0.537863	1.790105	5.998057
C	-0.222850	0.534004	6.543103
C	0.055807	-0.516259	5.651372
C	-0.192357	0.354666	8.063774
C	-1.584450	0.692251	8.639803
C	1.475770	-5.580264	-1.326162
C	0.409028	-6.540577	-0.756974
C	0.170124	-1.079431	8.477110
C	0.857176	1.315428	8.663614
C	1.557848	-5.790344	-2.845037
C	2.851382	-5.917204	-0.711061
H	0.713922	-2.987952	3.590668
H	-0.844990	3.183111	2.025717
H	-0.767988	3.054698	-0.452330
H	-0.192904	0.925489	-1.594116
H	-0.819434	2.982386	4.236681
H	-0.759976	2.629221	6.656137
H	0.301726	-1.497890	6.050342
H	1.157068	-4.550167	1.162580
H	0.955296	-3.377985	-2.973265
H	0.357961	-1.101222	-2.309259
H	0.177048	-1.151209	9.572599
H	-0.558291	-1.808378	8.097748
H	1.166783	-1.366292	8.116020
H	0.888744	1.202335	9.756141
H	1.856806	1.096197	8.264829
H	0.621726	2.362766	8.437351
H	-1.577420	0.574266	9.732180
H	-1.876179	1.724953	8.411614
H	-2.348922	0.021560	8.225039
H	0.668784	-7.579230	-1.003410
H	0.332995	-6.459667	0.334339
H	-0.578412	-6.320500	-1.184469
H	3.127944	-6.952567	-0.953368
H	3.627721	-5.249982	-1.108921
H	2.841991	-5.814469	0.380821
H	1.827956	-6.833764	-3.053958
H	0.596267	-5.588144	-3.335290
H	2.322020	-5.148153	-3.302790
O	0.831174	-5.641237	4.075586

O	1.383229	-6.179338	3.126015

[(CNC)Au-H] - O2 RC open shell singlet

57

C	0.940930	-3.798354	0.404487
C	0.615892	-2.512024	0.819431
C	0.405498	-1.514093	-0.184538
C	0.533241	-1.851173	-1.535004
C	0.862993	-3.153472	-1.912889
C	1.072539	-4.152513	-0.954739
C	0.060526	-0.155612	0.265669
N	-0.006830	-0.034906	1.609942
C	-0.301160	1.101003	2.281258
C	-0.562998	2.253603	1.533914
C	-0.506389	2.176647	0.141259
C	-0.195061	0.981113	-0.507890
AU	0.369765	-1.716462	2.728805
C	0.005062	-0.358547	4.264808
C	-0.295043	0.935698	3.744098
C	-0.565250	1.992988	4.623397
C	-0.540694	1.787297	5.998382
C	-0.247556	0.525711	6.543014
C	0.019435	-0.527333	5.650997
C	-0.225268	0.344284	8.063591
C	-1.611083	0.711392	8.636865
C	1.433999	-5.592856	-1.325725
C	0.345280	-6.541218	-0.778675
C	0.104958	-1.097692	8.476592
C	0.843363	1.281884	8.666359
C	1.539030	-5.798103	-2.843910
C	2.794566	-5.950475	-0.689200
H	0.657711	-3.003287	3.590577
H	-0.805465	3.191291	2.027050
H	-0.708619	3.068078	-0.450615
H	-0.152404	0.934159	-1.592907
H	-0.795607	2.986395	4.237657
H	-0.752874	2.628809	6.656679
H	0.248156	-1.513282	6.049504
H	1.102775	-4.565157	1.162298
H	0.954982	-3.380736	-2.972340
H	0.376629	-1.099012	-2.308588
H	0.105836	-1.170833	9.571998
H	-0.637452	-1.810249	8.093266
H	1.096735	-1.405550	8.119334
H	0.870288	1.167659	9.758885
H	1.838860	1.041532	8.269399
H	0.630725	2.334117	8.439941
H	-1.608660	0.593251	9.729227
H	-1.880491	1.750013	8.408413
H	-2.388841	0.057120	8.220641
H	0.597014	-7.582540	-1.022216
H	0.249287	-6.460855	0.311083
H	-0.631063	-6.308415	-1.224520
H	3.062916	-6.987310	-0.934332
H	3.585718	-5.289824	-1.068313
H	2.767221	-5.856086	0.403193
H	1.799782	-6.843983	-3.052395
H	0.588335	-5.582402	-3.349399

```
H         2.318555   -5.163527   -3.286123
O         1.798949   -5.338593    4.079594
O         1.635155   -6.066336    3.108795
```

[(CNC)Au-H] - O2 TSI open shell singlet H-abstraction

57

```
C        -3.088161   -0.666944    0.062879
C        -2.023957    0.216610    0.013925
C        -2.291270    1.618721   -0.036562
C        -3.616201    2.063080   -0.036804
C        -4.669032    1.146640    0.013694
C        -4.431782   -0.233353    0.063623
C        -1.134468    2.524773   -0.089197
N         0.060437    1.897383   -0.056755
C         1.283286    2.464352   -0.108087
C         1.325780    3.861039   -0.192461
C         0.124293    4.570429   -0.223683
C        -1.110269    3.921526   -0.171300
AU        0.014207   -0.169196    0.013050
C         2.079668    0.115513   -0.005280
C         2.398614    1.504353   -0.066082
C         3.739627    1.911325   -0.078756
C         4.759296    0.967467   -0.024816
C         4.479582   -0.408529    0.040766
C         3.129955   -0.801865    0.047084
C         5.631896   -1.414843    0.102773
C         6.494946   -1.117125    1.348254
C        -5.555801   -1.269565    0.119909
C        -5.401996   -2.109664    1.406848
C         5.139166   -2.867036    0.191377
C         6.496059   -1.268937   -1.168519
C        -6.948359   -0.622870    0.122050
C        -5.445511   -2.195980   -1.110601
H        -0.104509   -2.032653   -0.241221
H         2.279834    4.379857   -0.235256
H         0.150902    5.656577   -0.292962
H        -2.039046    4.485659   -0.197864
H         3.996231    2.969705   -0.127192
H         5.792164    1.313342   -0.032357
H         2.891910   -1.863099    0.096707
H        -2.875219   -1.741050    0.097467
H        -5.688204    1.526282    0.013211
H        -3.842077    3.128962   -0.075578
H         6.004274   -3.541359    0.237553
H         4.533510   -3.036753    1.091646
H         4.542067   -3.149136   -0.685971
H         7.333632   -1.979419   -1.136246
H         5.901130   -1.477554   -2.067961
H         6.911263   -0.257603   -1.260547
H         7.330485   -1.828508    1.403388
H         6.913773   -0.103581    1.317945
H         5.898615   -1.213663    2.265547
H        -6.198797   -2.864519    1.458606
H        -4.437425   -2.631480    1.434671
H        -5.471769   -1.471768    2.298160
H        -6.240226   -2.954192   -1.081034
H        -5.550676   -1.621083   -2.040526
H        -4.479945   -2.715865   -1.138030
```

H	-7.714179	-1.408417	0.165022
H	-7.090245	0.032029	0.992029
H	-7.123336	-0.033409	-0.787911
O	-0.313418	-3.028184	0.193308
O	-1.505408	-3.465536	-0.195661

[(CNC)Au-H] - O2 TSI triplet H-abstraction

57

C	-3.064774	0.695962	-0.330728
C	-1.973990	-0.160490	-0.219745
C	-2.233028	-1.552758	-0.009698
C	-3.552111	-2.007985	0.070523
C	-4.619759	-1.115634	-0.036920
C	-4.399892	0.252984	-0.233645
C	-1.081511	-2.461647	0.128386
N	0.115752	-1.858593	0.021152
C	1.334240	-2.417023	0.143933
C	1.369522	-3.796372	0.382529
C	0.161682	-4.489615	0.486275
C	-1.069247	-3.841996	0.366928
AU	0.078735	0.220855	-0.282614
C	2.136487	-0.089677	-0.203955
C	2.450502	-1.464052	0.018248
C	3.789531	-1.862718	0.129298
C	4.812122	-0.925825	0.028345
C	4.537245	0.435566	-0.186513
C	3.190160	0.820920	-0.301841
C	5.691624	1.437102	-0.280414
C	6.614740	1.034282	-1.450082
C	-5.538309	1.271710	-0.325671
C	-5.464619	1.992218	-1.688832
C	5.203499	2.873471	-0.518879
C	6.491306	1.407424	1.040286
C	-6.920242	0.616633	-0.192315
C	-5.375577	2.305681	0.810472
H	-0.840895	1.450828	1.255841
H	2.318999	-4.314985	0.488428
H	0.180462	-5.562241	0.672191
H	-2.000073	-4.395193	0.462148
H	4.041693	-2.909519	0.300020
H	5.843047	-1.264862	0.122624
H	2.954454	1.870466	-0.469145
H	-2.878461	1.760210	-0.480070
H	-5.632647	-1.503634	0.041277
H	-3.761516	-3.066682	0.224770
H	6.069440	3.545840	-0.577407
H	4.645586	2.962285	-1.460477
H	4.560738	3.225396	0.298838
H	7.329115	2.116393	0.987651
H	5.851922	1.691250	1.887042
H	6.902061	0.410231	1.241660
H	7.454367	1.739022	-1.525546
H	7.028177	0.027655	-1.310747
H	6.064976	1.049439	-2.400799
H	-6.270243	2.735674	-1.764085
H	-4.508333	2.513947	-1.818749
H	-5.577902	1.275588	-2.513333
H	-6.179316	3.052848	0.755176

H	-5.426338	1.814821	1.791617
H	-4.415243	2.831660	0.743727
H	-7.697517	1.388761	-0.263729
H	-7.101257	-0.116346	-0.989805
H	-7.037757	0.111543	0.775611
O	-1.095956	2.054940	2.048489
O	-1.558885	3.188863	1.519338

[(CNC)Au-H] - O2 INT open shell singlet H-abstraction

57

C	-3.115351	-0.666546	0.009120
C	-2.065615	0.229950	-0.060961
C	-2.331893	1.626435	-0.073139
C	-3.656758	2.071180	-0.024706
C	-4.702281	1.147692	0.046185
C	-4.456875	-0.232845	0.069689
C	-1.167189	2.511972	-0.121300
N	0.023965	1.856781	-0.112102
C	1.248723	2.445626	-0.152707
C	1.291575	3.840561	-0.205963
C	0.096125	4.558332	-0.216440
C	-1.135716	3.907516	-0.172595
AU	-0.021073	-0.145459	-0.106003
C	2.049036	0.121460	-0.104644
C	2.371284	1.503414	-0.123985
C	3.715348	1.899834	-0.085526
C	4.721055	0.942029	-0.013739
C	4.427432	-0.433036	0.024846
C	3.075316	-0.814395	-0.027191
C	5.565094	-1.450638	0.138840
C	6.339727	-1.186647	1.448864
C	-5.573535	-1.273435	0.175314
C	-5.372710	-2.081567	1.476687
C	5.055205	-2.899552	0.161143
C	6.517058	-1.286150	-1.064764
C	-6.970091	-0.636521	0.203999
C	-5.492107	-2.228789	-1.034314
H	0.403744	-2.431960	-1.004216
H	2.249246	4.352741	-0.240303
H	0.125859	5.645508	-0.260397
H	-2.065269	4.470330	-0.181314
H	3.983905	2.956123	-0.099978
H	5.757042	1.276221	0.023379
H	2.821391	-1.873472	0.008019
H	-2.854898	-1.730698	0.022456
H	-5.722423	1.522495	0.088798
H	-3.886608	3.136779	-0.034205
H	5.909967	-3.583262	0.244520
H	4.394054	-3.085315	1.017945
H	4.511358	-3.154057	-0.758356
H	7.344039	-2.005427	-0.988068
H	5.985799	-1.470487	-2.008152
H	6.947297	-0.277810	-1.105941
H	7.163895	-1.906390	1.548429
H	6.766365	-0.176075	1.469270
H	5.678431	-1.296554	2.318764
H	-6.157242	-2.845397	1.569824

```
H        -4.399324   -2.587676    1.486385
H        -5.425131   -1.423494    2.354497
H        -6.278176   -2.993081   -0.961573
H        -5.631207   -1.678741   -1.974855
H        -4.522672   -2.740370   -1.077449
H        -7.729215   -1.426288    0.278081
H        -7.095059    0.029791    1.067863
H        -7.172049   -0.061165   -0.709535
O         0.086165   -2.226613   -0.099601
O        -1.135620   -3.029049   -0.004818
```

[(CNC)Au-H] - O2 PC triplet H-abstraction

57

```
C        -3.135696    0.722364    0.086136
C        -2.093992   -0.188853    0.066560
C        -2.355169   -1.580384    0.066874
C        -3.684286   -2.014736    0.084602
C        -4.726573   -1.087636    0.104814
C        -4.479508    0.291476    0.107786
C        -1.194924   -2.470219    0.057651
N        -0.005262   -1.813790    0.073250
C         1.207908   -2.427473    0.056680
C         1.244504   -3.821590    0.027542
C         0.044250   -4.530842    0.014938
C        -1.180241   -3.865797    0.030053
AU       -0.040225    0.206656    0.083258
C         2.024185   -0.120239    0.107878
C         2.335292   -1.496042    0.068217
C         3.683939   -1.876583    0.026515
C         4.682275   -0.908153    0.024647
C         4.382049    0.464356    0.068443
C         3.027430    0.839605    0.115881
C         5.509980    1.499217    0.055512
C         6.304125    1.348153   -1.260542
C        -5.597973    1.336908    0.133969
C        -5.488607    2.224681   -1.124545
C         4.983219    2.939404    0.144842
C         6.445642    1.244910    1.256509
C        -6.992652    0.694877    0.159742
C        -5.437710    2.213598    1.395301
H         1.359224    1.816153   -1.724692
H         2.199210   -4.340147    0.014493
H         0.064164   -5.618667   -0.008724
H        -2.115280   -4.419013    0.016614
H         3.958037   -2.930472   -0.013023
H         5.720828   -1.233468   -0.013949
H         2.757334    1.892154    0.155061
H        -2.912245    1.788059    0.085482
H        -5.748507   -1.458346    0.120332
H        -3.915830   -3.079490    0.088440
H         5.831032    3.636401    0.134104
H         4.334492    3.190185   -0.704803
H         4.420937    3.109883    1.072671
H         7.264485    1.977369    1.251817
H         5.898324    1.345240    2.203252
H         6.888030    0.241777    1.221005
H         7.124329    2.078711   -1.287276
H         6.737712    0.345110   -1.357351
```

H	5.654694	1.526051	-2.128093
H	-6.283282	2.983304	-1.115722
H	-4.524015	2.745176	-1.170322
H	-5.595990	1.622138	-2.036384
H	-6.232841	2.971183	1.426093
H	-5.506839	1.602660	2.305254
H	-4.472534	2.734966	1.406106
H	-7.754461	1.485093	0.179134
H	-7.172223	0.077562	-0.730471
H	-7.135249	0.069447	1.050946
O	0.815241	2.622656	-1.815327
O	-0.074847	2.113026	0.072344

[(CNC)Au-H] - O2 TSII open shell singlet H-abstraction

57

C	-3.072457	0.765181	-0.019797
C	-2.022242	-0.137832	0.012751
C	-2.303531	-1.531988	0.004020
C	-3.633825	-1.964768	-0.008980
C	-4.672621	-1.033576	-0.019787
C	-4.418319	0.346019	-0.032250
C	-1.146765	-2.424159	-0.019765
N	0.055232	-1.775977	-0.038793
C	1.275121	-2.388817	-0.065648
C	1.296654	-3.784266	-0.081347
C	0.094528	-4.489425	-0.064990
C	-1.127525	-3.819747	-0.033219
AU	0.026871	0.188603	0.012333
C	2.084944	-0.081211	-0.020697
C	2.404118	-1.462814	-0.067592
C	3.748547	-1.859713	-0.117795
C	4.753402	-0.899868	-0.122509
C	4.460624	0.476845	-0.079620
C	3.109297	0.860051	-0.028824
C	5.605116	1.493685	-0.098468
C	6.411460	1.315840	-1.403690
C	-5.531503	1.395557	-0.076495
C	-5.384938	2.221473	-1.373685
C	5.101497	2.943182	-0.031701
C	6.527410	1.241047	1.113687
C	-6.930839	0.764263	-0.054167
C	-5.397408	2.331546	1.144016
H	-0.881931	2.531934	0.784813
H	2.249781	-4.305886	-0.101688
H	0.110436	-5.577551	-0.074759
H	-2.064862	-4.369396	-0.017617
H	4.015691	-2.915855	-0.156841
H	5.790024	-1.231481	-0.164691
H	2.847052	1.916477	-0.001406
H	-2.855217	1.834855	-0.060688
H	-5.696684	-1.399064	-0.028786
H	-3.871886	-3.028456	-0.016752
H	5.960281	3.626899	-0.046398
H	4.461536	3.191486	-0.888706
H	4.535816	3.134554	0.889938
H	7.359371	1.958797	1.106486
H	5.974340	1.363434	2.054631

H	6.952229	0.229705	1.095824
H	7.241072	2.035779	-1.432683
H	6.834430	0.306637	-1.482850
H	5.773103	1.488899	-2.280522
H	-6.170980	2.987805	-1.420969
H	-4.413190	2.728594	-1.421713
H	-5.477345	1.576020	-2.257368
H	-6.189832	3.092223	1.120597
H	-5.489104	1.765436	2.080621
H	-4.431125	2.850909	1.152562
H	-7.688082	1.558613	-0.083600
H	-7.096150	0.111652	-0.921563
H	-7.095547	0.176578	0.858863
O	0.024416	2.289273	0.500997
O	-0.096534	2.359345	-1.018671

[(CNC)Au-H] - O2 PC open shell singlet H-abstraction

57

C	-3.080729	0.633753	-0.204715
C	-2.003628	-0.249163	-0.221604
C	-2.260810	-1.639393	-0.076601
C	-3.580703	-2.095232	0.052507
C	-4.633701	-1.187760	0.064585
C	-4.406654	0.195326	-0.048593
AU	0.052974	0.120896	-0.312605
N	0.091624	-1.875283	-0.091049
C	1.311695	-2.473363	-0.038228
C	1.352176	-3.864449	0.080647
C	0.154784	-4.576122	0.141276
C	-1.074025	-3.920075	0.088794
C	-1.097482	-2.527884	-0.029081
C	2.437408	-1.537064	-0.103828
C	2.113662	-0.159131	-0.242067
C	3.134722	0.777227	-0.265104
C	4.490890	0.408014	-0.149572
C	4.788177	-0.957657	-0.035014
C	3.779156	-1.921628	-0.012403
C	5.568772	1.494632	-0.133155
C	5.483573	2.317176	-1.436791
C	-5.587462	1.167711	0.016161
C	-6.570208	0.851854	-1.131212
C	-6.305531	0.992253	1.372009
C	-5.145010	2.632982	-0.110467
C	5.317637	2.422802	1.075896
C	6.984797	0.913209	-0.013836
O	0.277165	2.087373	-0.583997
H	-2.005083	-4.478103	0.139508
H	0.180075	-5.660607	0.232134
H	2.308509	-4.378660	0.124539
H	-3.791405	-3.159592	0.157672
H	-5.649010	-1.566000	0.177443
H	-2.868234	1.695332	-0.308121
H	2.871851	1.832285	-0.365934
H	5.821407	-1.286361	0.048299
H	4.050356	-2.972775	0.087775
H	-6.026810	3.284716	-0.054096
H	-4.461812	2.921933	0.699049
H	-4.645528	2.825950	-1.069065

H	-7.428231	1.537081	-1.089631
H	-6.079292	0.972723	-2.106177
H	-6.951981	-0.174658	-1.065159
H	-7.157858	1.682624	1.437764
H	-6.685013	-0.029220	1.499523
H	-5.621342	1.208656	2.203570
H	6.078146	3.215503	1.104929
H	4.331000	2.899298	1.020233
H	5.371377	1.857859	2.016288
H	6.245280	3.109197	-1.430230
H	5.657991	1.677108	-2.312059
H	4.501747	2.792684	-1.551508
H	7.714903	1.733117	-0.005783
H	7.112121	0.342298	0.915566
H	7.226826	0.256466	-0.859987
O	-0.954763	2.842039	-0.850605
H	-1.023445	2.755334	-1.822649

[(PCP)Au-H]+ SOC

71

AU	-0.002583	0.000150	0.167410
H	-0.030963	0.000047	1.798895
C	0.033707	-0.000002	-1.919455
C	-1.173188	0.000219	-2.649954
C	-1.139035	0.000270	-4.050033
C	0.082578	0.000104	-4.723513
C	1.280006	-0.000114	-4.007995
C	1.265245	-0.000195	-2.607615
C	-2.510733	0.000530	-1.944792
H	-2.070867	0.000468	-4.616111
H	0.101493	0.000155	-5.812829
H	2.231031	-0.000225	-4.541255
C	2.577462	-0.000546	-1.856469
P	2.320638	-0.000050	-0.024755
H	3.182698	-0.876155	-2.125501
H	3.183470	0.874403	-2.125915
P	-2.317759	0.000069	-0.105126
H	-3.107019	-0.874366	-2.235231
H	-3.106258	0.876119	-2.234709
C	-3.071037	-1.599330	0.504131
C	-3.070947	1.599068	0.505200
C	-2.227070	2.708088	-0.154596
C	-4.542082	1.740331	0.089273
C	-2.933806	1.708091	2.030737
C	-4.541879	-1.740499	0.087187
C	-2.226668	-2.707858	-0.155870
C	-2.934960	-1.709267	2.029724
H	-3.206713	-2.730636	2.329072
H	-1.906828	-1.518043	2.359949
H	-3.605622	-1.020037	2.552094
H	-4.881557	-2.750994	0.353230
H	-5.184659	-1.025443	0.610199
H	-4.688805	-1.617591	-0.992647
H	-2.628450	-3.681309	0.155218
H	-2.253013	-2.667716	-1.251207
H	-1.178493	-2.654822	0.165979
H	-3.206260	2.728963	2.331147
H	-3.603269	1.017726	2.553065

H	-1.905231	1.517505	2.359951
H	-2.628809	3.681305	0.157282
H	-1.178737	2.654954	0.166733
H	-2.253966	2.668601	-1.249944
H	-4.881668	2.750599	0.356279
H	-4.689599	1.618276	-0.990579
H	-5.184593	1.024859	0.612086
C	3.052481	1.599280	0.610361
C	3.052252	-1.599046	0.611437
C	2.231991	-2.708055	-0.077500
C	4.537006	-1.740013	0.246935
C	2.862117	-1.708068	2.131318
C	4.537210	1.739786	0.245580
C	2.232359	2.707928	-0.079343
C	2.862399	1.709380	2.130179
H	3.123583	-2.728977	2.441083
H	3.513271	-1.017916	2.676619
H	1.822729	-1.517122	2.424537
H	4.867170	-2.750400	0.525036
H	4.722366	-1.617140	-0.826974
H	5.160634	-1.024838	0.792503
H	2.622126	-3.681279	0.248857
H	1.172919	-2.654590	0.206297
H	2.297811	-2.669010	-1.171205
H	4.867496	2.750375	0.522789
H	5.160873	1.025005	0.791638
H	4.722425	1.615998	-0.828253
H	3.123923	2.730495	2.439218
H	1.823020	1.518685	2.423589
H	3.513546	1.019580	2.675926
H	2.622680	3.681320	0.246286
H	2.298104	2.668070	-1.173022
H	1.173294	2.654854	0.204539

O2 SOC

2

O	0.000000	0.000000	-0.041616
O	0.000000	0.000000	1.181616

[(PCP)Au-H]+ - O2 RC SOC

73

C	1.394688	-3.924130	0.268708
C	1.329811	-2.543605	0.046193
C	0.103292	-1.869197	0.204222
C	-1.050695	-2.584911	0.578080
C	-0.973457	-3.967921	0.780446
C	0.246088	-4.630294	0.629905
C	2.557303	-1.783682	-0.400875
P	2.322966	0.032647	-0.160852
C	3.109674	0.869867	-1.632252
C	2.180459	0.559427	-2.822323
AU	-0.005319	0.202893	-0.119240
P	-2.304730	-0.178304	0.022943
C	-3.181639	0.978486	1.197280
C	-2.226669	1.141294	2.396145
C	-2.353366	-1.852070	0.802368

C	-3.036047	-0.336872	-1.690267
C	-4.555282	-0.546494	-1.637669
C	-2.686646	0.914092	-2.512711
C	-2.360289	-1.559712	-2.339224
C	3.021791	0.471409	1.517528
C	4.555608	0.427291	1.519289
C	2.515783	1.862563	1.931603
C	2.466245	-0.569663	2.507601
C	3.154928	2.387341	-1.409533
C	4.516023	0.318676	-1.917663
C	-3.392878	2.344264	0.530710
C	-4.519975	0.395287	1.678366
O	0.017898	4.335523	0.429542
O	-0.177759	4.319347	1.637951
H	-0.088356	1.811595	-0.375676
H	-1.864457	-4.527519	1.065861
H	0.301758	-5.705674	0.795654
H	2.342457	-4.450464	0.152863
H	3.469972	-2.124463	0.105142
H	2.723486	-1.939443	-1.476511
H	-2.510446	-1.691691	1.878730
H	-3.222521	-2.419217	0.444599
H	-3.744935	3.053965	1.291591
H	-2.459407	2.735036	0.107730
H	-4.150064	2.301791	-0.259977
H	-4.955654	1.090759	2.408788
H	-5.238738	0.273795	0.862601
H	-4.394482	-0.571839	2.179192
H	-2.709042	1.787125	3.141985
H	-1.995807	0.185705	2.883298
H	-1.284633	1.614474	2.092097
H	-3.055170	0.769905	-3.537278
H	-3.147551	1.822339	-2.113058
H	-1.601864	1.070487	-2.557237
H	-2.725129	-1.641232	-3.371942
H	-1.268961	-1.454408	-2.369591
H	-2.600802	-2.495396	-1.822196
H	-4.913045	-0.757710	-2.654782
H	-4.836322	-1.397124	-1.004388
H	-5.079287	0.346937	-1.281093
H	2.875411	2.075494	2.947301
H	2.877951	2.656311	1.271553
H	1.419739	1.897306	1.942119
H	4.906727	0.567771	2.550774
H	4.944636	-0.535466	1.165025
H	4.988353	1.228140	0.909952
H	2.811073	-0.298119	3.514421
H	1.369517	-0.582197	2.513436
H	2.820293	-1.584032	2.292571
H	4.896040	0.802220	-2.828033
H	5.219818	0.531223	-1.107608
H	4.508277	-0.762648	-2.098257
H	3.463036	2.869191	-2.347217
H	2.170006	2.783181	-1.132319
H	3.880918	2.667168	-0.638100
H	2.615745	1.000782	-3.729101
H	2.068199	-0.517720	-2.998674
H	1.184654	0.995580	-2.673656

[(PCP)Au-H]+ - O2 TSI SOC

C	1.133071	3.966358	0.073415
C	1.160285	2.567301	0.160108
C	-0.034827	1.886355	-0.078882
C	-1.239620	2.517427	-0.391772
C	-1.236841	3.918035	-0.453093
C	-0.057853	4.629178	-0.225730
C	2.418604	1.827601	0.542960
P	2.313627	0.033492	0.110779
C	3.186514	-0.878472	1.486672
C	2.220538	-0.824506	2.685666
AU	-0.018608	-0.243303	0.045179
O	-0.265523	-3.357685	0.291816
O	0.804708	-4.104600	-0.055486
C	-2.483341	1.719616	-0.696727
P	-2.358076	-0.012340	-0.062982
C	-3.220722	-1.079616	-1.331745
C	-2.255960	-1.147518	-2.530844
C	-3.074839	-0.032872	1.663227
C	-4.602262	0.110283	1.643116
C	-2.654205	-1.336133	2.361817
C	-2.446523	1.155961	2.413814
C	3.018877	-0.176951	-1.606927
C	4.547052	-0.040402	-1.609982
C	2.585749	-1.547081	-2.153702
C	2.392120	0.924953	-2.480635
C	3.415582	-2.339764	1.080398
C	4.517617	-0.206565	1.858561
C	-3.446632	-2.491797	-0.775719
C	-4.556726	-0.461065	-1.774484
H	-0.114092	-2.387252	-0.062126
H	-2.160937	4.446840	-0.686643
H	-0.067293	5.716658	-0.283073
H	2.048223	4.532575	0.247263
H	3.314250	2.278540	0.097019
H	2.554059	1.876500	1.632632
H	-2.608880	1.642661	-1.785975
H	-3.389263	2.204839	-0.312125
H	-3.818451	-3.128826	-1.589451
H	-2.520738	-2.942412	-0.398498
H	-4.195737	-2.503415	0.023449
H	-4.996365	-1.112833	-2.541824
H	-5.273173	-0.384265	-0.951121
H	-4.429465	0.531559	-2.221023
H	-2.733773	-1.723846	-3.334226
H	-2.012432	-0.155820	-2.932567
H	-1.318397	-1.652876	-2.263917
H	-3.015385	-1.308301	3.398670
H	-3.072149	-2.226457	1.882740
H	-1.561235	-1.438795	2.388240
H	-2.803185	1.127942	3.452172
H	-1.351117	1.096862	2.431216
H	-2.733486	2.122666	1.985274
H	-4.956056	0.202815	2.679100
H	-4.927787	1.006950	1.101474
H	-5.091357	-0.765337	1.202608
H	2.954431	-1.645834	-3.183597
H	2.981215	-2.382216	-1.568877
H	1.491010	-1.633885	-2.178815
H	4.896727	-0.069908	-2.651100

H	4.878201	0.911433	-1.176466
H	5.034342	-0.861195	-1.072764
H	2.734532	0.774810	-3.513152
H	1.296046	0.876569	-2.477891
H	2.694447	1.931062	-2.169080
H	4.962618	-0.769403	2.690731
H	5.232788	-0.213490	1.030296
H	4.383504	0.827426	2.196492
H	3.776438	-2.892074	1.958738
H	2.493185	-2.825390	0.733634
H	4.175018	-2.432419	0.296021
H	2.700590	-1.311076	3.545425
H	1.973298	0.203099	2.981376
H	1.286415	-1.360069	2.469927

[(PCP)Au-H]+ - O2 INT SOC

73

C	1.360283	-2.479819	0.063242
C	0.131122	-1.825541	0.262064
C	-1.023746	-2.543504	0.620102
C	-0.930671	-3.929523	0.790752
C	0.288459	-4.582764	0.621481
C	1.426185	-3.863893	0.260592
AU	0.012400	0.198301	-0.051586
P	-2.316727	-0.168492	0.088167
C	-3.007636	-0.293015	-1.642675
C	-2.580709	0.957831	-2.429693
C	-2.336775	-1.842791	0.845448
C	2.577951	-1.726934	-0.403606
P	2.374619	0.077697	-0.121315
C	3.094177	0.475670	1.555436
C	2.727670	1.917133	1.945435
C	3.134262	0.939099	-1.593390
C	3.261514	2.445664	-1.325570
C	-3.221628	0.946078	1.281805
C	-3.448055	2.333447	0.666231
C	-4.557393	0.316549	1.709206
C	-2.279139	1.080466	2.495383
C	-2.363558	-1.530377	-2.293162
C	-4.534651	-0.440037	-1.631604
C	4.505736	0.335335	-1.937510
C	2.154634	0.703183	-2.759316
C	2.428307	-0.497687	2.546444
C	4.615681	0.274000	1.563172
H	-0.871745	2.526555	-0.921121
H	-1.821780	-4.494122	1.063103
H	0.350796	-5.660286	0.765310
H	2.376949	-4.377128	0.119639
H	3.497523	-2.093645	0.069093
H	2.705569	-1.867044	-1.486316
H	-2.504209	-1.698968	1.921843
H	-3.187045	-2.426879	0.471908
H	-3.955566	2.957921	1.414529
H	-2.490228	2.822863	0.440232
H	-4.088109	2.301480	-0.221810
H	-5.003826	0.967311	2.473637

H	-5.267968	0.237318	0.881203
H	-4.429851	-0.675324	2.157976
H	-2.793314	1.676040	3.262469
H	-2.029702	0.111566	2.946172
H	-1.358322	1.608415	2.208398
H	-2.992761	0.894634	-3.445375
H	-2.947752	1.888496	-1.983383
H	-1.487344	1.008098	-2.527354
H	-2.692640	-1.574270	-3.339878
H	-1.267919	-1.475463	-2.286443
H	-2.666711	-2.463822	-1.806336
H	-4.873091	-0.619428	-2.661135
H	-4.862485	-1.290486	-1.021425
H	-5.032174	0.466570	-1.271092
H	2.951305	2.048551	3.013170
H	3.318213	2.651411	1.389150
H	1.663384	2.143951	1.778282
H	4.976073	0.414323	2.591558
H	4.909294	-0.734178	1.244776
H	5.126581	1.007937	0.930714
H	2.827594	-0.284300	3.546732
H	1.340177	-0.362455	2.581347
H	2.638969	-1.548315	2.316725
H	4.891565	0.855069	-2.825343
H	5.233184	0.467183	-1.130737
H	4.443904	-0.731318	-2.181924
H	3.537803	2.939388	-2.267116
H	2.318794	2.880631	-0.975354
H	4.048299	2.661578	-0.594951
H	2.597139	1.115797	-3.675771
H	1.957991	-0.361699	-2.937869
H	1.199210	1.214406	-2.588814
O	-0.036310	2.327541	-0.449056
O	-0.202961	3.050980	0.829579

[(PCP)Au-H]+ - O2 TSII SOC

73

C	1.187688	2.456824	0.127677
C	0.000973	1.751673	-0.146941
C	-1.183677	2.434148	-0.479146
C	-1.158749	3.831392	-0.557114
C	0.018463	4.536189	-0.317335
C	1.182940	3.852444	0.024773
AU	-0.005834	-0.282904	0.006360
P	-2.350490	-0.020358	-0.094851
C	-3.035241	-0.011187	1.647426
C	-2.562685	-1.278596	2.378565
C	-2.459034	1.688080	-0.757851
C	2.431790	1.738661	0.571702
P	2.345211	-0.034251	0.101668
C	3.119650	-0.190041	-1.596352
C	2.904481	-1.606833	-2.151663
C	3.161309	-0.989294	1.484058
C	3.375356	-2.450312	1.062980
C	-3.230782	-1.102121	-1.339621
C	-3.316784	-2.545105	-0.824463
C	-4.630025	-0.548030	-1.654850
C	-2.359759	-1.071567	-2.611147

C	-2.433203	1.216826	2.354493
C	-4.566760	0.080428	1.656265
C	4.496515	-0.340565	1.885762
C	2.183823	-0.931017	2.673968
C	2.376407	0.817198	-2.494510
C	4.615353	0.153847	-1.552498
H	-0.860227	-2.772428	0.738458
H	-2.075315	4.363592	-0.809703
H	0.026372	5.622597	-0.388679
H	2.103037	4.400805	0.224763
H	3.342699	2.207329	0.180033
H	2.506511	1.772786	1.667993
H	-2.616234	1.598023	-1.841490
H	-3.337224	2.207896	-0.355023
H	-3.808507	-3.157243	-1.592878
H	-2.311937	-2.962874	-0.675723
H	-3.909300	-2.630964	0.092618
H	-5.048483	-1.146094	-2.476107
H	-5.313858	-0.622255	-0.805066
H	-4.600696	0.495080	-1.991729
H	-2.850041	-1.695015	-3.371358
H	-2.260111	-0.062477	-3.029481
H	-1.364378	-1.484319	-2.404607
H	-2.966861	-1.265308	3.399459
H	-2.910915	-2.200418	1.899960
H	-1.468370	-1.304657	2.459977
H	-2.759269	1.196436	3.402964
H	-1.336682	1.201178	2.340510
H	-2.772958	2.161388	1.915542
H	-4.897683	0.219297	2.694529
H	-4.935261	0.933429	1.073646
H	-5.033971	-0.835179	1.278649
H	3.143025	-1.593239	-3.223987
H	3.564504	-2.335976	-1.671847
H	1.867263	-1.941059	-2.012446
H	4.991583	0.171148	-2.584880
H	4.813043	1.139387	-1.113287
H	5.189930	-0.600430	-1.004603
H	2.800318	0.741945	-3.504721
H	1.305203	0.590344	-2.558466
H	2.492147	1.853208	-2.156878
H	4.915288	-0.919182	2.720731
H	5.228029	-0.350771	1.072320
H	4.371339	0.692123	2.231448
H	3.672517	-3.025218	1.950615
H	2.460834	-2.896303	0.655988
H	4.177638	-2.543764	0.323387
H	2.664548	-1.407405	3.539113
H	1.930616	0.097171	2.961941
H	1.257960	-1.475572	2.457697
O	0.032542	-2.447306	0.497046
O	0.041013	-2.602276	-1.010840

[(PCP)Au-H]+ - O2 PC SOC

73

C	1.394783	-3.849344	0.097333
C	1.340176	-2.461170	-0.070979
C	0.125171	-1.787866	0.158512

C	-1.018165	-2.499866	0.568643
C	-0.943019	-3.889507	0.714934
C	0.257286	-4.559034	0.479832
C	2.567230	-1.700960	-0.506123
P	2.380963	0.085584	-0.108188
C	3.200331	1.026386	-1.494376
C	2.273009	0.845431	-2.712710
AU	0.028421	0.259389	-0.053370
O	-0.130758	2.852482	-1.482561
O	-0.049284	2.359158	-0.096218
C	-2.299243	-1.772075	0.891193
P	-2.302230	-0.110229	0.102636
C	-3.159171	1.047142	1.291147
C	-2.170405	1.238253	2.458377
C	-3.080811	-0.301731	-1.587631
C	-4.599820	-0.492842	-1.483157
C	-2.741567	0.926376	-2.446036
C	-2.440163	-1.542753	-2.235622
C	3.059762	0.374886	1.609344
C	4.593671	0.331902	1.621011
C	2.544662	1.728781	2.125326
C	2.499795	-0.743127	2.507358
C	3.291227	2.516201	-1.134257
C	4.591146	0.454919	-1.813093
C	-3.409573	2.403947	0.618651
C	-4.471855	0.444732	1.815775
H	-1.097771	2.946894	-1.599186
H	-1.827824	-4.445904	1.023188
H	0.307998	-5.640140	0.601480
H	2.334532	-4.374133	-0.073713
H	3.485305	-2.106940	-0.062735
H	2.688382	-1.769288	-1.596499
H	-2.370169	-1.605682	1.975574
H	-3.189278	-2.345183	0.602853
H	-3.804635	3.097840	1.373176
H	-2.474159	2.827475	0.232559
H	-4.147030	2.337555	-0.188410
H	-4.890963	1.135857	2.560151
H	-5.216375	0.313138	1.025320
H	-4.316475	-0.519777	2.313414
H	-2.650002	1.868270	3.219531
H	-1.896056	0.290969	2.939154
H	-1.257356	1.745767	2.124142
H	-3.179472	0.787854	-3.443486
H	-3.150592	1.854427	-2.032471
H	-1.656753	1.034920	-2.569866
H	-2.833397	-1.630286	-3.257328
H	-1.348810	-1.456170	-2.298327
H	-2.683723	-2.467743	-1.701640
H	-4.989189	-0.721071	-2.484833
H	-4.869354	-1.327791	-0.824762
H	-5.103892	0.413030	-1.129731
H	2.891526	1.860331	3.159070
H	2.911278	2.572016	1.533261
H	1.448215	1.764810	2.126375
H	4.934368	0.398173	2.663471
H	4.985753	-0.603486	1.202661
H	5.031290	1.174300	1.074746
H	2.840427	-0.553671	3.534026
H	1.403250	-0.756606	2.510984

H	2.856136	-1.736562	2.212608
H	4.994123	1.006247	-2.674093
H	5.293429	0.572712	-0.982608
H	4.552222	-0.604931	-2.090956
H	3.618849	3.068546	-2.025576
H	2.318803	2.912400	-0.820651
H	4.026817	2.699151	-0.342928
H	2.738294	1.341159	-3.575962
H	2.130724	-0.209840	-2.977791
H	1.294414	1.308980	-2.540598

[(PCP)Pd-H] SOC

71

PD	-0.002072	0.000195	0.197818
H	-0.029606	0.000250	1.841652
C	0.033458	-0.000015	-1.876113
C	-1.167423	0.000195	-2.630648
C	-1.136986	-0.000085	-4.032690
C	0.082494	-0.000558	-4.713288
C	1.277755	-0.000708	-3.990992
C	1.259683	-0.000439	-2.588754
C	-2.505443	0.000871	-1.924114
H	-2.070992	0.000089	-4.598345
H	0.101351	-0.000784	-5.803565
H	2.230746	-0.001034	-4.524057
C	2.572571	-0.000617	-1.836499
P	2.262898	-0.000054	-0.001502
H	3.179567	-0.875787	-2.106187
H	3.180057	0.874075	-2.106638
P	-2.259536	0.000262	-0.079319
H	-3.103646	-0.873499	-2.215085
H	-3.102319	0.876337	-2.214505
C	-3.100652	-1.584627	0.496579
C	-3.100812	1.584522	0.497992
C	-2.269529	2.708105	-0.153475
C	-4.569357	1.715272	0.075960
C	-2.973736	1.711013	2.023334
C	-4.569144	-1.715084	0.074304
C	-2.269255	-2.707479	-0.156015
C	-2.973616	-1.712656	2.021813
H	-3.249770	-2.734462	2.320704
H	-1.942907	-1.516840	2.344156
H	-3.640928	-1.020297	2.546684
H	-4.921098	-2.730263	0.310633
H	-5.211914	-1.009457	0.611047
H	-4.711321	-1.559934	-1.002686
H	-2.654348	-3.679976	0.182975
H	-2.323387	-2.684842	-1.251564
H	-1.212567	-2.624062	0.131962
H	-3.250301	2.732374	2.323369
H	-3.640666	1.017771	2.547496
H	-1.942919	1.515278	2.345385
H	-2.654684	3.680219	0.186546
H	-1.212824	2.624475	0.134383
H	-2.323721	2.686613	-1.249047
H	-4.921414	2.730138	0.313466
H	-4.711575	1.561352	-1.001198
H	-5.212017	1.008960	0.611937

C	3.082617	1.584875	0.604109
C	3.082381	-1.584621	0.605351
C	2.273462	-2.707662	-0.074659
C	4.564645	-1.716042	0.234924
C	2.901726	-1.710456	2.125350
C	4.564737	1.715912	0.232992
C	2.273606	2.707476	-0.076523
C	2.902454	1.711912	2.124088
H	3.166438	-2.731952	2.435287
H	3.550639	-1.017665	2.672248
H	1.860378	-1.513371	2.410825
H	4.908115	-2.730683	0.485591
H	4.744499	-1.563136	-0.836736
H	5.188170	-1.009193	0.792431
H	2.645170	-3.680075	0.279263
H	1.207327	-2.622510	0.175690
H	2.366349	-2.686746	-1.167633
H	4.908387	2.730699	0.482822
H	5.188439	1.009389	0.790706
H	4.744136	1.562260	-0.838643
H	3.167163	2.733694	2.433083
H	1.861215	1.514983	2.410063
H	3.551619	1.019662	2.671400
H	2.645618	3.680127	0.276422
H	2.366041	2.685613	-1.169517
H	1.207559	2.622708	0.174319

[(PCP)Pd-H] - O2 RC SOC

73

C	1.198131	-2.588683	0.282868
C	-0.013834	-1.891421	0.060636
C	-1.233339	-2.573661	0.287808
C	-1.234576	-3.878065	0.801223
C	-0.028003	-4.530052	1.069298
C	1.185431	-3.892951	0.796396
PD	-0.002384	0.126946	-0.438350
P	-2.255341	-0.060626	-0.208269
C	-3.326597	0.480443	-1.656486
C	-3.402453	2.013066	-1.687519
C	-2.534381	-1.892374	-0.074023
C	2.505754	-1.923130	-0.084399
P	2.248798	-0.087854	-0.213480
C	2.843490	0.593362	1.441991
C	2.363737	2.046933	1.583445
C	3.324062	0.444173	-1.662052
C	3.420300	1.975763	-1.687517
C	-2.838654	0.631605	1.446635
C	-2.344742	2.080952	1.582368
C	-4.356790	0.560936	1.645479
C	-2.139087	-0.216428	2.524934
C	-2.556261	-0.000789	-2.901664
C	-4.735418	-0.129816	-1.662102
C	4.724490	-0.184991	-1.672834
C	2.544921	-0.022089	-2.907430
C	2.139528	-0.251304	2.520043
C	4.361518	0.507767	1.635859
H	0.006669	1.727947	-0.800231
H	-2.180282	-4.387944	0.994988

H	-0.033417	-5.539935	1.480579
H	2.125582	-4.414406	0.986417
H	3.324024	-2.179557	0.602251
H	2.821364	-2.267801	-1.080640
H	-3.351876	-2.136768	0.617926
H	-2.860120	-2.235540	-1.067496
H	-2.563049	2.440867	2.598488
H	-1.262935	2.137369	1.408207
H	-2.833270	2.755276	0.871494
H	-4.596785	0.855723	2.677940
H	-4.888722	1.245776	0.975216
H	-4.748134	-0.452812	1.491148
H	-2.350550	0.221967	3.510553
H	-2.489406	-1.255228	2.532585
H	-1.052780	-0.226014	2.372542
H	-3.841285	2.333607	-2.643545
H	-4.034680	2.406854	-0.882959
H	-2.402499	2.457359	-1.598183
H	-3.100922	0.310761	-3.804862
H	-1.547840	0.433480	-2.925811
H	-2.457003	-1.093935	-2.928581
H	-5.249981	0.173448	-2.585908
H	-4.709222	-1.226359	-1.645679
H	-5.340445	0.211536	-0.816758
H	2.589795	2.402337	2.599465
H	2.855209	2.718444	0.871923
H	1.281706	2.113707	1.414203
H	4.607540	0.798182	2.668144
H	4.742518	-0.509429	1.478351
H	4.897948	1.188764	0.965262
H	2.357556	0.182746	3.506153
H	1.052760	-0.251316	2.370850
H	2.480970	-1.293055	2.524017
H	5.241664	0.115724	-2.596015
H	5.335417	0.143984	-0.826822
H	4.683378	-1.281133	-1.661707
H	3.862031	2.293949	-2.642983
H	2.426446	2.432958	-1.595199
H	4.058764	2.358203	-0.882418
H	3.092072	0.285121	-3.810614
H	2.430806	-1.113687	-2.937885
H	1.542511	0.425985	-2.928127
O	0.028185	4.014641	-0.763862
O	0.026831	4.535911	0.357497

[(PCP)Pd-H] - O2 TSI SOC

73

C	1.215700	2.590112	0.104966
C	-0.002737	1.881043	0.188747
C	-1.223283	2.586747	0.107132
C	-1.216036	3.967145	-0.136721
C	-0.007027	4.652846	-0.274475
C	1.204122	3.970511	-0.138861
PD	0.000134	-0.199277	0.273431
P	-2.268709	0.030296	0.112146
C	-3.332140	-0.766073	1.443724
C	-3.443680	-2.273930	1.179818
C	-2.524987	1.854696	0.338330

C	2.519923	1.861800	0.333835
P	2.268235	0.036439	0.110082
C	2.866946	-0.321962	-1.640495
C	2.398201	-1.729147	-2.044643
C	3.334280	-0.755486	1.442223
C	3.448615	-2.263552	1.180730
C	-2.867502	-0.327384	-1.638598
C	-2.394934	-1.732635	-2.045088
C	-4.385127	-0.199288	-1.808667
C	-2.158569	0.699332	-2.541316
C	-2.541712	-0.547959	2.748736
C	-4.728131	-0.142258	1.583455
C	4.729116	-0.128670	1.579959
C	2.544331	-0.536817	2.747444
C	2.154242	0.701348	-2.544112
C	4.384062	-0.189672	-1.811800
H	0.002462	-2.010527	0.286059
H	-2.160190	4.508493	-0.219000
H	-0.008691	5.724323	-0.475595
H	2.146612	4.514502	-0.222813
H	3.335464	2.244098	-0.294446
H	2.837774	2.011555	1.376205
H	-3.343143	2.235517	-0.287442
H	-2.840553	2.002285	1.381712
H	-2.624095	-1.894208	-3.108525
H	-1.311960	-1.838634	-1.898622
H	-2.888316	-2.521726	-1.468621
H	-4.635147	-0.295339	-2.875597
H	-4.924589	-0.987716	-1.271583
H	-4.759360	0.774603	-1.467605
H	-2.383197	0.459861	-3.590340
H	-2.490904	1.726045	-2.348682
H	-1.071000	0.662010	-2.401063
H	-3.889620	-2.759740	2.059748
H	-4.086711	-2.491632	0.319066
H	-2.457671	-2.722886	1.003907
H	-3.090819	-1.008474	3.582748
H	-1.545880	-1.006478	2.684806
H	-2.410559	0.517310	2.979879
H	-5.242548	-0.614231	2.433535
H	-4.681031	0.934713	1.785847
H	-5.344723	-0.297960	0.692951
H	2.626569	-1.891355	-3.108158
H	2.894659	-2.516017	-1.467792
H	1.315750	-1.838405	-1.896669
H	4.633618	-0.286340	-2.878784
H	4.755757	0.785701	-1.472196
H	4.926138	-0.975892	-1.274111
H	2.378698	0.461058	-3.592984
H	1.066896	0.660930	-2.402901
H	2.483674	1.729325	-2.353234
H	5.244980	-0.598077	2.430583
H	5.345467	-0.284785	0.689362
H	4.680038	0.948576	1.780417
H	3.896134	-2.747036	2.061139
H	2.463367	-2.714704	1.006243
H	4.091467	-2.481388	0.319880
H	3.094983	-0.994828	3.581817
H	2.411135	0.528556	2.976931
H	1.549407	-0.997506	2.684994

O	0.002040	-3.418359	0.427510
O	0.009756	-4.040592	-0.711422

[(PCP)Pd-H] - O2 INT SOC

73

C	1.210502	-2.553723	0.209946
C	-0.001676	-1.833508	0.052831
C	-1.221183	-2.514014	0.296079
C	-1.214111	-3.833679	0.766030
C	-0.011484	-4.508563	0.972873
C	1.194804	-3.873247	0.678127
PD	0.018694	0.142176	-0.398789
P	-2.269883	-0.021699	-0.158076
C	-3.337510	0.505148	-1.611496
C	-3.392579	2.037272	-1.695023
C	-2.535582	-1.840765	-0.009348
C	2.520318	-1.910200	-0.171180
P	2.301218	-0.080086	-0.175594
C	2.882553	0.502346	1.517885
C	2.368065	1.932925	1.749005
C	3.352201	0.550243	-1.596929
C	3.485111	2.077767	-1.511485
C	-2.815302	0.703173	1.493344
C	-2.255926	2.131066	1.611945
C	-4.335084	0.703236	1.690562
C	-2.150502	-0.159912	2.581180
C	-2.575384	-0.016854	-2.846874
C	-4.753097	-0.088137	-1.577980
C	4.735520	-0.111371	-1.669281
C	2.533377	0.200706	-2.856658
C	2.211881	-0.422939	2.549484
C	4.404852	0.440674	1.688598
H	-0.704030	2.695391	-0.531166
H	-2.161893	-4.334678	0.968434
H	-0.014702	-5.531308	1.349462
H	2.137930	-4.404670	0.813371
H	3.353781	-2.231411	0.466904
H	2.783619	-2.198646	-1.199538
H	-3.317598	-2.083318	0.721879
H	-2.904884	-2.191128	-0.984056
H	-2.541161	2.552763	2.586124
H	-1.158459	2.120406	1.562717
H	-2.641452	2.802479	0.836104
H	-4.561166	1.020505	2.719063
H	-4.836328	1.401580	1.011011
H	-4.768144	-0.294880	1.548075
H	-2.323573	0.311498	3.558705
H	-2.562551	-1.174888	2.618124
H	-1.067451	-0.234627	2.419967
H	-3.960740	2.318641	-2.594043
H	-3.899573	2.487124	-0.833609
H	-2.378943	2.452361	-1.809164
H	-3.146539	0.245298	-3.749784
H	-1.584054	0.454673	-2.904128
H	-2.455411	-1.108249	-2.833129
H	-5.263971	0.178482	-2.514840
H	-4.744360	-1.182928	-1.509371

H	-5.350896	0.303791	-0.749610
H	2.631102	2.247160	2.769209
H	2.799969	2.653753	1.047782
H	1.276024	1.972380	1.646598
H	4.656253	0.683329	2.731548
H	4.802799	-0.559824	1.476285
H	4.919502	1.164726	1.047204
H	2.426935	-0.037875	3.556089
H	1.123590	-0.447756	2.413050
H	2.585980	-1.451632	2.493133
H	5.250062	0.251744	-2.571150
H	5.362197	0.134046	-0.806018
H	4.668002	-1.203451	-1.747472
H	3.964911	2.440262	-2.432258
H	2.500138	2.553044	-1.430010
H	4.111102	2.390836	-0.667677
H	3.105900	0.507176	-3.744791
H	2.341100	-0.877602	-2.939905
H	1.572458	0.737208	-2.850394
O	0.120034	2.316146	-0.893087
O	-0.108556	2.444576	-2.363994

[(PCP)Pd-H] - O2 TSII SOC

73

C	1.240883	2.317465	0.787491
C	0.016949	1.616740	0.612402
C	-1.189379	2.357774	0.727629
C	-1.158169	3.746112	0.907618
C	0.054404	4.427259	1.008840
C	1.247235	3.705606	0.967625
PD	-0.000923	-0.345224	0.162130
P	-2.281430	-0.038110	0.008304
C	-3.374835	-1.153039	1.056320
C	-3.414661	-2.557264	0.436861
C	-2.516445	1.645265	0.715588
C	2.541216	1.558419	0.827964
P	2.284241	-0.084671	0.039717
C	2.858038	0.127688	-1.744024
C	2.335702	-1.048719	-2.583608
C	3.341904	-1.275523	1.035798
C	3.401384	-2.628985	0.313390
C	-2.837920	0.103617	-1.789609
C	-2.319846	-1.110939	-2.576428
C	-4.356640	0.233376	-1.950167
C	-2.148362	1.361041	-2.350399
C	-2.646018	-1.242655	2.412357
C	-4.793703	-0.607869	1.269826
C	4.753159	-0.747616	1.329077
C	2.572076	-1.459201	2.358941
C	2.193774	1.417526	-2.259108
C	4.380455	0.248079	-1.878670
H	-0.842004	-2.726011	-1.017074
H	-2.098918	4.295234	0.970267
H	0.069408	5.509573	1.134945
H	2.201609	4.223160	1.076341
H	3.373350	2.115620	0.378371
H	2.821344	1.366866	1.874285
H	-3.298060	2.210458	0.191788

H	-2.869123	1.511757	1.748524
H	-2.610350	-1.007115	-3.631459
H	-1.224352	-1.160714	-2.531895
H	-2.735405	-2.057888	-2.212216
H	-4.585381	0.453428	-3.003249
H	-4.875873	-0.694701	-1.686145
H	-4.768189	1.049675	-1.343277
H	-2.326584	1.405210	-3.433955
H	-2.537921	2.283899	-1.905827
H	-1.065381	1.327369	-2.176932
H	-3.974289	-3.226201	1.107348
H	-3.917303	-2.571512	-0.537066
H	-2.392938	-2.956445	0.349634
H	-3.213371	-1.914733	3.072887
H	-1.634692	-1.645615	2.268912
H	-2.575029	-0.267337	2.911461
H	-5.307390	-1.250944	1.999563
H	-4.789401	0.411018	1.677255
H	-5.386345	-0.608710	0.350510
H	2.597379	-0.877221	-3.637658
H	2.766958	-2.006691	-2.276143
H	1.245256	-1.128988	-2.501488
H	4.625730	0.498777	-2.921253
H	4.791145	1.041706	-1.241498
H	4.887847	-0.692498	-1.636952
H	2.402934	1.512952	-3.333904
H	1.106516	1.385680	-2.117005
H	2.577145	2.314103	-1.758566
H	5.258086	-1.457459	2.000918
H	5.363022	-0.653156	0.425582
H	4.733304	0.225265	1.835974
H	3.878896	-3.364421	0.977444
H	2.390766	-2.984321	0.076532
H	3.996150	-2.578705	-0.606311
H	3.129620	-2.162148	2.995628
H	2.470986	-0.516400	2.913222
H	1.571911	-1.866098	2.155235
O	0.062635	-2.540331	-0.696053
O	-0.094094	-2.768403	0.792280

[(PCP)Pd-H] - O2 PC SOC

73

PD	0.028434	0.350549	-0.082896
O	0.183569	2.421027	-0.420776
C	0.019718	-1.679399	0.044325
C	-1.140420	-2.421043	-0.284865
C	-1.139558	-3.819699	-0.213361
C	0.012950	-4.508205	0.169122
C	1.171183	-3.794785	0.482723
C	1.180536	-2.395437	0.426822
C	-2.379944	-1.685429	-0.729094
H	-2.046597	-4.373470	-0.461972
H	0.009604	-5.597194	0.219859
H	2.076589	-4.329419	0.774943
C	2.433719	-1.632724	0.782389
P	2.295497	0.067608	0.072496
H	2.507428	-1.512317	1.873319
H	3.347868	-2.150025	0.461560

P	-2.263079	0.067398	-0.149353
H	-3.303853	-2.179106	-0.398955
H	-2.419136	-1.645117	-1.827922
C	-3.101426	0.072418	1.540069
C	-3.200231	1.055566	-1.446762
C	-2.224475	1.144330	-2.637227
C	-4.507577	0.392902	-1.903400
C	-3.469733	2.473716	-0.925058
C	-4.611519	-0.181946	1.469639
C	-2.430746	-1.047027	2.358152
C	-2.814523	1.410206	2.240081
H	-3.149275	1.342275	3.285552
H	-1.742810	1.640880	2.222669
H	-3.340384	2.246342	1.768503
H	-5.003409	-0.289468	2.492036
H	-5.144499	0.651539	0.998104
H	-4.852622	-1.103925	0.924942
H	-2.829345	-1.012930	3.382435
H	-2.632467	-2.042921	1.947173
H	-1.342761	-0.911882	2.399196
H	-3.815186	3.100760	-1.760143
H	-4.254405	2.482237	-0.158994
H	-2.560953	2.920936	-0.505424
H	-2.704798	1.708479	-3.450480
H	-1.302060	1.658071	-2.338017
H	-1.958490	0.153284	-3.028685
H	-4.948562	1.001822	-2.706659
H	-4.341171	-0.613112	-2.307469
H	-5.243897	0.321710	-1.096742
C	3.148588	0.002788	-1.601869
C	3.155571	1.182691	1.316848
C	2.186270	1.255935	2.513578
C	4.509132	0.643720	1.800279
C	3.308184	2.587051	0.713964
C	4.672083	-0.138961	-1.515483
C	2.556447	-1.218046	-2.331017
C	2.762942	1.265300	-2.391113
H	3.588953	3.292455	1.509597
H	4.095718	2.616924	-0.048550
H	2.362652	2.914870	0.258602
H	4.895122	1.310136	2.586009
H	4.421062	-0.360063	2.234382
H	5.252962	0.608037	0.998552
H	2.593568	1.950287	3.262419
H	1.194142	1.609022	2.202783
H	2.052222	0.280351	2.998233
H	5.074858	-0.299450	-2.526426
H	5.143969	0.764045	-1.111777
H	4.972763	-0.995999	-0.899129
H	3.132698	1.168596	-3.422172
H	1.672251	1.386932	-2.420064
H	3.193739	2.174429	-1.959813
H	2.946782	-1.232044	-3.358777
H	2.832303	-2.163963	-1.850220
H	1.461347	-1.163167	-2.372092
O	-0.513599	3.224829	0.633167
H	0.209353	3.331180	1.281094

[(CNC)Au-H] SOC

55

C	0.042816	-0.169615	0.247309
N	-0.007634	-0.051719	1.593342
C	-0.293040	1.084645	2.269090
C	-0.558686	2.239477	1.526508
C	-0.517103	2.165446	0.133310
C	-0.217749	0.969726	-0.520910
AU	0.372571	-1.739742	2.705145
C	-0.279495	0.916904	3.731275
C	0.373634	-1.528640	-0.211258
C	0.593579	-2.530191	0.787348
H	0.666385	-3.032524	3.558066
H	-0.793740	3.176531	2.024182
H	-0.722713	3.058500	-0.454824
H	-0.188715	0.924602	-1.606362
C	-0.547913	1.971831	4.613815
C	-0.523429	1.761857	5.988256
C	-0.231657	0.498019	6.528525
C	0.035520	-0.552363	5.633018
C	0.020047	-0.379475	4.247343
H	-0.777974	2.966254	4.230874
H	-0.735811	2.601047	6.649377
C	-0.213403	0.310717	8.048548
H	0.263677	-1.539453	6.028634
C	0.475534	-1.863425	-1.564344
C	0.788491	-3.167572	-1.950646
C	1.008859	-4.169910	-0.998307
C	0.903798	-3.818717	0.364094
H	1.072793	-4.587689	1.117633
C	1.354215	-5.611793	-1.380660
H	0.858797	-3.393131	-3.011983
H	0.310309	-1.108626	-2.333414
C	0.119345	-1.131930	8.457216
C	0.850485	1.248644	8.659334
C	-1.602500	0.671344	8.618444
C	1.422398	-5.813251	-2.901576
C	2.727183	-5.978547	-0.776505
C	0.274052	-6.556670	-0.810317
H	0.117900	-1.208372	9.552320
H	-0.620636	-1.844781	8.069882
H	1.112333	-1.436577	8.100943
H	0.873400	1.129467	9.751349
H	1.847957	1.012672	8.265135
H	0.636086	2.301380	8.437088
H	-1.603820	0.547756	9.710137
H	-1.873908	1.710350	8.394082
H	-2.376819	0.017152	8.195982
H	0.511444	-7.597694	-1.069410
H	0.210763	-6.484720	0.282450
H	-0.712728	-6.313768	-1.226637
H	2.985253	-7.015118	-1.033800
H	3.512677	-5.319478	-1.169820
H	2.723693	-5.890796	0.316878
H	1.671418	-6.860127	-3.119128
H	0.461537	-5.589918	-3.383867
H	2.195128	-5.182455	-3.360538

[(CNC)Au-H] - O2 RC SOC

57

C	1.250489	-3.850621	0.556987
C	0.816796	-2.591751	0.955703
C	0.396418	-1.673219	-0.057312
C	0.434905	-2.055654	-1.401620
C	0.873036	-3.329067	-1.763559
C	1.288068	-4.251573	-0.795439
C	-0.082584	-0.351672	0.376278
N	-0.069241	-0.186844	1.718429
C	-0.464778	0.927771	2.374245
C	-0.925676	2.006797	1.613194
C	-0.957415	1.882287	0.223334
C	-0.540735	0.710564	-0.409974
AU	0.606207	-1.761560	2.856132
C	0.153951	-0.402825	4.374947
C	-0.347147	0.820628	3.837461
C	-0.710559	1.862918	4.700998
C	-0.587130	1.710334	6.077565
C	-0.099071	0.518198	6.638982
C	0.264206	-0.519205	5.762256
C	0.016971	0.390921	8.160885
C	-1.381973	0.571303	8.789540
C	1.775738	-5.657908	-1.151664
C	0.875267	-6.698464	-0.451530
C	0.571056	-0.974250	8.594435
C	0.962531	1.491408	8.689253
C	1.740017	-5.923949	-2.663733
C	3.230267	-5.824699	-0.659915
H	1.124608	-2.968353	3.727144
H	-1.254117	2.924161	2.094491
H	-1.315220	2.715902	-0.378853
H	-0.571297	0.625382	-1.492971
H	-1.094125	2.802048	4.301595
H	-0.878656	2.537415	6.723630
H	0.645228	-1.450819	6.174648
H	1.568846	-4.558679	1.321755
H	0.884773	-3.595790	-2.817563
H	0.115612	-1.363534	-2.181140
H	0.632769	-1.009519	9.690005
H	-0.077350	-1.798194	8.268404
H	1.579125	-1.149007	8.195754
H	1.050992	1.417229	9.781927
H	1.964952	1.383408	8.253738
H	0.591088	2.494612	8.446242
H	-1.315569	0.489217	9.883216
H	-1.809987	1.552104	8.547971
H	-2.072728	-0.201644	8.426747
H	1.223549	-7.713677	-0.686458
H	0.889775	-6.577160	0.638474
H	-0.164012	-6.601362	-0.791291
H	3.594843	-6.832544	-0.902075
H	3.890043	-5.092878	-1.145002
H	3.307479	-5.687040	0.425660
H	2.095958	-6.943302	-2.862488
H	0.721846	-5.839730	-3.066261
H	2.388345	-5.229177	-3.213931
O	-2.361137	-3.178408	0.099703
O	-2.013081	-4.350667	0.176369

[(CNC)Au-H] - O2 TSI SOC

57

C	-3.093072	-0.668406	0.091106
C	-2.026089	0.212932	0.054028
C	-2.290249	1.614253	-0.018696
C	-3.613438	2.062150	-0.051062
C	-4.668788	1.149074	-0.012839
C	-4.435406	-0.230679	0.056362
C	-1.133369	2.518641	-0.065175
N	0.060857	1.890396	-0.027630
C	1.283041	2.458875	-0.075126
C	1.325775	3.855741	-0.158103
C	0.124706	4.565477	-0.191171
C	-1.109361	3.915402	-0.146900
AU	0.015462	-0.181679	0.073665
C	2.083245	0.112260	0.037977
C	2.398895	1.501025	-0.035095
C	3.738142	1.911964	-0.069388
C	4.760525	0.970947	-0.032805
C	4.484828	-0.405273	0.038823
C	3.136408	-0.802909	0.073721
C	5.642268	-1.406939	0.074409
C	6.520621	-1.117979	1.311307
C	-5.564730	-1.261384	0.102375
C	-5.459926	-2.057957	1.421469
C	5.156562	-2.861717	0.152323
C	6.489299	-1.242665	-1.206530
C	-6.953862	-0.610563	0.032878
C	-5.415083	-2.227589	-1.092866
H	-0.092795	-2.111513	-0.166805
H	2.280009	4.374443	-0.196945
H	0.151164	5.651884	-0.256292
H	-2.038398	4.478838	-0.177153
H	3.990512	2.970933	-0.124884
H	5.792283	1.318523	-0.061080
H	2.902571	-1.864623	0.129757
H	-2.883945	-1.742524	0.141765
H	-5.686640	1.530728	-0.039181
H	-3.835205	3.127979	-0.108815
H	6.024656	-3.533237	0.175318
H	4.566786	-3.045555	1.060095
H	4.545807	-3.133630	-0.718739
H	7.329417	-1.950540	-1.193522
H	5.883423	-1.442258	-2.100511
H	6.900584	-0.229282	-1.291980
H	7.361019	-1.824735	1.347342
H	6.932798	-0.101634	1.285975
H	5.937496	-1.227560	2.235476
H	-6.258365	-2.811174	1.469136
H	-4.496804	-2.576994	1.502146
H	-5.562434	-1.390198	2.287285
H	-6.215651	-2.979553	-1.068536
H	-5.482355	-1.682204	-2.043742
H	-4.453294	-2.754272	-1.068552
H	-7.723750	-1.392539	0.063611
H	-7.128922	0.065183	0.880505
H	-7.089382	-0.042291	-0.896972
O	-0.342673	-3.046217	0.311105

O	-1.517572	-3.488214	-0.149664

[(CNC)Au-H] - O2 INT SOC

57

C	-3.123048	-0.668559	0.086921
C	-2.069055	0.225103	0.044698
C	-2.333021	1.621029	-0.010565
C	-3.657556	2.069061	-0.017963
C	-4.706840	1.148757	0.026212
C	-4.465079	-0.231620	0.076652
C	-1.167096	2.503888	-0.063340
N	0.023795	1.847545	-0.038911
C	1.248303	2.436607	-0.096178
C	1.290844	3.830296	-0.175868
C	0.095962	4.548397	-0.196061
C	-1.135584	3.898065	-0.141426
AU	-0.021547	-0.155050	0.040960
C	2.052377	0.114863	0.004530
C	2.371787	1.496359	-0.063067
C	3.714930	1.896551	-0.085377
C	4.725447	0.942668	-0.037423
C	4.436871	-0.431752	0.036704
C	3.084529	-0.816722	0.057587
C	5.582894	-1.444825	0.098605
C	6.438864	-1.158809	1.352109
C	-5.589049	-1.268475	0.124501
C	-5.453867	-2.091671	1.424226
C	5.079656	-2.893861	0.174002
C	6.457045	-1.296238	-1.165573
C	-6.983084	-0.625689	0.093271
C	-5.453497	-2.209712	-1.092069
H	0.389453	-2.495663	-0.726529
H	2.248439	4.341515	-0.221769
H	0.125591	5.634659	-0.257854
H	-2.065128	4.460363	-0.161713
H	3.979114	2.952688	-0.136652
H	5.761143	1.278939	-0.054925
H	2.836801	-1.875640	0.124882
H	-2.866073	-1.732607	0.132110
H	-5.727000	1.525518	0.019065
H	-3.884124	3.134591	-0.058231
H	5.939704	-3.574713	0.214718
H	4.472443	-3.067797	1.072245
H	4.481211	-3.162883	-0.706455
H	7.290108	-2.011658	-1.130247
H	5.868053	-1.496503	-2.070671
H	6.879688	-0.287424	-1.249395
H	7.269380	-1.875669	1.409859
H	6.864164	-0.147745	1.328672
H	5.835253	-1.255126	2.264452
H	-6.245096	-2.852713	1.470967
H	-4.484479	-2.602590	1.475050
H	-5.545683	-1.443025	2.305692
H	-6.245978	-2.970450	-1.068026
H	-5.543430	-1.646604	-2.030766
H	-4.485150	-2.725104	-1.094316
H	-7.748031	-1.412548	0.125745
H	-7.145315	0.034998	0.955269

H	-7.139941	-0.042611	-0.824104
O	0.078295	-2.233776	0.165636
O	-1.148982	-3.022818	0.314210

[(CNC)Au-H] - O2 TSII SOC

57

C	-3.079411	0.772919	-0.036189
C	-2.024122	-0.125580	-0.016357
C	-2.303322	-1.520634	-0.014892
C	-3.631667	-1.959134	-0.013289
C	-4.673836	-1.032469	-0.015966
C	-4.424092	0.347661	-0.031951
C	-1.147123	-2.412853	-0.036306
N	0.055416	-1.765701	-0.057229
C	1.274414	-2.382350	-0.080648
C	1.293131	-3.777777	-0.089478
C	0.089933	-4.480955	-0.070434
C	-1.130479	-3.808574	-0.042700
AU	0.031078	0.201424	-0.033464
C	2.089825	-0.077386	-0.057858
C	2.405892	-1.459898	-0.085229
C	3.749712	-1.860646	-0.113295
C	4.756967	-0.903068	-0.112167
C	4.466623	0.474480	-0.086680
C	3.115751	0.861656	-0.061364
C	5.613150	1.489071	-0.089153
C	6.456066	1.293840	-1.368439
C	-5.544927	1.389936	-0.056153
C	-5.415439	2.234348	-1.342944
C	5.109773	2.939587	-0.055348
C	6.500129	1.250569	1.152299
C	-6.939919	0.748222	-0.032348
C	-5.408182	2.308833	1.177299
H	-0.832208	2.577340	0.707694
H	2.245204	-4.301253	-0.107717
H	0.103570	-5.569099	-0.075469
H	-2.068741	-4.356412	-0.025793
H	4.014347	-2.917819	-0.136495
H	5.793301	-1.237228	-0.133280
H	2.855732	1.918720	-0.047837
H	-2.867679	1.843534	-0.075846
H	-5.696439	-1.401607	-0.012860
H	-3.865244	-3.023796	-0.014095
H	5.969605	3.622013	-0.056818
H	4.492443	3.176429	-0.931904
H	4.520589	3.144343	0.848386
H	7.331977	1.968282	1.161618
H	5.919636	1.382681	2.075206
H	6.924894	0.239027	1.157734
H	7.288240	2.011121	-1.381830
H	6.878484	0.282882	-1.424028
H	5.843702	1.458360	-2.265154
H	-6.209953	2.992531	-1.375096
H	-4.449700	2.752515	-1.390995
H	-5.507067	1.599461	-2.234268
H	-6.204679	3.065494	1.170338
H	-5.491259	1.727944	2.105557
H	-4.444187	2.832225	1.187594

H	-7.702903	1.537348	-0.047754
H	-7.106148	0.104449	-0.906160
H	-7.095163	0.148694	0.874514
O	0.065586	2.314866	0.415031
O	-0.080684	2.352652	-1.104865

[(CNC)Au-H] - O2 PC SOC

57

C	-3.090874	0.647143	-0.073577
C	-2.005329	-0.225348	-0.047426
C	-2.259562	-1.621961	0.026836
C	-3.580028	-2.091944	0.065055
C	-4.640742	-1.194597	0.031929
C	-4.420487	0.192362	-0.035403
AU	0.057604	0.154799	-0.087333
N	0.093963	-1.852235	0.028154
C	1.313190	-2.455411	0.052080
C	1.352685	-3.849573	0.125536
C	0.155428	-4.562367	0.168208
C	-1.072272	-3.903221	0.138886
C	-1.094868	-2.508046	0.066430
C	2.441326	-1.522684	-0.008220
C	2.122002	-0.138866	-0.083613
C	3.149064	0.789326	-0.146566
C	4.506150	0.404946	-0.137692
C	4.797177	-0.964682	-0.063334
C	3.782194	-1.920059	0.001338
C	5.595267	1.478323	-0.208066
C	5.418529	2.290694	-1.509433
C	-5.615899	1.148647	-0.064186
C	-6.492272	0.825278	-1.293872
C	-6.447025	0.955173	1.223012
C	-5.184878	2.620253	-0.147921
C	5.451778	2.419676	1.007739
C	7.009240	0.879136	-0.196311
O	0.294413	2.135704	-0.208222
H	-2.003821	-4.461604	0.170970
H	0.179732	-5.649169	0.224050
H	2.308777	-4.365486	0.146909
H	-3.786048	-3.160818	0.121189
H	-5.657325	-1.584605	0.062218
H	-2.884323	1.713784	-0.120435
H	2.891463	1.848805	-0.204690
H	5.830193	-1.303841	-0.054341
H	4.048193	-2.975646	0.058288
H	-6.077048	3.259675	-0.166214
H	-4.577748	2.915821	0.717705
H	-4.607787	2.823054	-1.059912
H	-7.358925	1.499885	-1.324861
H	-5.920618	0.954806	-2.222685
H	-6.866152	-0.205755	-1.263826
H	-7.314519	1.629407	1.214494
H	-6.817295	-0.073520	1.314069
H	-5.843086	1.181008	2.112127
H	6.222147	3.202125	0.968758
H	4.470407	2.909635	1.025042
H	5.572069	1.862517	1.946497
H	6.188724	3.071843	-1.571524

H	5.514674	1.640200	-2.389020
H	4.436670	2.778070	-1.551004
H	7.747738	1.689494	-0.252010
H	7.202564	0.312171	0.724093
H	7.175605	0.214266	-1.054201
O	-0.940912	2.927389	-0.300139
H	-1.054071	2.983253	-1.270336

[(PCP)Au-H]+ - model SOC

35

C	1.112737	3.986307	-0.051471
C	1.123789	2.603954	0.144677
C	-0.053579	1.861549	0.033395
C	-1.261410	2.502529	-0.266694
C	-1.275063	3.895464	-0.479831
C	-0.082187	4.638643	-0.377953
C	-2.561397	1.734459	-0.332934
P	-3.754249	2.676558	-1.367782
C	-5.428922	2.140225	-0.945080
AU	-3.083070	4.869107	-0.955423
P	-1.823001	6.742043	-0.381654
C	-2.020299	8.296715	-1.284680
C	-0.113281	6.123630	-0.657300
C	-3.444981	2.208095	-3.091828
C	-1.970709	7.114776	1.387021
H	2.037911	4.555902	0.037542
H	2.058873	2.100437	0.387604
H	-0.033698	0.783257	0.191668
H	-2.439312	0.715102	-0.721780
H	-3.014683	1.651951	0.666620
H	0.124498	6.325404	-1.712793
H	0.601445	6.690982	-0.046984
H	-6.144019	2.654711	-1.595672
H	-5.516946	1.055772	-1.084508
H	-5.638697	2.398399	0.098183
H	-4.096348	2.790133	-3.752038
H	-2.398863	2.421780	-3.336389
H	-3.645944	1.137911	-3.221705
H	-1.268984	7.915075	1.650879
H	-2.994729	7.431212	1.610847
H	-1.737283	6.212079	1.961870
H	-1.253416	9.011632	-0.961626
H	-1.921446	8.107330	-2.358595
H	-3.015052	8.706148	-1.079638
H	-4.482319	5.622684	-1.325649

[(PCP)Au-H]+ - model O2 RC SOC

37

C	0.884615	3.998502	0.257108
C	0.811521	2.628920	0.519265
C	-0.369205	1.922872	0.280743
C	-1.497453	2.588938	-0.212558
C	-1.424525	3.968349	-0.490497
C	-0.226644	4.674284	-0.260662
C	-2.805955	1.861484	-0.419271
P	-3.827771	2.798735	-1.627559

C	-5.562002	2.354595	-1.368350
AU	-3.113303	4.981949	-1.241963
P	-1.860068	6.836972	-0.598455
C	-1.876499	8.345277	-1.595267
C	-0.157204	6.142562	-0.612816
C	-3.354678	2.224252	-3.280438
C	-2.221641	7.305382	1.115467
H	1.812210	4.539242	0.445667
H	1.683135	2.106726	0.912320
H	-0.415891	0.854242	0.490450
H	-2.672461	0.825508	-0.757022
H	-3.383048	1.825476	0.517270
H	0.225792	6.278625	-1.635445
H	0.494729	6.715490	0.059580
H	-6.177688	2.861147	-2.118860
H	-5.683917	1.268675	-1.462865
H	-5.875793	2.674210	-0.369122
H	-3.902225	2.795804	-4.036896
H	-2.279258	2.381415	-3.417153
H	-3.589845	1.157688	-3.377997
H	-1.534460	8.099684	1.430574
H	-3.254822	7.659958	1.190184
H	-2.091338	6.428199	1.758402
H	-1.129264	9.050385	-1.210959
H	-1.648634	8.093350	-2.636251
H	-2.872352	8.798049	-1.541797
H	-4.421563	5.766661	-1.820507
O	-5.217667	4.893475	1.920497
O	-4.180917	4.270416	1.730464

[(PCP)Au-H]+ - model O2 TSI SOC

37

C	1.110544	2.538783	0.126425
C	-0.124259	1.892018	-0.026614
C	-1.221447	2.685463	-0.369592
C	-1.160693	4.067111	-0.564063
C	0.083898	4.685517	-0.376323
C	1.203533	3.921971	-0.040449
C	-0.289408	0.407470	0.208181
P	-1.790664	-0.214695	-0.662711
C	-2.351754	-1.716874	0.171410
AU	-3.107440	1.716822	-0.639020
P	-3.917699	3.911285	-0.572064
C	-5.227721	4.459510	-1.693809
C	-2.383736	4.837835	-1.004851
C	-4.379762	4.415370	1.107639
C	-1.285607	-0.668111	-2.343278
O	-5.452733	-0.167272	-1.621630
O	-4.852928	-1.288447	-2.075821
H	-4.702361	0.533553	-1.464628
H	0.174187	5.764089	-0.505764
H	2.166652	4.413192	0.090919
H	1.994762	1.956977	0.386749
H	0.593484	-0.164900	-0.103248
H	-0.448689	0.208160	1.278306
H	-2.387451	4.946596	-2.099681
H	-2.419046	5.847623	-0.576602
H	-5.345681	5.547848	-1.622759

H	-4.966024	4.180529	-2.719842
H	-6.166435	3.970053	-1.412970
H	-4.583843	5.492536	1.125352
H	-5.271596	3.863135	1.421528
H	-3.554358	4.183431	1.789586
H	-3.240786	-2.088777	-0.351706
H	-1.560939	-2.476513	0.142140
H	-2.607601	-1.482561	1.209814
H	-0.533624	-1.464993	-2.298993
H	-2.167038	-1.014258	-2.893555
H	-0.866281	0.211909	-2.842953

[(PCP)Au-H]+ - model O2 INT SOC

37

C	1.280942	-2.475324	-0.031759
C	0.047051	-1.833410	0.127532
C	-1.049088	-2.594539	0.576891
C	-0.929837	-3.968622	0.853281
C	0.311434	-4.585010	0.657997
C	1.407731	-3.840183	0.224808
C	-0.117701	-0.367463	-0.194745
P	-1.542850	0.274451	0.766504
C	-2.214583	1.747240	-0.028548
AU	-2.856484	-1.675155	0.839512
O	-4.638061	-0.530602	1.148692
O	-4.259831	0.395447	2.263196
C	-2.113288	-4.742227	1.380178
P	-3.644806	-3.893567	0.841765
C	-5.003274	-4.340895	1.945554
C	-4.045815	-4.474381	-0.824497
C	-0.944465	0.712274	2.414251
H	-5.332758	-1.080153	1.565007
H	0.417261	-5.650556	0.858396
H	2.371467	-4.327959	0.086796
H	2.142514	-1.899407	-0.367689
H	0.792199	0.210755	0.007389
H	-0.367201	-0.232644	-1.257684
H	-2.121354	-4.734311	2.480187
H	-2.113686	-5.789877	1.055275
H	-5.924706	-3.859662	1.599617
H	-5.140966	-5.429048	1.936461
H	-4.772928	-4.004919	2.961859
H	-4.270000	-5.547135	-0.793251
H	-4.914001	-3.924971	-1.203170
H	-3.188326	-4.291370	-1.481046
H	-3.070558	2.078443	0.568857
H	-1.444819	2.528258	-0.064595
H	-2.541683	1.499495	-1.043492
H	-0.271789	1.575271	2.344334
H	-1.813983	0.950158	3.035353
H	-0.409155	-0.143729	2.839619

[(PCP)Au-H]+ - model O2 TSII SOC

37

C	1.139800	2.445287	0.237858
C	-0.041196	1.739351	-0.019791

C	-1.179313	2.463683	-0.426187
C	-1.149569	3.863892	-0.567590
C	0.041068	4.539181	-0.276280
C	1.177289	3.833976	0.118374
C	-0.109511	0.241809	0.157223
P	-1.469071	-0.383168	-0.897539
C	-2.091060	-1.957516	-0.273773
AU	-2.905295	1.463793	-0.819566
O	-4.312232	0.328914	-2.486351
O	-4.748590	0.242526	-1.030266
C	-2.370910	4.604624	-1.051475
P	-3.849624	3.607372	-0.634082
C	-5.205381	4.041017	-1.746915
C	-4.344228	4.027616	1.055595
C	-0.826421	-0.635603	-2.567707
H	-5.556521	0.794602	-1.029985
H	0.074203	5.624051	-0.370391
H	2.099934	4.370623	0.333727
H	2.030420	1.897803	0.544110
H	0.839725	-0.254795	-0.077284
H	-0.372545	-0.012666	1.194910
H	-2.357055	4.698518	-2.147700
H	-2.447067	5.615101	-0.632029
H	-6.099704	3.472246	-1.468788
H	-5.421992	5.113118	-1.664403
H	-4.922848	3.792819	-2.775045
H	-4.645531	5.080996	1.099803
H	-5.180993	3.390454	1.361065
H	-3.496050	3.853800	1.726615
H	-2.925983	-2.277130	-0.907176
H	-1.293843	-2.710058	-0.306614
H	-2.442290	-1.828302	0.754956
H	-0.098395	-1.455339	-2.565210
H	-1.664555	-0.874717	-3.230677
H	-0.347203	0.288724	-2.908029

[(PCP)Au-H]+ - model O2 PC SOC

37

C	1.396741	-3.833605	0.192065
C	1.280461	-2.473599	-0.094762
C	0.057213	-1.816228	0.080200
C	-1.046374	-2.538037	0.573927
C	-0.926860	-3.908633	0.880217
C	0.299404	-4.549760	0.670265
C	-0.100844	-0.359175	-0.277105
P	-1.509309	0.330961	0.671466
C	-0.882114	0.878337	2.280184
AU	-2.851930	-1.584932	0.882298
P	-3.637561	-3.792923	0.964222
C	-4.112008	-4.398686	-0.674431
C	-2.102803	-4.658248	1.461102
C	-2.121231	1.785876	-0.208301
O	-4.720714	-0.817973	1.286526
O	-4.733438	0.660191	1.360498
C	-4.970468	-4.175542	2.120032
H	-5.154898	0.877801	0.505686
H	0.395779	-5.611724	0.894702
H	2.349917	-4.338976	0.042721

H	2.141030	-1.917646	-0.466049
H	0.811205	0.224155	-0.098155
H	-0.360360	-0.247237	-1.340650
H	-2.072494	-4.634813	2.560727
H	-2.130722	-5.710749	1.152842
H	-2.912295	2.254168	0.384786
H	-1.293408	2.491605	-0.350832
H	-2.521170	1.483331	-1.181687
H	-1.719687	1.225370	2.894548
H	-0.393398	0.034301	2.778979
H	-0.163421	1.694036	2.137442
H	-5.182367	-5.251315	2.099785
H	-4.672850	-3.871411	3.128870
H	-5.866138	-3.618430	1.823515
H	-4.357851	-5.465556	-0.613819
H	-4.981750	-3.837520	-1.031978
H	-3.275696	-4.247697	-1.365484

[(PCP)Au-OHO]+ - O2 model RCO2 SOC

39

C	-4.341891	1.428548	0.233276
C	-4.097140	0.103662	-0.125467
C	-2.784670	-0.367323	-0.244528
C	-1.725856	0.516920	0.034919
C	-1.960039	1.853298	0.407757
C	-3.284223	2.299552	0.490336
C	-2.506121	-1.779190	-0.698561
P	-0.845071	-2.266793	-0.103863
C	-1.017645	-2.913650	1.576937
AU	0.203612	-0.157876	-0.128098
P	0.711685	2.137177	-0.055794
C	0.825607	2.827730	-1.722278
C	-0.810677	2.771578	0.742095
C	-0.166302	-3.565487	-1.157023
C	2.155034	2.659760	0.887761
O	3.456176	-0.918161	1.697355
O	4.240967	-0.013617	1.275576
O	2.197031	-0.901293	-0.448336
O	3.076122	0.084718	-0.992298
H	2.619222	-1.082629	0.492376
H	-3.481867	3.334409	0.767541
H	-5.367381	1.785971	0.312539
H	-4.927524	-0.571862	-0.327555
H	-3.266744	-2.490988	-0.355041
H	-2.473450	-1.832212	-1.796730
H	-0.619251	2.768349	1.825300
H	-1.003249	3.809843	0.445732
H	2.235763	3.753102	0.854270
H	2.052331	2.324198	1.924692
H	3.037056	2.195306	0.433866
H	0.963859	3.913686	-1.660758
H	1.680100	2.368647	-2.231310
H	-0.094161	2.598423	-2.271054
H	0.828893	-3.840984	-0.790912
H	-0.823536	-4.442956	-1.126697
H	-0.086015	-3.193921	-2.183709
H	-0.027641	-3.136405	1.988463

```
H         -1.507206   -2.158217    2.200820
H         -1.622152   -3.828117    1.554255
```

[(PCP)Au-OHO]+ - O2 model TSsub SOC

39

```
C         -2.658526   -0.139311    0.214632
C         -1.476234   -0.828273   -0.102639
C         -1.473399   -2.171856   -0.512917
C         -2.702845   -2.837048   -0.589655
C         -3.889073   -2.167402   -0.291479
C         -3.871357   -0.829121    0.100458
AU         0.320815    0.172022    0.041205
P         -1.082754    2.056733    0.012689
C         -1.432496    2.578266   -1.683889
C         -0.182842   -2.856940   -0.887082
P          1.192165   -2.002999   -0.030363
C          1.360060   -2.703613    1.627656
C         -2.604580    1.291843    0.687968
C          2.738549   -2.259478   -0.925121
C         -0.636462    3.511235    0.980388
O          2.213204    0.904728    1.254778
O          1.830415    1.746056    2.292419
H          2.625882    1.509067    0.531051
H         -2.724552   -3.882807   -0.893960
H         -4.838722   -2.694686   -0.365959
H         -4.802083   -0.312780    0.332075
H         -3.493914    1.865963    0.401681
H         -2.517286    1.333275    1.783889
H          0.009029   -2.755498   -1.965643
H         -0.188001   -3.927572   -0.650227
H          2.966613   -3.331470   -0.966532
H          2.642673   -1.858813   -1.939432
H          3.545261   -1.736224   -0.399635
H          1.674351   -3.751281    1.551666
H          2.108199   -2.131528    2.186763
H          0.395320   -2.639383    2.142215
H          0.251935    3.974885    0.537818
H         -1.469824    4.224462    0.967237
H         -0.407338    3.206766    2.006131
H         -0.511646    2.947005   -2.148088
H         -1.804793    1.719408   -2.252500
H         -2.186524    3.374112   -1.672821
O          1.933695    1.420001   -1.450606
O          2.958182    1.989291   -0.909573
```

[(PCP)Au-OHO]+ - O2 model PCrad SOC

39

```
C          4.213271   -1.384664    0.646704
C          4.004349   -0.062904    0.253381
C          2.712846    0.393727   -0.033375
C          1.635058   -0.498897    0.111830
C          1.833273   -1.830610    0.520666
C          3.139393   -2.265666    0.773118
C          2.471300    1.802459   -0.519403
P          0.745731    2.264562   -0.114513
C          0.721481    2.877544    1.588804
```

AU	-0.275409	0.154022	-0.280049
P	-0.736236	-2.148176	-0.318415
C	-0.571178	-2.848817	-1.976776
C	0.652083	-2.751564	0.714299
C	0.185464	3.588229	-1.206472
C	-2.308215	-2.690637	0.383535
O	-3.102423	0.990754	1.846511
O	-1.948889	0.484593	2.311625
O	-2.266752	0.830024	-0.627311
O	-2.930567	0.238106	-1.616123
H	-2.985854	0.983345	0.833644
H	3.310858	-3.296590	1.080814
H	5.223763	-1.731943	0.856584
H	4.848151	0.619457	0.156989
H	3.177581	2.524756	-0.092034
H	2.562326	1.855704	-1.614357
H	0.302222	-2.714763	1.756930
H	0.887973	-3.797300	0.482414
H	-2.345574	-3.786454	0.404665
H	-2.406696	-2.294139	1.399544
H	-3.124748	-2.308592	-0.239005
H	-0.704141	-3.936203	-1.932083
H	-1.333047	-2.407442	-2.628499
H	0.423460	-2.609197	-2.367868
H	-0.847051	3.850156	-0.950041
H	0.828640	4.467448	-1.078360
H	0.226364	3.244004	-2.244970
H	-0.311687	3.076890	1.891851
H	1.149272	2.114042	2.247426
H	1.310849	3.799748	1.654335

[(PCP)Pd-OHO] - model SOC

37

C	-4.305620	1.424774	0.250562
C	-4.058313	0.104861	-0.129412
C	-2.744443	-0.347842	-0.259354
C	-1.668884	0.516189	-0.019034
C	-1.907507	1.853033	0.384284
C	-3.245808	2.300183	0.520483
C	-0.244265	0.049007	-0.198590
P	0.816950	1.132279	0.849055
C	2.517994	0.969121	0.213775
PD	-0.362675	3.103289	0.763682
O	1.266565	4.536799	1.116947
O	0.992957	5.767355	0.290762
C	-3.512159	3.716907	0.974483
P	-1.996906	4.700216	0.605303
C	-1.999438	6.152257	1.701199
C	0.848766	0.315961	2.482454
C	-2.236708	5.366678	-1.073026
H	1.111541	4.874751	2.019633
H	-5.332564	1.778938	0.349422
H	-4.890197	-0.571298	-0.325493
H	-2.550522	-1.378791	-0.558120
H	-0.110332	-1.015426	0.035936
H	0.084265	0.203117	-1.238021
H	-3.648329	3.749734	2.066644
H	-4.414985	4.145524	0.519357

H	-2.874765	6.786908	1.513409
H	-1.999568	5.822582	2.746193
H	-1.073571	6.701726	1.489836
H	-3.099116	6.044345	-1.103190
H	-1.323694	5.903009	-1.354193
H	-2.394868	4.534594	-1.768453
H	3.196848	1.548619	0.850020
H	2.831909	-0.082487	0.211130
H	2.562514	1.369362	-0.805310
H	1.438043	0.918522	3.182782
H	-0.178171	0.243279	2.858083
H	1.285978	-0.687333	2.403063

[(PCP)Pd-OHO] - O2 model RCO2 SOC

39

C	-4.325617	1.420494	0.236047
C	-4.072629	0.102275	-0.145197
C	-2.757070	-0.362971	-0.263730
C	-1.671600	0.497240	0.032600
C	-1.939712	1.831026	0.426775
C	-3.263198	2.281489	0.513421
C	-2.475313	-1.772614	-0.724901
P	-0.801603	-2.221137	-0.103622
C	-1.080920	-2.927646	1.555475
PD	0.237038	-0.167715	-0.135932
P	0.709185	2.078422	-0.071577
C	0.766901	2.883305	-1.706561
C	-0.788768	2.747159	0.765871
C	-0.214609	-3.615589	-1.118758
C	2.156760	2.713500	0.825960
O	3.469878	-0.879113	1.752997
O	4.283670	0.002337	1.307382
O	2.278574	-0.926815	-0.439771
O	3.197855	0.026755	-0.977977
H	2.658170	-1.075335	0.515192
H	-3.460733	3.312699	0.808584
H	-5.352065	1.777463	0.316403
H	-4.902435	-0.570783	-0.364162
H	-3.242816	-2.489511	-0.404022
H	-2.421070	-1.815483	-1.823650
H	-0.579174	2.717744	1.846320
H	-0.983912	3.793995	0.498237
H	2.196206	3.808833	0.775182
H	2.100616	2.392554	1.872051
H	3.050730	2.274672	0.369159
H	0.862752	3.971094	-1.599370
H	1.624810	2.489306	-2.262868
H	-0.152001	2.644203	-2.253601
H	0.760784	-3.947605	-0.744634
H	-0.923632	-4.451998	-1.076000
H	-0.099299	-3.282420	-2.156114
H	-0.119328	-3.197174	2.006367
H	-1.564732	-2.170891	2.183039
H	-1.718049	-3.818571	1.491239

[(PCP)Pd-OHO] - O2 model TSsub SOC

39

C	-2.621092	-0.146036	0.221244
C	-1.387811	-0.783304	-0.052144
C	-1.391374	-2.108207	-0.550902
C	-2.605901	-2.775165	-0.752953
C	-3.817585	-2.135359	-0.488874
C	-3.824223	-0.824166	-0.011053
PD	0.373497	0.198584	0.234177
P	-1.031419	2.035365	0.222482
C	-1.373981	2.704147	-1.439546
C	-0.075521	-2.774471	-0.871483
P	1.199522	-1.949652	0.165891
C	1.191081	-2.845251	1.753962
C	-2.612470	1.261219	0.763932
C	2.822950	-2.302979	-0.577639
C	-0.771779	3.492732	1.279100
O	2.345229	1.143792	0.658578
O	2.233965	2.317133	1.492942
H	2.561526	1.499100	-0.255225
H	-2.600301	-3.799268	-1.127870
H	-4.758399	-2.658616	-0.657559
H	-4.771207	-0.322756	0.191774
H	-3.488051	1.845826	0.452525
H	-2.592376	1.253037	1.864695
H	0.207259	-2.589107	-1.919223
H	-0.096139	-3.861681	-0.720615
H	2.993399	-3.385008	-0.640569
H	2.863394	-1.863762	-1.580454
H	3.605817	-1.847804	0.039885
H	1.465006	-3.896954	1.602923
H	1.903338	-2.375462	2.441339
H	0.187025	-2.781849	2.188078
H	0.172022	3.967319	0.990992
H	-1.606438	4.197264	1.171991
H	-0.687329	3.166848	2.321260
H	-0.465838	3.169030	-1.838734
H	-1.666436	1.879706	-2.099114
H	-2.178895	3.448176	-1.394211
O	1.503660	0.916957	-2.123362
O	2.606971	1.537654	-1.941642

[(PCP)Pd-OHO] - O2 model PCrad SOC

39

C	4.178065	-1.359493	0.574435
C	3.935896	-0.045934	0.168656
C	2.627847	0.392062	-0.074070
C	1.538334	-0.491710	0.114193
C	1.795074	-1.815294	0.544962
C	3.111747	-2.241226	0.758704
C	2.355598	1.801013	-0.545824
P	0.625784	2.189983	-0.052030
C	0.734321	2.760168	1.674053
PD	-0.380245	0.125544	-0.248190
P	-0.762540	-2.149197	-0.263099
C	-0.581091	-2.996503	-1.866188
C	0.628223	-2.741605	0.786707
C	0.084932	3.641389	-1.008234

C	-2.300297	-2.788821	0.467251
O	-2.471648	1.534719	1.603584
O	-1.665154	0.353088	1.671995
O	-2.272985	0.757115	-0.978801
O	-2.339094	0.718620	-2.288245
H	-3.041950	1.344402	0.824864
H	3.302004	-3.266473	1.078657
H	5.199771	-1.696310	0.748525
H	4.769933	0.642920	0.028841
H	3.074441	2.531367	-0.150691
H	2.392988	1.858262	-1.644701
H	0.281957	-2.660344	1.829113
H	0.868235	-3.796719	0.600297
H	-2.248069	-3.876593	0.601039
H	-2.455901	-2.293858	1.432430
H	-3.139765	-2.541496	-0.192766
H	-0.674008	-4.082847	-1.742295
H	-1.355815	-2.637027	-2.553082
H	0.402110	-2.753554	-2.284757
H	-0.921748	3.924969	-0.679170
H	0.769402	4.484643	-0.851178
H	0.050844	3.386095	-2.072895
H	-0.279543	2.926212	2.053500
H	1.208093	1.972965	2.271003
H	1.321493	3.684480	1.739422
